# Association Fields via Cuspless Sub-Riemannian Geodesics in SE(2)

**DOI:** 10.1007/s10851-013-0475-y

**Published:** 2013-12-06

**Authors:** R. Duits, U. Boscain, F. Rossi, Y. Sachkov

**Affiliations:** 1 IST/e, Eindhoven University of Technology, Den Dolech 2, 5600 MB Eindhoven, The Netherlands; 2 École Polytechnique Paris, CMAP, Route de Saclay, 91128 Palaiseau Cedex, France; 3 Aix-Marseille University, LSIS, 13013 Marseille, France; 4 Program Systems Institute, Russian Academy of Sciences, Pereslavl-Zalessky, 152140 Russia

**Keywords:** Sub-Riemannian geometric control, Association fields, Pontryagin’s maximum principle, Boundary value problem, Geodesics in roto-translation space

## Abstract

To model association fields that underly perceptional organization (gestalt) in psychophysics we consider the problem **P**_curve_ of minimizing $\int _{0}^{\ell} \sqrt{\xi^{2} +\kappa^{2}(s)} {\rm d}s $ for a planar curve having fixed initial and final positions and directions. Here *κ*(*s*) is the curvature of the curve with free total length *ℓ*. This problem comes from a model of geometry of vision due to Petitot (in J. Physiol. Paris 97:265–309, 2003; Math. Inf. Sci. Humaines 145:5–101, 1999), and Citti & Sarti (in J. Math. Imaging Vis. 24(3):307–326, 2006). In previous work we proved that the range $\mathcal{R} \subset\mathrm{SE}(2)$ of the exponential map of the underlying geometric problem formulated on SE(2) consists of precisely those end-conditions (*x*_fin_,*y*_fin_,*θ*_fin_) that can be connected by a globally minimizing geodesic starting at the origin (*x*_in_,*y*_in_,*θ*_in_)=(0,0,0). From the applied imaging point of view it is relevant to analyze the sub-Riemannian geodesics and $\mathcal{R}$ in detail. In this article we

- show that $\mathcal{R}$ is contained in half space *x*≥0 and (0,*y*_fin_)≠(0,0) is reached with angle *π*,
- show that the boundary $\partial\mathcal{R}$ consists of endpoints of minimizers either starting or ending in a cusp,
- analyze and plot the cones of reachable angles *θ*_fin_ per spatial endpoint (*x*_fin_,*y*_fin_),
- relate the endings of association fields to $\partial\mathcal {R}$ and compute the length towards a cusp,
- analyze the exponential map both with the common arc-length parametrization *t* in the sub-Riemannian manifold $(\mathrm{SE}(2),\mathrm{Ker}(-\sin\theta{\rm d}x +\cos\theta {\rm d}y), \mathcal{G}_{\xi}:=\xi^{2}(\cos\theta{\rm d}x+ \sin\theta {\rm d}y) \otimes(\cos\theta{\rm d}x+ \sin\theta{\rm d}y) + {\rm d}\theta \otimes{\rm d}\theta)$ and with spatial arc-length parametrization *s* in the plane $\mathbb{R}^{2}$. Surprisingly, *s*-parametrization simplifies the exponential map, the curvature formulas, the cusp-surface, and the boundary value problem,
- present a novel efficient algorithm solving the boundary value problem,
- show that sub-Riemannian geodesics solve Petitot’s circle bundle model (cf. Petitot in J. Physiol. Paris 97:265–309, [2003]),
- show a clear similarity with association field lines and sub-Riemannian geodesics.

show that $\mathcal{R}$ is contained in half space *x*≥0 and (0,*y*_fin_)≠(0,0) is reached with angle *π*,

show that the boundary $\partial\mathcal{R}$ consists of endpoints of minimizers either starting or ending in a cusp,

relate the endings of association fields to $\partial\mathcal {R}$ and compute the length towards a cusp,

analyze the exponential map both with the common arc-length parametrization *t* in the sub-Riemannian manifold $(\mathrm{SE}(2),\mathrm{Ker}(-\sin\theta{\rm d}x +\cos\theta {\rm d}y), \mathcal{G}_{\xi}:=\xi^{2}(\cos\theta{\rm d}x+ \sin\theta {\rm d}y) \otimes(\cos\theta{\rm d}x+ \sin\theta{\rm d}y) + {\rm d}\theta \otimes{\rm d}\theta)$ and with spatial arc-length parametrization *s* in the plane $\mathbb{R}^{2}$. Surprisingly, *s*-parametrization simplifies the exponential map, the curvature formulas, the cusp-surface, and the boundary value problem,

## Introduction

Curve optimization plays a major role both in imaging and visual perception. In imaging there exist many works on snakes and active contour modeling, whereas in visual perception illusionary contours arise in various optical illusions [48, 52]. Mostly, these optimal curve models rely on Euler’s elastica curves [33] (minimizing $\int(\kappa ^{2}+ \xi^{2}) {\rm d}s$) to obtain extensions where typically external forces to the data are included, cf. [5, 18, 21, 60, 61].

**Fig. 1:**
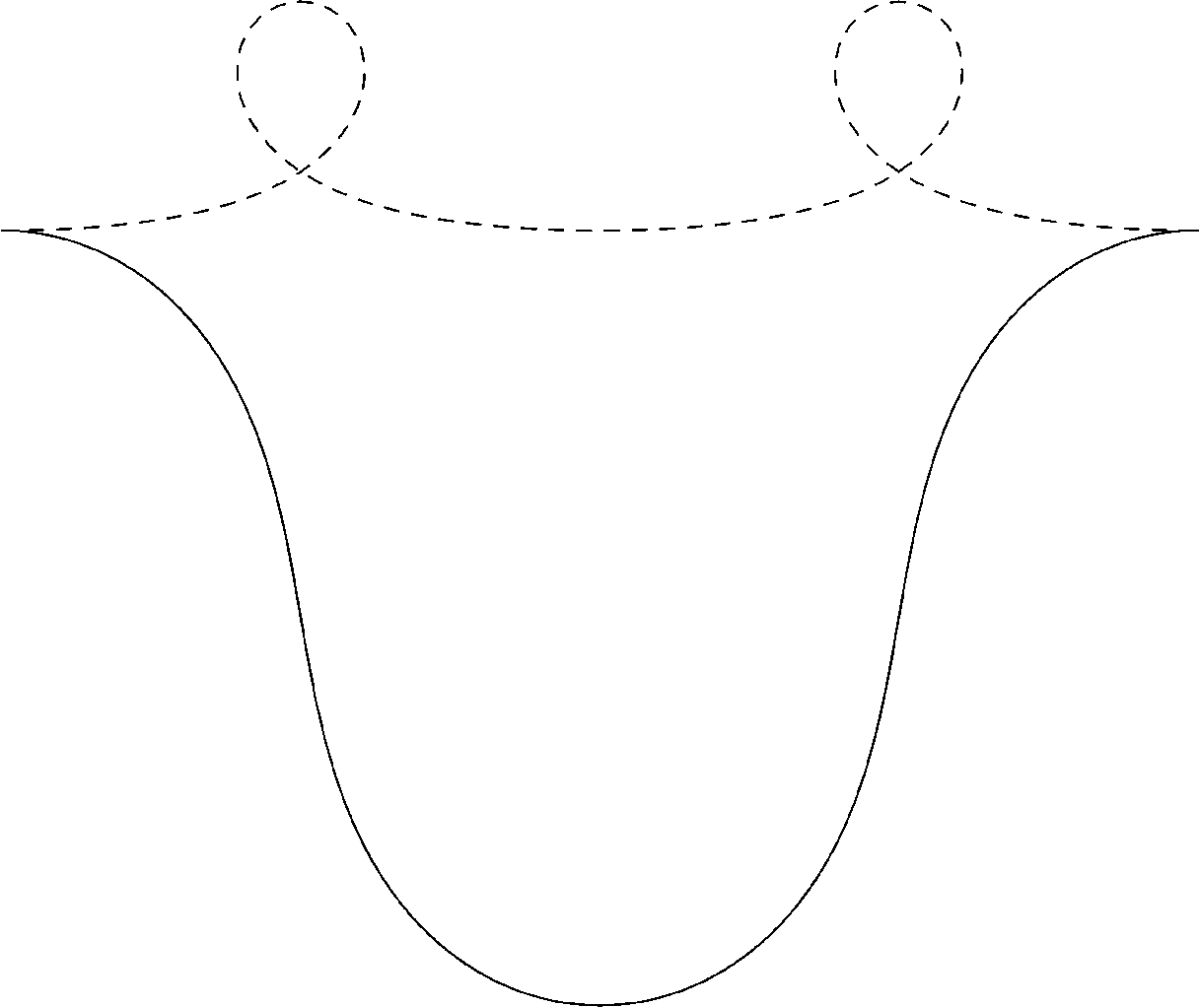
Stationary curves of the elastica problem ($\int_{0}^{\ell} \kappa^{2}(s)+\xi^{2} {\rm d} s \rightarrow\mathrm{min}$) do not need to be global minimizers, cf. [54, 66]. E.g. the non-dashed elastica is a global minimum (for *ξ*=1), whereas in *dashed lines* we have depicted a local minimum connecting the same boundary conditions

The elastica problem suffers from the well-known fact that not every stationary curve is a global minimizer, e.g. many local minimizers exist, cf. Fig. 1. Stationarity of a curve can be reasonably checked by the visual system using local perturbations, whereas checking for (global) optimality [54, 66] is much more difficult. Some visual illusions (e.g. the Kanisza triangle) involve corners requiring abrupt resetting of initial and ending conditions, which are difficult to explain in the elastica model. Another problem with elastica is that it is very hard to solve the boundary value problem analytically [4, 6] (due to a highly non-linear ODE for curvature [48]) and this requires efficient numerical 3D shooting schemes.

On top of that elastica curves relate to modes of the direction process (for contour-completion [24]) where the direction of an oriented random walker is deterministic and its orientation is random. Such deterministic propagation only makes sense when the initial orientation is sharply defined. Instead Brownian motion with random behavior both in spatial propagation direction and in orientation direction [1, 22, 25], relates to hypo-elliptic diffusion on the planar roto-translation group. Such a Brownian motion models contour enhancement [25] rather than contour completion [24], see [28] for a short overview. The corresponding Brownian bridge measures [27, 67] (relating to so-called completion fields in imaging [4, 24, 63, 64]) tend to concentrate towards optimal sub-Riemannian geodesics [12, 15, 22, 26, 47, 56]. So both elastica curves and sub-Riemannian geodesics relate to two different fundamental left-invariant stochastic processes [28] on sub-Riemannian manifolds on the 2D-Euclidean motion group SE(2), (respectively to the direction process [24, 48] and to hypo-elliptic Brownian motion [1, 22, 25]).

In short, advantages of the sub-Riemannian geodesic model over the elastica model are:

- Every cuspless sub-Riemannian geodesic (stationary curve) is a global minimizer [15, 16].
- The Euler-Lagrange ODE for normalized curvature $z=\kappa/\sqrt {\kappa^{2}+\xi^{2}}$ can be reduced to a linear one.
- The boundary value problem can be tackled via effective analytic techniques.
- The locations where global optimality is lost can be derived explicitly.
- Sub-Riemannian geodesics are parametrization independent in the roto-translation group SE(2), which is encoded via a pinwheel structure of cortical columns in the primary visual cortex [50, 51].

**Fig. 2:**
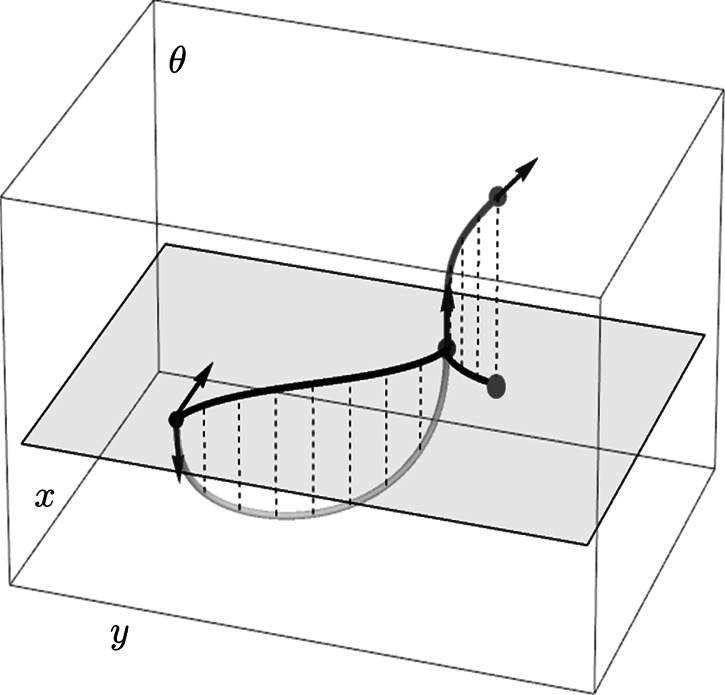
An example of a smooth sub-Riemannian geodesic *γ*=(*x*(⋅),*y*(⋅),*θ*(⋅)) (in *purple*) in auxiliary problem **P**_MEC_, Eq. (12), whose spatial projection (in *black*) shows a cusp (*red point*). A cusp point is a point (*x*,*y*,*θ*) on *γ* such that the velocity (*black arrow*) $\dot{\mathbf{x}}$ of the projected curve **x**(⋅)=(*x*(⋅),*y*(⋅)) switches sign at (*x*,*y*). At such a point in $\mathrm{SE}(2)\equiv\mathbb{R}^{2}\rtimes S^{1}$ the tangent vector points (*blue arrow*) in *θ*-direction (Color figure online)

**Fig. 3:**
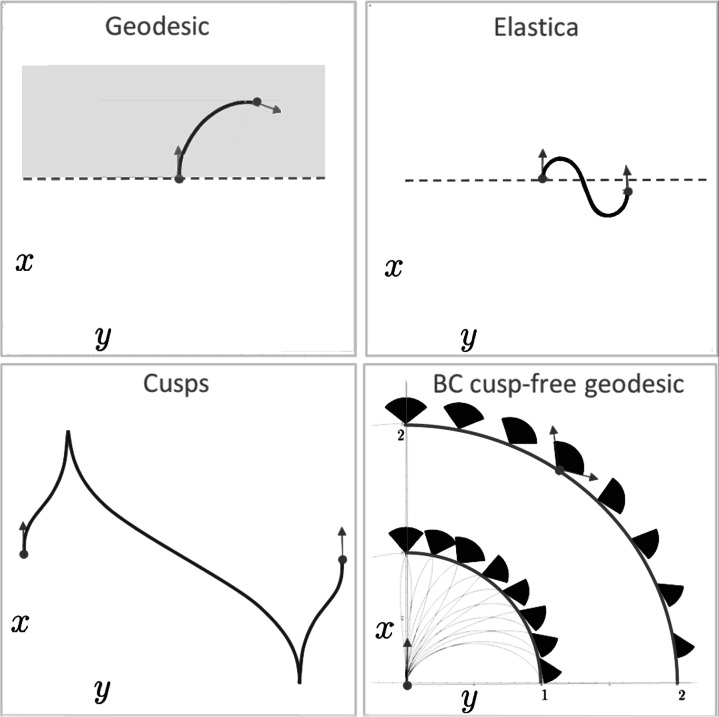
*Top left*: example of a spatially projected sub-Riemannian geodesic without cusp (i.e. a solution of **P**_curve_). *Top right*: example of an elastica curve reaching points *x*<0. Such a (weak) connection is not possible with sub-Riemannian geodesics. Instead we see in the *bottom left figure* a comparable example of a spatially projected sub-Riemannian geodesic connecting the *g*_*in*_=(0,0,0) with *g*_*fin*_=(0,*y*_*fin*_,0) via two cusps. *Bottom right*: not all points in *x*≥0 can be reached via a globally minimizing geodesic, here we have depicted the set $\mathcal{R}$ of admissible end-conditions *g*_*fin*_=(*x*_*fin*_,*y*_*fin*_,*θ*_*fin*_) via black cones on half circles with radius 1 and 2

However, the practical drawback of sub-Riemannian geodesics compared to elastica is that their spatial projections may exhibit cusps and it is hard to analyze when such a cusp occurs. See Fig. 2. Therefore, in this article we provide a complete analysis of such sub-Riemannian geodesics, their parametrization, solving the boundary value problem, and we show precisely when a cusp occurs. See Fig. 3.

A variant of the sub-Riemannian problem that ensures avoiding cusps is the following variational problem, here formulated on the plane:

**P**: Fix *ξ*>0 and boundary conditions $g_{in}=(x_{in},y_{in},\theta _{in}), g_{fin}=(x_{fin},y_{fin},\theta_{fin})\in\mathbb{R}^{2}\times S^{1}$. On the space of (regular enough) planar curves, parameterized by planar arclength *s*>0, we aim to find the solutions of:
  1 $$\begin{aligned} &\mathbf{x}(0)=(x_{in},y_{in}),\quad\quad\mathbf{x}(\ell )=(x_{fin},y_{fin}) , \\ &\dot{\mathbf{x}}(0)=(\cos(\theta_{in}), \sin(\theta_{in})) , \end{aligned}$$
  2 $$\begin{aligned} &\dot{\mathbf{x}}(\ell)=(\cos(\theta_{fin}), \sin(\theta _{fin})), \\ & \int_0^\ell\sqrt{\xi^2+(\kappa (s))^2}~ds\to\min~~(\mbox{with $\ell$ free}). \end{aligned}$$

Here $\kappa(s)=\frac{\dot{x}(s) \ddot{y}(s)-\dot{y}(s)\ddot{x}(s)}{(|\dot{x}(s)|^{2}+|\dot{y}(s)|^{2})^{3/2}}$ is the geodesic curvature of the planar curve **x**(⋅)=(*x*(⋅),*y*(⋅))^*T*^.

This variational problem was studied as a possible model of the mechanism used by the visual cortex V1 to reconstruct curves which are partially hidden or corrupted. This model was initially due to Petitot (see [50, 51] and references therein). Subsequently, the sub-Riemannian structure was introduced in the problem by Petitot [52] for the contact geometry of the fiber bundle of the 1-jets of curves in the plane (the polarized Heisenberg group), whereas Citti and Sarti [22] introduced the sub-Riemannian structure in SE(2) in problem **P**. The group of planar rotations and translations SE(2) is the true symmetry group underlying problem **P**. Therefore, we build on the SE(2) sub-Riemannian viewpoint first proposed by Citti and Sarti [22], and we solve their cortical model for all appropriate end-conditions. The stationary curves of problem **P** were derived by the authors of this paper in [12, 26]. The problem was also studied by Hladky and Pauls in [40], and by Ben-Yosef and Ben-Shahar in [11].

In this article we will show that the model coincides^1^ with the circle bundle model by Petitot [52] and that its minimizers correspond to spatial projections of *cuspless* sub-Riemannian geodesics within $\mathbb {R}^{2}\rtimes S^{1}$.

### Remark 1.1

Problem **P** is well-posed if and only if,^2^

3 $$ \begin{array}{c} \left( R_{\theta_{in}}^{-1} \left( \begin{array}{c} x_{fin} - x_{in} \\ y_{fin} - y_{in} \end{array} \right) , \theta_{fin} - \theta_{in} \right) {\large\in\mathcal{R}}, \end{array} $$

where $R_{\theta_{in}}$ denotes the counterclockwise rotation over *θ*_*in*_ in the spatial plane and where $\mathcal{R}$ is a particular subset $\mathbb{R}^{2} \times S^{1}$ (equal to the range of the underlying exponential map of **P**_curve_ which we will define and derive later in this article), cf. [15, 16].

We will see in the following that this set $\mathcal{R}$ is the set of all endpoints in $\mathbb{R}^{2} \times S^{1}$ that can be connected with a cuspless stationary curve of problem **P**, starting from (0,0,0).

### Remark 1.2

The physical dimension of parameter *ξ* is [Length]^−1^. From a physical point of view it is crucial to make the energy integrand dimensionally consistent. However, the problem with (**x**(0),*θ*(0))=(0,0,0) and *ξ*>0 is equivalent up to a scaling to the problem with *ξ*=1: The minimizer **x** of **P** with *ξ*>0 and boundary conditions (**0**,0) and (**x**_1_,*θ*_1_) relates to the minimizer $\overline{\mathbf{x}}$ of **P** with *ξ*=1 and boundary conditions (**0**,0) and (*ξ* **x**_1_,*θ*_1_), by spatial re-scaling: $\mathbf{x}(s)=\xi^{-1} \overline{\mathbf{x}}(s)$. Therefore, in the remainder of this article we just consider the case *ξ*=1 for simplicity.

It is not straightforward to derive the exact Euler-Lagrange equations together with a necessary geometric study of the set of all possible solution curves. The exact solutions to the problem can be derived using 3 types of techniques:

1. Direct derivation of the Euler-Lagrange equation. E.g. the approach by Mumford [48], yielding a direct approach to the ODE for the curvature, see Appendix A.
2. The Pontryagin Maximum principle: A geometrical control theory approach based on *Hamiltonians*, cf. [3, 12, 47, 53] and Appendix D.
3. The Bryant and Griffith’s approach (based on the works by Marsen-Weinstein on reduction in theoretical mechanics [44]) using a symplectic differential geometrical approach based on *Lagrangians* [26, App. A], cf. [19].

In this article we will apply all three techniques as they are complementary. Furthermore, we aim to provide a complete overview on the surprisingly tedious problem (many inaccurate and/or incomplete results on the stationary curves have appeared in the mathematical imaging literature). Finally, we want to connect remarkably different approaches in previous works [11, 14, 22, 26, 47, 58] on the topic.

The first approach very efficiently produces only the Euler-Lagrange equation for the curvature of stationary curves, but lacks integration of a single curve and lacks a geometric study of the continuum of all stationary curves that arise by varying the possible boundary conditions.

The second approach includes profound geometrical understanding from a *Hamiltonian* point of view and deals with local optimality [3] of stationary curves.

The third approach^3^ takes a *Lagrangian* point of view and provides additional differential geometrical tools from theoretical mechanics that help integrating and structuring the canonical equations. These additional techniques will be of use in deriving semi-analytic solutions to the boundary value problem and in the modeling of association fields.

All three approaches provide, among other results, the following linear hyperbolic ODE

4 $$\begin{aligned} &\ddot{z}(s)= \xi^{2} z(s)\quad \textrm{with }z(s) \in(-1,1) \\ &\quad\Leftrightarrow\quad \frac{d}{ds} \left( \begin{array}{c} z \\ \dot{z} \end{array} \right) = \left( \begin{array}{c@{\quad}c} 0 & 1 \\ \xi^2 & 0 \end{array} \right) \left( \begin{array}{c} z \\ \dot{z} \end{array} \right) \end{aligned}$$

for normalized curvature

5 $$ z(s)= \frac{\kappa(s)}{\sqrt{\kappa^{2}(s)+\xi^2}}= \frac{d\theta }{dt}\bigl(t(s)\bigr), $$

where *s* denotes spatial arc-length and *κ*(*s*) denotes curvature of the spatial part *r*↦**x**(*r*) of a geodesic $\gamma=(\mathbf{x},\theta):[0,\ell] \to\mathbb{R}^{2}\rtimes S^{1}$, with $\theta(s)=\operatorname{arg}(\dot{x}(s)+i\dot{y}(s))$. Such geodesics are globally minimizing, cf. [15, 16] and Theorem 1 below). Furthermore,

6 $$ t(s)=\int_{0}^{s}\sqrt{|\kappa( \tau)|^2 +\xi^{2}} \, {\rm d}\tau $$

denotes sub-Riemannian arclength *t* as a function of *s* along a sub-Riemannian geodesic. Recall that spatial arclength *s* and sub-Riemannian arclength *t* are respectively determined by

7 $$\begin{aligned} \begin{aligned} &|\dot{x}(s)|^2 +|\dot{y}(s)|^2=1, \\ &\xi^{2}|\dot{x}(t)|^2 + \xi^{2}|\dot{y}(t)|^2 + |\dot{\theta}(t)|^2=1. \end{aligned} \end{aligned}$$

As a particular case of Eq. (6), the total sub-Riemannian arc-length *T* of the lifted curve *s*↦*γ*=(**x**(*s*),*θ*(*s*)) with $\theta(s)=\arg(\dot{x}(x)+i \, \dot {y}(s))$, relates to the total length *ℓ* of the spatial curve *s*↦**x**(*s*) via *T*=*t*(*ℓ*).

Firstly, application of Mumford’s approach for deriving the ODE for curvature of elastica, to problem **P** is relatively straightforward, see Appendix A, but does not explicitly involve geometrical control and the Frenet formula still needs to be integrated.

Secondly, in our previous work [16] we considered an extended mechanical problem **P**_MEC_ related to **P**. This problem **P**_MEC_ will soon be explained in detail in Sect. 1.1, and is completely solved by Sachkov et al. in [47, 55, 56]. Application of the Pontryagin maximum principle to this related problem **P**_MEC_ (after squaring the Lagrangian and constraining the total time to a fixed^4^ *T*) yields for *ξ*=1 the maximized Hamiltonian^5^

8 $$ H(p)= \frac{1}{2} \bigl((p_{2} \cos \theta+p_{3} \sin\theta)^{2} +p_{1}^{2} \bigr) $$

with momentum $p=p_{1} {\rm d}\theta+p_{2}{\rm d}x +p_{3} {\rm d}y $ and the induced canonical equations

$$\begin{aligned} &\frac{d\theta}{dt}= \frac{\partial H}{\partial p_{1}},\quad\quad \frac{dx}{dt}= \frac{\partial H}{\partial p_{2}},\quad\quad \frac{dy}{dt}= \frac{\partial H}{\partial p_{3}}, \\ &\dot{p}_{1}= -\frac{\partial H}{\partial\theta},\quad\quad \dot{p}_{2}= -\frac{\partial H}{\partial x}=0,\quad\quad \dot{p}_{3}= -\frac{\partial H}{\partial y}=0, \end{aligned}$$

which via re-parametrization of cylinder $H(p)=\frac{1}{2}$

9 $$\begin{aligned} \begin{aligned} &\sin(\nu/2)= p_{2} \cos( \theta) + p_{3} \sin( \theta), \\ &\cos(\nu/2)= -p_{1}, \\ &c= 2 (p_3 \cos( \theta) - p_{2} \sin( \theta )), \end{aligned} \end{aligned}$$

produces the mathematical pendulum ODE

10 $$ \begin{aligned} &\ddot{\nu}(t)=-\sin\nu(t), \quad\textrm{with } \nu(t) \in(-\pi ,3\pi)\\ &\quad\Leftrightarrow\quad \frac{d}{dt} \left( \begin{array}{c} \nu\\ c \end{array} \right) = \left( \begin{array}{c} c \\ -\sin\nu \end{array} \right),\quad \textrm{with }c:=\dot{\nu}. \end{aligned} $$

For details on the involved computation see [16, 47].

Thirdly, application of the Bryant and Griffith’s (Lagrangian) approach to problem **P** will yield a canonical Pfaffian system on an extended manifold whose elements involve both position, orientation, control (curvature and length), spatial momentum and angular momentum. We will show that the essential part of this Pfaffian system is equivalent to $\nabla_{\dot{\gamma}} p = 0$ where ∇ denotes a Cartan connection and *p* denotes momentum as a co-vector within $T^{*}(\mathbb {R}^{2}\rtimes S^{1})$. This fundamental identity allows us to analytically solve the boundary value problem.

### Lift problem $\bf{P}$ to the roto-translation group

Problem $\bf{P}$ relates to two different geometric control problems (**P**_curve_ and **P**_MEC_):

-
  **P**_curve_: Fix *ξ*>0 and boundary conditions $(x_{in},y_{in},\theta _{in}), (x_{fin},y_{fin},\theta_{fin})\in\mathbb{R}^{2}\times S^{1}$, with (*x*_*in*_,*y*_*in*_)≠(*x*_*fin*_,*y*_*fin*_). In the space of integrable (possibly non-smooth) controls $v(\cdot ):[0,\ell]\to\mathbb{R}$, we aim to solve:
    11 $$\begin{aligned} &(x(0),y(0),\theta(0))=(x_{in},y_{in},\theta_{in}), \\ &(x(\ell ),y(\ell),\theta(\ell))=(x_{fin},y_{fin},\theta_{fin}), \\ & \left( \begin{array}{c} \frac{dx}{ds}(s)\\ \frac{dy}{ds}(s)\\ \frac{d\theta }{ds}(s) \end{array} \right)=\left( \begin{array}{c} \cos(\theta(s)) \\ \sin(\theta(s)) \\ 0 \end{array} \right)+v(s) \left( \begin{array}{c} 0\\ 0\\ 1 \end{array} \right), \\ & \int_0^\ell\sqrt{\xi^2 + \kappa(s)^2}~{\rm d}s= \int_0^\ell\sqrt{\xi^2 + v(s)^2}{\rm d}s\\ &\quad\to\min\quad (\mbox{here } \ell\geq0 \mbox{ is free}) \end{aligned}$$
  Since in this problem we are taking *v*(⋅)∈*L* ^1^([0,*ℓ*]), the curve $\gamma=(x(\cdot),y(\cdot),\theta(\cdot)):[0,\ell]\to \mathbb{R} ^{2}\times S^{1}$ is absolutely continuous and curve $\mathbf{x}=(x(\cdot ),y(\cdot)):[0,\ell]\to\mathbb{R}^{2}$ is in Sobolev space $W^{2,1}([0,\ell],\mathbb{R}^{2})$.
-
  **P**_MEC_: Fix *ξ*>0 and boundary conditions $(x_{in},y_{in},\theta _{in}), (x_{fin},y_{fin},\theta_{fin})\in\mathbb{R}^{2}\times S^{1}$. In the space of *L* ^∞^ controls $\tilde{u}(\cdot),\tilde {v}(\cdot):[0,\ell]\to\mathbb{R}$, solve:
    12 $$\begin{aligned} &(x(0),y(0),\theta(0))=(x_{in},y_{in},\theta_{in}), \\ &(x(T),y(T),\theta(T))=(x_{fin},y_{fin},\theta_{fin}) , \\ & \left( \begin{array}{c} \frac{dx}{dt}(t)\\ \frac{dy}{dt}(t)\\ \frac{d\theta }{dt}(t) \end{array} \right)=\tilde{u}(t) \left( \begin{array}{c} \cos(\theta(t)) \\ \sin(\theta(t)) \\ 0 \end{array} \right)+\tilde{v}(t) \left( \begin{array}{c} 0\\ 0\\ 1 \end{array} \right) \\ & \int_0^T\sqrt{\xi^2\tilde {u}(t)^2+\tilde{v}(t)^2}~{\rm d}t \\ &\quad \to\min\quad (\mbox{here } T\geq0 \mbox{ is free}) \end{aligned}$$
  Problem **P**_MEC_ has a solution by Chow’s and Fillipov’s theorems [3] regardless the choice of end-condition and has been completely solved in a series of papers by one of the authors (see [47, 55, 56]). It gives rise to a sub-Riemannian distance on the sub-Riemannian manifold within SE(2) as we will explain next.

The space $\mathbb{R}^{2}\times S^{1}$ can be equipped with a natural group product

13 $$ (\mathbf{x},\theta) \cdot\bigl(\mathbf{x}', \theta'\bigr)= \bigl(R_{\theta} \mathbf{x}'+\mathbf{x}, \theta+\theta'\bigr) $$

where *R*_*θ*_ denotes a counter-clockwise rotation over angle *θ*∈(−*π*,*π*] and with **x**=(*x*,*y*)^*T*^ and **x**′=(*x*′,*y*′)^*T*^ so that it becomes isomorphic to the 2D (special) Euclidean motion group consisting of rotations and translations in the plane, also known as roto-translation group, and commonly denoted by SE(2). As SE(2) acts transitive and free on the set of positions and orientations $\mathbb{R}^{2}\times S^{1}$ we can identify point on orbits (*x*,*y*,*θ*) starting from the unity (0,0,0) with the corresponding group elements (*x*,*y*,*R*_*θ*_). Therefore we write $\mathbb{R}^{2}\rtimes S^{1} \equiv\mathrm{SE}(2)$ to stress that the set $\mathbb{R}^{2}\times S^{1}$ is equipped with a (semi-direct) group product (13). Now both problems **P**_curve_ and **P**_MEC_ are invariant with respect to rotations and translations so we may as well set (*x*_*in*_,*y*_*in*_,*θ*_*in*_)=(0,0,0). Indeed, given a problem with general boundary conditions (*x*_*in*_,*y*_*in*_,*θ*_*in*_) and (*x*_*fin*_,*y*_*fin*_,*θ*_*fin*_), its minimizer *γ*_*opt*_ (when it exists) is $(x_{in},y_{in},\theta_{in}) \cdot\tilde{\gamma}_{opt}$, where $\tilde{\gamma}_{opt}$ is the minimizer from (0,0,0) to

$$(x_{in},y_{in},\theta_{in})^{-1} \cdot(x_{fin},y_{fin},\theta_{fin}). $$

Throughout this article we use the following notation for the moving frame $\{\mathcal{A}_{1},\mathcal{A}_{2},\mathcal{A}_{3}\}$ of left-invariant vector fields

14 $$ \begin{aligned} &X_{1}=(0,0,1)^{T} \leftrightarrow\mathcal{A}_{1}:= \partial _{\theta}, \\ &X_{2}=(\cos\theta,\sin\theta,0)^{T} \leftrightarrow\mathcal {A}_{2}:= \cos\theta\partial_{x} +\sin\theta\partial_{y}, \\ &X_{3}=(-\sin\theta, \cos\theta,0)^{T} \leftrightarrow \mathcal {A}_{3}:=-\sin\theta\partial_{x}+ \cos\theta\partial_{y}, \end{aligned} $$

where on the right we consider vector fields as differential operators, for details on such identification see e.g. [3, 7]. The corresponding co-frame of left-invariant dual basis vectors will be denoted by

15 $$ \begin{aligned} &\hat{X}^{1}=(0,0,1) \leftrightarrow\omega^{1}:= {\rm d}\theta, \\ &\hat{X}^{2}=(\cos\theta,\sin\theta,0) \leftrightarrow\omega ^{2}:= \cos\theta\,{\rm d}x + \sin\theta\,{\rm d}y, \\ &\hat{X}^{3}=(-\sin\theta,\cos\theta,0) \leftrightarrow\omega ^{3}:= -\sin\theta\,{\rm d}x + \cos\theta\,{\rm d}y, \end{aligned} $$

where frame and dual frame relate via

$$\hat{X}^{i} \cdot X_{j}= \bigl\langle \omega^{i}, \mathcal{A}_{j} \bigr\rangle =\delta^{i}_{j}, \quad i,j=1,2,3, $$

where in the righthand side we have the Kronecker symbols $\delta ^{i}_{j}=1$ if *i*=*j* and 0 else. Problem **P**_MEC_ can now be reformulated as the computation of

16 $$\begin{aligned} &d(g_{in}, g_{fin}) \\ &\quad= \inf_{\footnotesize \begin{array}{c} \gamma\in\mathrm{Lip}([0,T],\mathrm{SE}(2)), T>0 \\ \gamma(0)=g_{in}, \gamma(T)=g_{fin} \\ \langle\omega^{3}, \dot{\gamma}\rangle=0 \end{array} } \int_{0}^{T} \sqrt{\mathcal{G}_{\xi}(\dot{\gamma}(t),\dot{\gamma }(t))} {\rm d}t \end{aligned}$$

where *d* denotes the sub-Riemannian distance^6^ on the sub-Riemannian manifold

17 $$ \bigl(\mathrm{SE}(2), \Delta:=\mathrm {Ker}\bigl(\omega^{3} \bigr)=\mathrm{span}\{\mathcal{A}_{1},\mathcal{A}_{2}\}, \mathcal{G}_{\xi}\bigr), $$

with sub-Riemannian metric tensor

18 $$ \mathcal{G}_{\xi}= \omega^{1}\otimes \omega^{1}+\xi^2 \omega^{2} \otimes \omega^{2}. $$

#### Remark 1.3

The sub-Riemannian structure is 3D contact and analytic and therefore we have non-existence of abnormal extrema and all minimizers are analytic, where we note that distribution Δ is 2-generating cf.[3, Chap. 20.5.1].

Problem **P**_MEC_ is to be considered as an *auxiliary* mechanical problem (of optimal path planning of a moving car carrying a steering wheel and the ability to drive both forwardly and backwardly) associated to **P**_curve_. To this end we stress that **P**_MEC_ cannot be interpreted as a problem of reconstruction of planar curves, [14]. The problem is that the minimizing curve *γ*=(**x**,*θ*):[0,*T*]→SE(2) may have a vertical tangent vector (i.e. in *θ*-direction) in between the ending conditions, which causes a cusp in the corresponding projected curve *t*↦**x**(*t*) in the plane, see Fig. 2. Such a cusp corresponds to a point on an optimal path where the car is suddenly set in reverse gear.

Problem **P**_MEC_ is invariant under monotonic re-parameterizations and at a cusp spatial arc-length parametrization breaks down. If $(x_{fin},y_{fin},\theta_{fin}) \in\mathcal{R}$ no such cusps arise and **P**_MEC_ and **P**_curve_ are equivalent [15, 16] and we can use arclength parametrization also in **P**_MEC_ (in which case the first control-variable is set to 1, since $\langle\omega^{2}\vert_{\gamma (s)},\dot{\gamma}(s)\rangle=1$). In [16] we have proven the following Theorem.

#### Definition 1

Let $\mathcal{R} \subset\mathrm{SE}(2)$ denote the set of end-points in SE(2) that can be reached from *e* with a stationary curve of problem **P**_curve_.

#### Theorem 1

*In* **P**_curve_ *we set initial condition* (*x*_*in*_,*y*_*in*_,*θ*_*in*_)=*e*=(0,0,0) *and consider* $(x_{fin},y_{fin},\theta_{fin}) \in \mathbb{R} ^{2} \rtimes S^{1}$. *Then*

- $(x_{fin},y_{fin},\theta_{fin}) \in\mathcal{R}$ *if and only if* **P**_curve_ *has a unique minimizing geodesic which exactly coincides with the unique minimizer of* **P**_MEC_.
- $(x_{fin},y_{fin},\theta_{fin}) \notin\mathcal{R}$ *if and only if problem* **P**_curve_ *is ill*-*defined* (*i*.*e*. **P**_curve_ *does not have a minimizer*).^7^

**Fig. 4:**
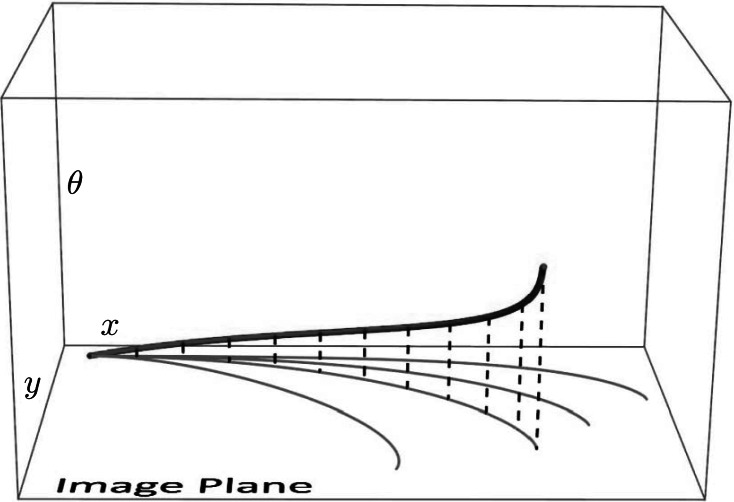
Cuspless sub-Riemannian geodesics (projected on the plane) for admissible boundary conditions modeling the association field as in Fig. 8. According to Theorem 1 they are global minimizers. Remarkably the tangent vector to these geodesics (e.g. the *red* geodesic) is nearly vertical at the end condition and the large curvature at the end condition at the association field, indicate the association field lines end at close vicinity of cusps (Color figure online)

As a result, for the case *g*_*in*_=(0,0,0), we say *g*_*fin*_∈SE(2) is an *admissible* end-condition for **P**_curve_ if $g_{fin} \in\mathcal{R}$, as only for such end-conditions we have existence of a (smooth) global minimizer, see also [12]. See Fig. 4.

## Structure of the Article

Firstly, in Sect. 3 we consider the origin of the problem of finding cuspless sub-Riemannian geodesics in $(\mathrm {SE}(2),\Delta , \mathcal{G}_{\beta})$, which includes cortical modeling of the primary visual cortex and association fields.

In Sect. 4 we provide a short road map on how to connect two natural parameterizations. The *cuspless* sub-Riemanian geodesics in the sub-Riemannian manifold $(\mathrm{SE}(2),\Delta,\mathcal{G}_{\beta})$ can be properly parameterized by the sub-Riemannian arclength parametrization (via *t*) or by spatial arclength parametrization (via *s*). Parametrization via *t* yields the central part of the mathematical pendulum phase portrait (recall Eq. (10)), whereas parametrization via *s* yields a central part of a hyperbolic phase portrait (recall Eq. (4)). The hyperbolic phase portrait does *not* coincide with a local linearization approximation (as in Hartman-Grobman’s theorem [38]). In fact, it is *globally* equivalent to the relevant part of the pendulum phase portrait (i.e. the part associated to cuspless sub-Riemannian geodesics). The involved coordinate transforms are global diffeomorphisms.

In Sect. 5 we define the exponential map [2, 47] for **P**_curve_ and **P**_MEC_. Then we show that the set $\mathcal{R} \subset\mathrm{SE}(2)$ (consisting of admissible end-conditions) equals the range of the exponential map of **P**_curve_. We will provide novel explicit formulas for the exponential map for **P**_curve_ using spatial arc length parametrization *s* and moreover, for completeness and comparison, in Appendix B we will also provide explicit formulas for the exponential map of **P**_MEC_ that were previously derived in previous work [47] by one of the authors.

We show that the exponential map of **P**_curve_ follows by restriction of **P**_MEC_ to the strip $(\nu,c) \in[0,2\pi] \times\mathbb{R}$, see Fig. 9. A quick comparison in Appendix B learns us that spatial arc-length parametrization (also suggested in [22]) simplifies the formulas of the (globally minimizing, cuspless) geodesics of **P**_curve_ considerably.

As the set of admissible end-conditions $\mathcal{R}$ equals the range of the exponential map of **P**_curve_, we analyze this important set $\mathcal{R}$ carefully in Sect. 6. More precisely, we

1. show that $\mathcal{R}$ is contained in half space *x*≥0 and (0,*y*_fin_)≠(0,0) is reached with angle *π*,
2. show in Theorem 6 that the boundary $\partial \mathcal{R}$ consists of the union of endpoints of minimizers either starting or ending in a cusp and a vertical line $\mathfrak{l}$ above (0,0,0), and we compute the total spatial arc-length towards a cusp,
3. analyze and plot the cones of reachable angles *θ*_fin_ per spatial endpoint (*x*_fin_,*y*_fin_),
4. prove homeomorphic and diffeomorphic properties of the exponential map in Theorem 6,
5. show in Lemma 8 that geodesics that end with a cusp at $\theta_{fin}=\frac{\pi}{2}$ are precisely those with stationary curvature ($\dot{\kappa}(0)=0$) at the origin.

In Sect. 7 we solve the boundary value problem, where we derive a (semi)-analytic description of the inverse of the exponential map and present a novel efficient algorithm to solve the boundary value problem. This algorithm requires numerical shooting only in a small sub-interval of [−1,1], rather than a numerical shooting algorithm in $\mathbb{R}^{2}\times S^{1}$.

In Sect. 8 we show a clear similarity of cuspless sub-Riemannian geodesics and the association field lines from psychophysics [34] and neuro-physiology [52]. This is not surprising as we will show that sub-Riemannian geodesics allowing *x*-parametrization, exactly solve the circle bundle model for association fields by Petitot, cf. [52]. It is remarkable that the endings of association fields are close to the cusp-surface $\partial\mathcal{R}$, which we underpin with Lemma 8 and Remark 8.1.

For a concise overview of previous mathematical models for association fields and their direct relation to the cuspless sub-Riemannian geodesic model proposed in this article we refer to the final subsection in Appendix G.

## Origin of Problem $\bf{P}$: Cortical Modeling

In a simplified model (see [51, p. 79]), neurons of V1 are grouped into *orientation columns*, each of them being sensitive to visual stimuli at a given point of the retina and for a given direction on it. The retina is modeled by the real plane.

**Fig. 5:**
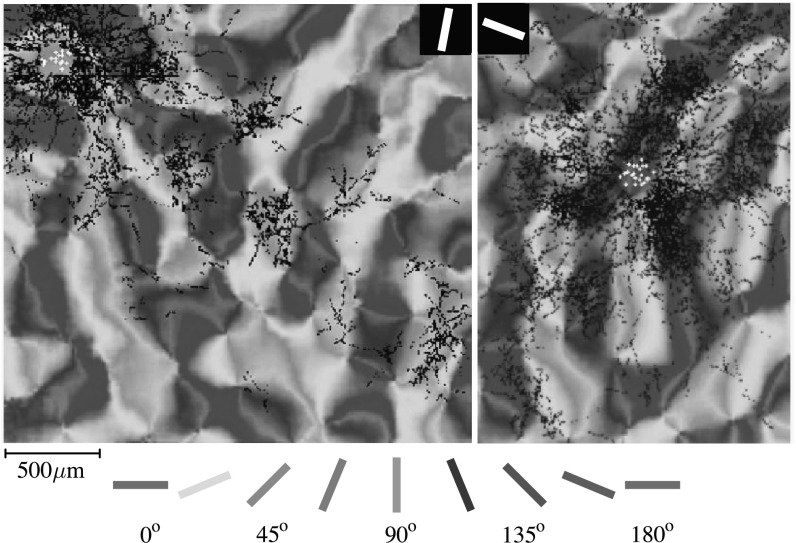
Receptive fields in the visual cortex of many mammalians are tuned to various locations and orientations. Assemblies of oriented receptive fields are grouped together on the surface of the primary visual cortex in a pinwheel like structure. Orientation sensitivity in the primary visual cortex of a tree shrew, replicated from [17], © 1997 Society of Neuroscience. *Black dots* indicate horizontal connections to aligned neurons with an 80^∘^ orientation preference shown by the *white dots*. The *figure* on the *right* indicates horizontal connections at 160^∘^

**Fig. 6:**
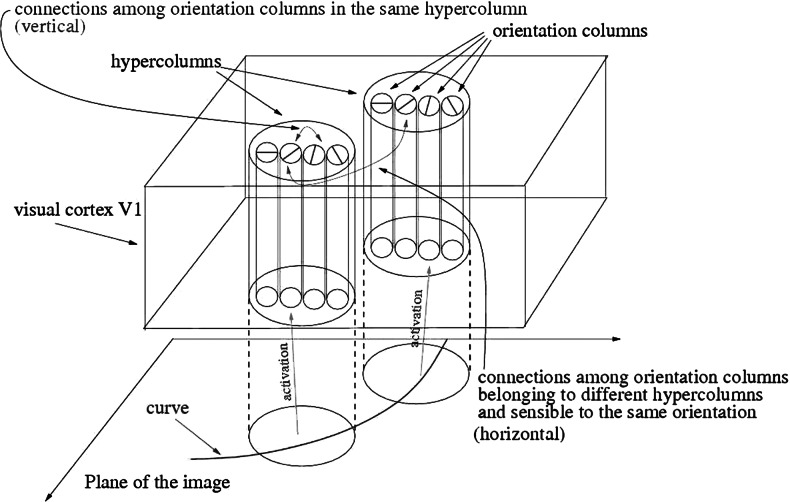
A scheme of the primary visual cortex V1

Orientation columns are connected between them in two different ways. The first kind is given by *vertical connections*, which connect orientation columns belonging to the same hypercolumn and sensible to similar directions. The second is given by the *horizontal connections* across the orientation columns which checks for alignment of local orientations. See Figs. 5 and 6.

**Fig. 7:**
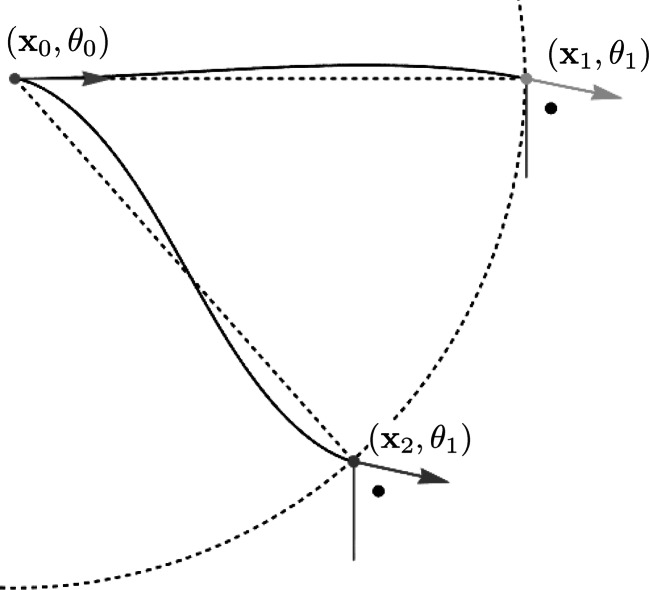
Positions and orientations are coupled. The spatial and angular distance between (**x**_1_,*θ*_1_) and (**x**_0_,*θ*_0_) is the same as the spatial and angular distance of (**x**_2_,*θ*_1_) between (**x**_0_,*θ*_0_). However, (**x**_1_,*θ*_1_) is much more aligned with (**x**_0_,*θ*_0_) than (**x**_2_,*θ*_1_) is. The left-invariant sub-Riemannian structure on the space $\mathbb{R}^{2} \rtimes S^{1}$ takes this alignment into account. The connecting curves are spatial projections of sub-Riemannian geodesics in SE(2) for $\xi=\frac{1}{2}$ (with $\mathbf {x}_{0}=(0,0), \mathbf{x}_{1}=(5,0), \mathbf{x}_{2}=(4,3), \theta_{0}=0, \theta _{1}=-\frac {\pi}{5}$)

The human visual system not only performs a score of local orientations (organized by a pinwheel structure in V1). It also checks (a priori) for alignment of local orientations in the enhancement and detection of elongated structures. In modeling both procedures it is crucial that one does not consider $\mathbb{R}^{2}\times S^{1}$ as a flat Cartesian space. See Fig. 7.

The Euclidean motion group acts transitively and free on the space of positions and orientations, allowing us to identify the coupled space of positions and orientations $\mathbb{R}^{2}\rtimes S^{1}$ with the roto-translation group $\mathrm{SE}(2)=\mathbb{R}^{2} \rtimes SO(2)$. This imposes a natural Cartan connection [26, 52] on the tangent bundle $T(\mathbb{R}^{2}\rtimes S^{1})$ induced by the push-forward of the left-multiplication of SE(2) onto itself.

**Fig. 8:**
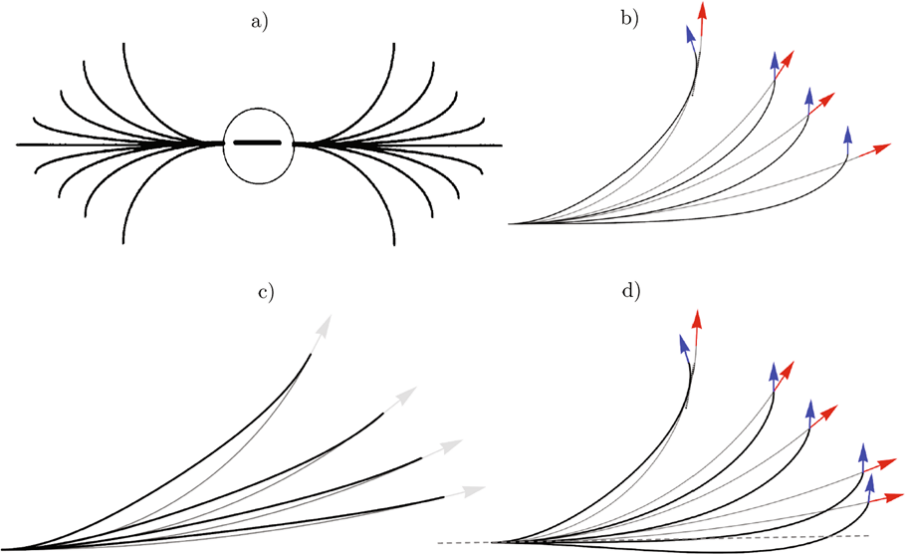
Modeling the association field with sub-Riemannian geodesics and exponential curves, (**a**) the association field [34, 52]. Compare the *upper-right part* of the association field to the following lines: in (**b**) we impose the end condition (*blue arrows*) for the SR-geodesic model in *black* and the end condition (*red arrows*) for the horizontal exponential curve model [57], Eq. (72), in *grey*; (**c**) comparison of sub-Riemannian geodesics with exponential curves with the same (co-circularity) ending conditions; (**d**) as in (**b**) including other ending conditions (Color figure online)

Besides the non-commutative group structure on $\mathbb{R}^{2}\rtimes S^{1}\equiv\mathrm{SE}(2)$, contact geometry plays a major role in the functional architecture of the primary visual cortex (V1) [41], and more precisely its pinwheel structure, cf. [52]. In his paper [52] Petitot shows that the horizontal cortico-cortical connections of V1 implement the contact structure of a continuous fibration *π*:*R*×*P* ^1^→*P* ^1^ with base space the space of the retina and *P* ^1^ the projective line of orientations in the plane. He applies his model to the Field’s, Hayes’ and Hess’ physical concept of an association field, to several models of visual hallucinations [32] and to a variational model of curved modal illusory contours [42, 48, 65]. Such *association fields* reflects the propagation of local orientations in the primary visual cortex. For further remarks on the concept of an association field and its mathematical models see Appendix G. Intuitively, the tangents to the field lines of the association field provide expected local orientations, given that a local orientation is observed at the center of the field in Fig. 8). These association fields have been confirmed by Jean Lorenceau et al. [43] via the method of apparent speed of fast sequences where the apparent velocity is overestimated when the successive elements are aligned in the direction of the motion path and underestimated when the motion is orthogonal to the orientation of the elements. They have also been confirmed by electrophysiological methods measuring the velocity of propagation of horizontal activation [37]. There exist several other interesting low-level vision models and psychophysical measurements that have produced similar fields of association and perceptual grouping [39, 49, 68], for an overview see [52, Chaps. 5.5, 5.6]. Remarkably, psychological physics experiments based on multiple Gabor patch-stimuli indicate a thresholding effect in contour recognition, if the slope variation in two subsequent elements (Gabor patches) is too large no alignment is perceived and if the orientations are no longer tangent but transverse to the curve no alignment is perceived, cf. [52].

In this article we will show that sub-Riemannian geodesics closely model the association fields from psychophysics and that the location of cusps seems to provide a reasonable grouping criterium to connect two local orientations (consistent with endings of the association field), see Fig. 8. Next we will show that it does not matter whether one lifts problem **P** (given by Eqs. (1) and (2)) to the projective line bundle or to the group of rotations and translations in the plane.

### No Need for Projective Line Bundles in **P**_curve_

The **P**_MEC_ problem on $(\mathrm{SE}(2)=\mathbb {R}^{2}\rtimes S^{1}, \Delta=\mathrm {Ker}(\omega^{3}), \mathcal{G}_{\xi})$ can as well be formulated on the projective line bundle *P* ^1^ [14, 52] where antipodal points on the sphere *S* ^1^ are identified. See also [13].

In the setting of **P**_curve_, we then can study the problem with initial condition in the set

$$\left\{ (x_{in},y_{in},\theta_{in}),(x_{in},y_{in}, \theta_{in}+\pi ) \right\}, $$

and similarly for the final condition. Nevertheless, the structure of solutions does not change with respect to the solutions of the standard problem **P**_curve_. Indeed such flips are either not allowed or they do not produce new curves:

- Flipping only one of the boundary conditions is not possible as in this article we shall show that if $(x_{fin},y_{fin}, \theta_{fin}) \in \mathcal{R} \Rightarrow(x_{fin},y_{fin}, \theta_{fin}+\pi) \in (\mathbb{R} ^{2}\times S^{1}) \setminus\mathcal{R}$, i.e. when (*x*_*fin*_,*y*_*fin*_,*θ*_*fin*_) is an admissible ending condition then (*x*_*fin*_,*y*_*fin*_,*θ*_*fin*_+*π*) is not admissible.
- If we both flip (i.e. *θ*↦*θ*+*π*) and switch both the initial and ending condition we get the same curve (in opposite direction).

So when insisting on cuspless solution curves in our central problem **P**, lifting problem **P** to the projective bundle $\mathbb{R}^{2} \rtimes P^{1}$ is equivalent to lifting **P** to $\mathrm{SE}(2)\equiv\mathbb {R}^{2}\rtimes S^{1}$. In fact, identification of antipodal points does not make any difference when considering cuspless sub-Riemannian geodesics in $(\mathrm{SE}(2), \Delta,\mathcal{G}_{\xi})$.

Therefore, in this article we will not identify antipodal points and we focus on problem **P**_curve_ and its corresponding admissible boundary conditions (i.e. an explicit description of the set $\mathcal {R}\subset\mathrm{SE}(2)$).

## Parametrization of Curves in **P**_curve_

The natural parametrization for sub-Riemannian geodesics in **P**_MEC_ is the sub-Riemannian arclength parametrization. However, when considering only those sub-Riemannian geodesics in $(\mathrm{SE}(2),\Delta,\mathcal{G}_{\xi})$ without cusps (as in **P**_curve_), i.e. the cuspless sub-Riemannian geodesics, the problem is actually a planar curve problem (as in **P**) and there it is more natural^8^ to use spatial arclength parametrization.

Recall *t* denotes the sub-Riemannian arclength parameter of a (horizontal) curve *γ*(⋅)=(*x*(⋅),*y*(⋅),*θ*(⋅)) in $(\mathrm{SE}(2),\Delta,\mathcal{G}_{\xi})$ and *s* denotes the spatial arclength parameter of $(x(\cdot),y(\cdot ))=P_{\mathbb{R}^{2}} \gamma(\cdot)$, recall Eq. (7). Then along a horizontal curve $\gamma\in(\mathrm{SE}(2),\Delta,\mathcal {G}_{\xi})$ we have $\kappa(s)=\dot{\theta}(s)$ and $\langle\omega^{2}\vert_{\gamma(s)}, \dot{\gamma}(s) \rangle= \|\dot{\mathbf{x}}(s)\|=1$ and thereby we have

$$\begin{aligned} t(s) =& \int_{0}^{s} \sqrt{\mathcal{G}_{\xi}\vert_{\gamma (\tau)}(\dot{\gamma}(\tau),\dot{\gamma}(\tau))}\, {\rm d}\tau = \int_{0}^{s} \sqrt{\kappa^{2}(\tau)+\xi^2}\, {\rm d}\tau. \end{aligned}$$

**Fig. 9:**
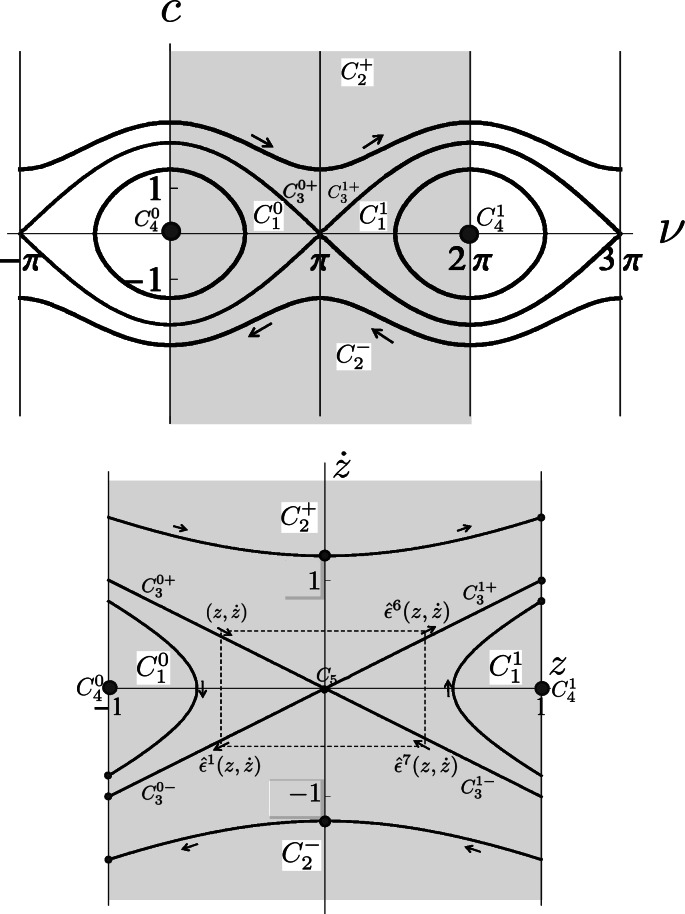
Optimal control via phase portrait (*top*) of the pendulum $(\dot{\nu}(t),\dot {c}(t))=(c(t),-\sin\nu(t))$ using *t*-parametrization and the corresponding (recall Eq. (19)) phase portrait $(\dot{z}(s),\ddot{z}(s))=(\dot{z}(s),\xi^{2} z(s))$ using *s*-parametrization (*bottom*). We have also included the four reflectional symmetries of **P**_curve_, which are half of all reflectional symmetries of **P**_MEC_[47]. The labeling of sub-regions (e.g. $C_{1}^{1}, C_{0}^{1}, C_{2}^{+}, C_{2}^{-}$) follows the conventions in [47]

As mentioned in Remark 1.2, we may as well set *ξ*=1. Furthermore, recall from Eq. (4) that the Euler-Lagrange equation for cuspless sub-Riemannian geodesics in **P**_curve_ is $\ddot{z}(s)=z(s)$, producing a hyperbolic phase portrait where we must restrict ourselves to $z=\kappa/\sqrt{\kappa^{2}+1} \in(-1,1)$. On the other hand, we recall from Eq. (10) the Euler-Lagrange equation for sub-Riemannian geodesics in **P**_MEC_ is given by $\ddot{\nu}(t)=-\sin\nu(t)$ producing a mathematical pendulum phase portrait where we must restrict *ν* to the interior of $\mathbb{R} /(4\pi\mathbb{Z})$ say the open interval (−*π*,3*π*), cf. [47]. The central part *ν*∈(0,2*π*) of the mathematical pendulum relates to the initial momentum components of cuspless sub-Riemannian geodesics. In fact, it is globally equivalent to the hyperbolic phase portrait as follows by the next lemma and Fig. 9.

### Lemma 1

*The central part* (*i*.*e*. *ν*∈(0,2*π*)) *of the mathematical pendulum phase portrait induced by* $\ddot{\nu}(t)=-\sin(\nu(t))$ *is diffeomorphic to a hyperbolic phase portrait of the linear ODE* $\ddot {z}(s)=z(s)$ (*with* |*z*|<1). *The direct coordinate transforms between* (*ν*,*c*) *and* $(z,\dot{z})$ *are given by*

19 $$\begin{aligned} \begin{aligned} &\nu(t) = 2 \arccos(-z(s(t))), \\ &c(t) = 2 \dot{z}(s(t)), \\ &z(s)= -\cos\biggl(\frac{\nu(t(s))}{2} \biggr), \\ &\dot{z}(s)= \frac{1}{2} c(\nu(t(s))), \end{aligned} \end{aligned}$$

*where*

$$\begin{aligned} t(s)=\int_{0}^{s} \sqrt{\kappa^{2}(\tau)+1}\, {\rm d}\tau= \int_{0}^{s}\frac{1}{\sqrt{1- |z(\tau)|^2}}\, {\rm d}\tau. \end{aligned}$$

### Proof

Directly follows by the chain-law:

$$\begin{aligned}& \frac{dc}{dt}= \frac{dc}{d\dot{z}} \frac{d\dot{z}}{ds} \frac {ds}{dt} = 2 z \sqrt{1-z^2} \\& \phantom{\frac{dc}{dt}} = -2 \cos(\nu/2) \sin(\nu/2) = -\sin(\nu), \\& \frac{d\nu}{dt}= \frac{dz}{ds} \frac{ds}{dt} \frac{d\nu}{dz} = \frac{c}{2} \frac{1}{\sqrt{\kappa^{2}+1^2}} \frac{2}{\sqrt {1-z^2}} = c. \end{aligned}$$

Finally, we note that for |*z*(*s*)|<1 the mapping between *s* and *t* is a diffeomorphism. □

## Cusps and the Exponential Map Associated to **P**_curve_ and **P**_MEC_

In order to express the exponential map associated to **P**_curve_(for *ξ*=1) in spatial arclength parametrization we apply Bryant & Griffith’s approach [20], which was previously successfully applied to the elastica problem [19]. Here we will also include an additional viewpoint on this technical approach via the Cartan connection. In case the reader is not so much interested in the geometrical details and underpinnings, it is also possible to skip the following derivations and to continue reading starting from the formulas for the sub-Riemannian geodesics *γ*(*s*) in Theorem 3.

To avoid large and cumbersome computations we first need some preliminaries on moving frames of references and Cartan connections. Recall to this end our notations for left-invariant frame $\{\mathcal {A}_{i}\}_{i=1}^{3}$ given by Eq. (14), and left-invariant co-frame $\{\omega^{i}\}_{i=1}^{3}$ given by Eq. (15). The left-invariant vector fields generate a Lie algebra

$$[\mathcal{A}_{i},\mathcal{A}_{j}]=\sum _{k=1}^{3}c^{k}_{ij} \mathcal{A}_{k}, $$

where the non-zero structure constants are $c^{3}_{12}=-c^{3}_{21}=-c_{13}^{2}=c_{31}^{2}=1$. This Lie-algebra serves as the moving frame of reference in $\mathbb{R} ^{2}\rtimes S^{1} \equiv\mathrm{SE}(2)$. The Cartan connection ∇ on *T*(SE(2)) is given by

$$\begin{aligned}& \nabla_{\dot{\gamma}(s)} \Biggl( \sum_{k=1}^{3} a^{k}\mathcal{A}_{k} \Biggr)\\& \quad := \sum_{k=1}^{3}\dot{a}^{k}(s)\mathcal{A}_{k}\vert_{\gamma (s)} + \sum_{i,j,k=1}^{3} c^{j}_{ki} \dot{\gamma}^{i}(s) a^{k}(s) \mathcal{A}_{j}\vert_{\gamma(s)}. \end{aligned}$$

where we used the following definitions

$$\begin{aligned}& \dot{\gamma}^{i}(s):= \langle\omega^{i}\vert_{\gamma (s)},\dot{\gamma}(s)\rangle, \\& \dot{a}^{k}(s):= \langle{\rm d}a^{k}, \dot{\gamma}(s)\rangle=\sum _{i=1}^{3} \dot{\gamma}^{i}(s) \mathcal {A}_{i}\vert_{\gamma(s)}(a^{k}), \end{aligned}$$

As a result (for details see Eq. (93) and Theorem 12 in Appendix C) covariant differentiation of a momentum covector field

20 $$ p(s)=\sum _{k=1}^{3} \lambda_{k}(s) \omega^{k}\vert_{\gamma(s)} $$

along a curve *γ*:[0,*ℓ*]→SE(2) yields

21 $$\begin{aligned}& \nabla_{\dot{\gamma}(s)} \Biggl(\sum_{k=1}^{3}\lambda_{k} \omega^{k} \Biggr) \\& \quad:= \sum_{k=1}^{3} \Biggl( \dot{\lambda}_{k}(s) + \sum_{i,j=1}^{3} c^{j}_{ik} \lambda_{j}(s) \dot{\gamma }^{i}(s) \Biggr) \omega^{k}\vert_{\gamma(s)} \end{aligned}$$

with $\dot{\lambda}_{k}(s)= \langle{\rm d}\lambda_{k}, \dot{\gamma }(s)\rangle$.

### Remark 5.1

The Christoffel symbols $c^{j}_{ki}$ of the Cartan connection ∇ on the tangent bundle *T*(SE(2)) expressed in reference frame $\{\mathcal{A}_{i}\}_{i=1}^{3}$ equal minus the structure constants on the Lie algebra. The Christoffel symbols of the corresponding Cartan connection on the co-tangent bundle *T* ^∗^(SE(2)) w.r.t. reference frame $\{\omega^{i}\}_{i=1}^{3}$ have opposite sign and are thereby equal to the structure constants $c^{j}_{ik}=-c^{j}_{ki}$.

Finally we mention the Cartan’s structural formula

22 $$\begin{aligned}& {\rm d}\omega^{k}= -\frac{1}{2} \sum_{i,j=1}^{3} c^{k}_{ij} {\rm d}\omega^{i} \wedge{\rm d}\omega^{j} =\sum_{i,j=1}^{3} c^{k}_{ji} \omega^{i} \otimes\omega^{j}, \end{aligned}$$

so for example for *k*=2 we find ${\rm d}\omega^{2}= {\rm d}(\cos\theta{\rm d}x+\sin\theta{\rm d}y)=-\sin\theta{\rm d}\theta\wedge{\rm d}x + \cos\theta{\rm d}\theta\wedge{\rm d}y= {\rm d}\theta\wedge{\rm d}\omega^{3}$.

Now that the preliminaries are done let us apply Bryant and Griffith’s method to **P**_curve_ in 4 steps.

**Step 1: Extend the manifold** **SE**(2) **with geometric control variables** Consider the extended manifold $Q= \mathrm{SE}(2)\times\mathbb{R}^{+} \times\mathbb{R}\times \mathbb{R}$ with coordinates (*x*,*y*,*e* ^*iθ*^,*σ*,*κ*,*r*), where *σ*=∥**x**′(*r*)∥ so that ${\rm d}s= \sigma{\rm d}r$, where *r*↦**x**(*r*) is some parametrization of the spatial part of the lifted curve *r*↦*γ*(*r*)=(**x**(*r*),*θ*(*r*)) in SE(2). In order to extend the sub-Riemannian manifold $(\mathrm{SE}(2),\mathrm{Ker}(\omega^{3}), \mathcal{G}_{\xi=1})$ such that the concept of horizontal curves is preserved we impose

23 $$ \begin{array}{l} \theta^{1}:={\rm d}\theta-\kappa\sigma{\rm d}r =0, \\ \theta^{2}:=\omega^{2}- \, \sigma{\rm d}r, \\ \theta^{3}:=\omega^{3}=0. \\ \end{array} $$

These equations determine the horizontal part

$$I(Q)=\bigl\{ v \in T(Q) \mid\theta^{1}(v)=\theta^{2}(v)= \theta^{3}(v)=0\bigr\} ^{*} $$

of the dual tangent space *T* ^∗^(*Q*). We have extended the sub-Riemannian manifold $(\mathrm{SE}(2),\mathrm{Ker}(\omega ^{3}),\mathcal {G}_{\xi=1})$ naturally to *I*(*Q*).

**Step 2: Include momentum** Include the Lagrange multipliers as local momentum vectors in our target space. Therefore we extend *Q* to a larger space *Z*. We define *Z* as the affine sub-bundle

$$Z=\{Z_\mathfrak{q} \mid\mathfrak{q} \in Q\}\equiv Q \times T\bigl(\mathrm{SE}(2) \bigr)^{*} $$

of *T* ^∗^(*Q*) determined by

$$Z_\mathfrak{q} = \bigl\{ \sqrt{\bigl(\kappa^{2} + 1 \bigr)}\, {\rm d}s \vert_{\mathfrak{q}} \in I_{\mathfrak{q}} \subset T^{*}_{\mathfrak{q}}(Q) \bigr\} , $$

which is isomorphic to *Z*≡*Q*×*T* ^∗^(SE(2)) via

24 $$ \begin{aligned} Q \times(T(\mathrm{SE}(2)))^{*} \ni\Biggl(\mathfrak{q},p:=\sum _{i=1}^{3}\lambda_{i}\omega^{i}\Biggr) \leftrightarrow\\ \psi \vert_{\mathfrak{q}}:= \sqrt{\kappa^{2} + 1} \, \sigma{\rm d}r \vert_{\mathfrak{q}} + \sum_{k=1}^3 \lambda_{k} \theta^{k} \vert_{\mathfrak{q}} \in Z \end{aligned} $$

**Step 3: Minimization on extended space** **Z** Consider a one parameter family {*N*_*r*_} of horizontal vector fields on SE(2) and compute the variation of the integrated Lagrangian-form *ψ* along such a *N*_*r*_:

25 $$\begin{aligned} \frac{d}{dr} \int_{N_r} \psi =& \int_{N_r} \mathcal{L}_{\frac {\partial}{\partial r}} \psi = \int_{N_r} \frac{\partial}{\partial r} \rfloor{\rm d}\psi+ \int _{N_r} {\rm d}\biggl(\frac{\partial}{\partial r} \rfloor\psi\biggr) \\ =& \int _{N_r} \frac{\partial}{\partial r} \rfloor{\rm d}\psi \end{aligned}$$

where we used the Stokes Theorem $\int_{N_{r}} {\rm d}(\frac{\partial }{\partial r} \rfloor\psi) = \oint_{\partial_{N_{r}}} \frac{\partial }{\partial r} \rfloor\psi=0$ and the formula for Lie derivatives of volume forms along vector fields $\mathcal{L}_{X}A=X \rfloor{\rm d }A + {\rm d}(X \rfloor A)$ and where *X*⌋*A*:=*A*(*X*,⋅) denotes the insert operator. Consequently, we must solve the canonical ODE system

26 $$ \varGamma'(r) \rfloor{\rm d\psi} \vert _{\varGamma(r)}=0 \quad\textrm{ for all }r>0. $$

where *Γ*(*r*)≡(*γ*(*r*),*κ*(*r*),*σ*(*r*),*r*,*p*(*r*)). This boils down to

27 $$ v \rfloor{\rm d}\psi= 0 \quad\textrm{for all }v \in T(Z). $$

Now by means of the Cartan structural formula (22), and Eq. (27) we obtain the Pfaffian system

28 $$ \left\{ \begin{array}{l} \partial_{\lambda_{1}} \rfloor{\rm d}\psi={\rm d}\theta- \kappa \sigma{\rm d}r=0 \\ \partial_{\lambda_{2}} \rfloor{\rm d}\psi=\omega^{2}- \sigma{\rm d}r=0 \\ \partial_{\lambda_3} \rfloor{\rm d}\psi=\omega^{3} =0 \\ \partial_{\sigma} \rfloor{\rm d}\psi= (\sqrt{\kappa^2 +1}-\lambda _{1}\kappa-\lambda_2) {\rm d}r =0 \\ \partial_{\kappa} \rfloor{\rm d}\psi= \sigma(\kappa(\kappa^2 + 1)^{-1/2} -\lambda_1 ) {\rm d}r =0 \\ -\partial_{\theta} \rfloor{\rm d}\psi= {\rm d}\lambda_{1} -\lambda _{2}\omega^{3} +\lambda_{3}\omega^{2} =0 \\ -\partial_{\xi} \rfloor{\rm d}\psi= {\rm d}\lambda_{2} -\lambda _{3}{\rm d}\theta=0 \\ -\partial_{\eta} \rfloor{\rm d}\psi= {\rm d}\lambda_{3} +\lambda _{2}{\rm d}\theta=0. \\ \end{array} \right. $$

The first three equations represent the horizontality restriction. The two equations in the middle represent the Euler-Lagrange optimization of the energy and show that {*λ*_1_,*λ*_2_,*λ*_3_} are components of momentum with respect to the dual frame (under identification (24)). It is readily deduced that

29 $$ \begin{array}{l} \lambda_{1}= \frac{\kappa}{\sqrt{\kappa^{2}+1}}=z,\quad\quad \lambda_{2}= \sqrt{1-z^2},\quad\quad \lambda_{3}=-\dot{z}. \end{array} $$

### Theorem 2

*Define* $L:= \sigma\sqrt{\kappa^{2}+1}$. *The Pfaffian system* (28) *for*

$$\varGamma(\cdot)=\bigl(\gamma(\cdot),\kappa(\cdot),\sigma(\cdot ),p(\cdot) \bigr): [0,\ell] \to Z $$

*with* *γ* *a cuspless sub*-*Riemannian geodesic can be rewritten as*

30 $$\begin{aligned}& \begin{aligned} &\theta^{1}=\theta^{2}=\theta^{3}=0, \\ &p={\rm d}L(\sigma\kappa, \sigma), \\ &\nabla p =0, \end{aligned} \end{aligned}$$

*where* ∇ *denotes the Cartan connection on the co*-*tangent bundle* *T* ^∗^(SE(2)).

### Proof

The last 3 equations in (28) provide the momentum covector. They can be written as

31 $$ {\rm d}\lambda_{i}+\sum _{j,k=1}^{3}c^{k}_{ij} \lambda_{k} \cdot\omega^{j}=0 , \quad i=1,2,3, $$

which by Eq. (21) can be rewritten as

32 $$ \nabla p=0, \quad\quad p=\sum_{i=1}^{3} \lambda_{i}\omega^{i}. $$

To this end we note that

33 $$\begin{aligned}& \forall_{i\in\{1,2,3\}}:\langle{\rm d}\lambda_{i}, \dot{\gamma } \rangle+\sum_{j,k=1}^{3}c^{k}_{ij} \lambda_{k} \langle \omega^{j} \vert_{\gamma}, \dot{\gamma} \rangle=0 \\& \quad \Leftrightarrow\quad \nabla_{\dot{\gamma}(s)} p=0. \end{aligned}$$

Finally, with respect to the second part of Eq. (30):

$$\begin{aligned} {\rm d}L =& \sigma^{-1} \frac{\partial L}{\partial\kappa} {\rm d}(\sigma\kappa) + \biggl(\frac{\partial L}{\partial\sigma} -\sigma^{-1}\kappa\frac {\partial L}{\partial\kappa} \biggr) {\rm d}\sigma\\ =&\frac{\kappa}{\sqrt{\kappa^{2}+1}}{\rm d}(\sigma\kappa)+ \frac {1}{\sqrt{\kappa^{2}+1}} {\rm d\sigma} =\lambda_{1} {\rm d}(\sigma \kappa) + \lambda_{2} {\rm d}\sigma, \end{aligned}$$

from which the result follows. □

### Remark 5.2

The first part ensures *γ*=(**x**,*θ*) is the horizontal lift from the planar curve **x**(*s*)=(*x*(*s*),*y*(*s*)), i.e. $\theta(s)=\arg(\dot{x}(s)+i \dot{y}(s))$. The second part allows us to interpretate $p=\sum_{i=1}^{3} \lambda_{i} \omega^{i}$ as a momentum covector.

### Remark 5.3

In contrast to Levi-Civita connections on Riemannian manifolds, the Cartan connection ∇ has torsion and thereby auto-parallel curves do not coincide with geodesics. In fact, Theorem 12 in Appendix C shows that auto-parallel curves are (horizontal) exponential curves.

**Step 4: Integrate the Pfaffian system** To integrate $\nabla_{\dot{\gamma}}p=0$ we resort to matrix-representation $m:\mathrm{SE}(2) \to\mathbb{R}^{3\times3}$ given by

34 $$ \begin{array}{l} m(\mathbf{x},R_{\theta})= \left( \begin{array}{cc} R_{\theta} & \mathbf{x} \\ 0 & 1 \end{array} \right)\quad \textrm{with } \\ R_{\theta}= \left( \begin{array}{cc} \cos\theta& -\sin\theta\\ \sin\theta& \cos\theta \end{array} \right) \quad\textrm{and}\quad\mathbf{x}=(x,y)^{T}, \end{array} $$

and express dual-vectors (covectors) as row vectors. Analogously to Bryant’s work on elastica [19] we express equation (32) in explicit coordinates

35 $$ {\rm d}\hat{\lambda}= \hat{\lambda} \bigl(m(\gamma) \bigr)^{-1}{\rm d}m(\gamma) $$

where we use short-notation for the row-vector

36 $$ \hat{\lambda}:=(-\lambda_{3},\lambda_{2}, \lambda_{1}), $$

from which we deduce that

37 $$\begin{aligned} \begin{aligned} \nabla p=0\quad &\Leftrightarrow\quad{\rm d} (\hat{\lambda} m(\gamma^{-1})) = 0 \\ &\Leftrightarrow\quad \hat{\lambda} m(\gamma^{-1}) = \hat{\lambda}(0) m(\gamma^{-1}(0)). \end{aligned} \end{aligned}$$

Before we will derive *γ* from Eq. (37) we will need the following lemma based on Noether’s theorem. Formally, one can avoid this general abstract lemma (as in [19]) by observing

$$\begin{aligned} \nabla p= 0 \quad \Rightarrow&\quad\lambda_{2} {\rm d}\lambda_{2} +\lambda_{3} {\rm d}\lambda_{3}=0 \\ \Rightarrow&\quad |\lambda_{2}|^2 +|\lambda_{3}|^2=|\lambda_{2}(0)|^2 +|\lambda _{3}(0)|^2=:\mathfrak{c}^2. \end{aligned}$$

### Lemma 2

*Cuspless sub*-*Riemannian geodesics are contained within the co*-*adjoint orbits*

38 $$ \mathfrak{c}^2=|\lambda_{2}(s)|^2+| \lambda_{3}(s)|^2= |\dot{z}(s)|^2 + 1-|z(s)|^2, $$

*for all* *s*∈[0,*s*_max_], *with* *s*_max_ *given by Eq*. (41).

### Proof

According to Noether’s theorem (i.e. conservation law on momentum) the moment map *m*:*Z*→*T*(SE(2))^∗^ given by $\langle m(\mathfrak{q},p), \varXi\rangle= (\varXi\rfloor\psi )(\mathfrak{q},p)$ with $(\mathfrak{q},p) \in Z\equiv Q \times T^{*}(\mathrm {SE}(2))$, for all *Ξ*∈*T*(SE(2)) is constant along the characteristic curves $\varXi=\dot{\gamma}$. The co-adjoint representation of SE(2) acting on the dual of its Lie-algebra (*T*(SE(2)))^∗^ is given by $\langle(\mathrm{Ad}_{g^{-1}})^{*}p ,\varXi\rangle= \langle p, \mathrm {Ad}_{g} \varXi\rangle$, i.e.

39 $$\begin{aligned} (\mathrm{Ad}_{g^{-1}})^{*}(p) =&(\lambda_{1}+\lambda_{2} y - x \lambda_{3})\omega^{1} \\ &{} +(\lambda_{2} \cos(\theta) +\lambda_{3}\sin\theta) \omega^{2} \\ &{} + (\cos(\theta)\lambda_{3}- \lambda_{2} \sin(\theta) ) \omega^{3}. \end{aligned}$$

We have $m(\eta_{g}(\mathfrak{q},p))= (\mathrm{Ad}_{g^{-1}})^{*} m(\mathfrak {q},p)$, where the group action *g*↦*η*_*g*_ is given by

$$\eta_{g}\bigl(g',\kappa, \sigma,r,p\bigr)=\bigl(g g', \kappa,\sigma,r, (\mathrm{Ad}_{g^{-1}})^{*}p \bigr). $$

As a result the co-adjoint orbits of SE(2) coincide with the cylinders in Eq. (38). □

### Corollary 1

*From Eq*. (38) *we deduce that*

40 $$ \ddot{z}(s) = z(s) \quad\Rightarrow\quad z(s)=z_{0} \cosh(s) + \dot{z}_{0} \sinh(s). $$

*The minimizers of* **P**_curve_ *are cuspless geodesics and their total length* (*towards a cusp*) *equals*

41 $$ s_{\mathrm{max}}:= \log\biggl(\frac{1+\mathfrak {c}}{|z_0+ \dot {z}_{0}|} \biggr) \in \mathbb{R}^{+} \cup\{\infty\}. $$

*The curvature of orbits with* $\mathfrak{c}<1$ *and* *z*_0_>0 *is strictly positive*. *The curvature of orbits with* $\mathfrak{c}<1$ *and* *z*_0_<0 *is strictly negative*. *The curvature of orbits with* $\mathfrak{c}>1$ *switches sign once at*

42 $$ s_{B}= \log\biggl(\frac{\sqrt{\mathfrak {c}^{2}-1}}{|z_{0}+ \dot {z}_{0}|} \biggr)\leq2 s_{\mathrm{max}} $$

### Proof

Follows directly from the hyperbolic phase portrait induced by $\ddot{z}=z$ and Theorem 2, and solving for respectively |*z*(*s*)|=1 and *z*(*s*)=0. □

After these results on sub-Riemannian geodesics, we continue with solving for ∇*p*=0, Eq. (37). Problem **P**_curve_ is left-invariant and in the next lemma we select a suitable point on each co-adoint orbit to simplify the computations considerably.

### Lemma 3

*Let* $\mathfrak{c}>0$. *There exists a unique* *h*_0_∈SE(2) *such that* $\hat{\lambda}(0) m(h_{0}^{-1}) = (\mathfrak{c},0,0)$. *Consequently*, *we have for* $\tilde{\gamma}(s):=h_{0}\gamma(s)$ *that*

43 $$\begin{aligned}& \nabla p =0 \\& \quad \Rightarrow\quad(-\lambda_{3}(s),\lambda_{2}(s),\lambda _{1}(s)) =\hat{\lambda}(s)=(\mathfrak{c}\ 0 \ 0)\quad m(\tilde{\gamma}(s)). \end{aligned}$$

### Proof

Equation (43) follows by Eq. (37) and the fact that *m* (Eq. (34)) is a group representation. □

Applying the above Lemma and Eq. (29) provides the next theorem, Theorem 3, where we provide explicit analytical formulae for the geodesics by integration of the Pfaffian system. To this end we first need a formal definition of the operator that integrates the Pfaffian system Eq. (28) and produces the corresponding geodesic of **P**_curve_ in SE(2).

This operator needs initial momentum *p*_0_ and total spatial length *ℓ*>0 as input and produces the corresponding geodesic of **P**_curve_ as output. By Eqs. (29) and (32) initial momentum equals

44 $$ p_0= z_0 {\rm d}\theta+ \sqrt{1-|z_0|^{2}} {\rm d}x -\dot{z}_{0}{\rm d}y, $$

with initial normalized curvature $z_{0}=\kappa_{0}/\sqrt{\kappa_{0}^{2}+1}$. As a result, we have

$$\kappa_0(p_0)=\frac{\langle p_0,\partial_\theta\rangle}{\sqrt{1- |\langle p_0,\partial_\theta\rangle|^{2}}}= \frac{\lambda _{1}(0)}{\sqrt{1-|\lambda_{1}(0)|^2}}. $$

The Hamiltonian at the unity element, evaluated at initial momentum is given by

$$H(e,p_0)=\frac{|\lambda_{1}(0)|^{2}+|\lambda_{2}(0)|^2}{2}. $$

Now let us use arclength parameterization (so set *r*=*s* and *σ*=1) in the canonical ODE system (26) on *Z*. Via identification Eq. (24) this gives rise to an equivalent ODE system on *Q*×*T* ^∗^(SE(2))

45 $$ \left\{ \begin{array}{l} \dot{\gamma}(s)= F(\gamma(s)), \qquad s \in[0,\ell], \\ \dot{\gamma}(0)= (e, 1, \kappa(p_0), 0, p_0) \in Q \times T^{*}(\mathrm{SE}(2)), \end{array} \right. $$

with unity element *e*=(0,0,0)∈SE(2).

### Definition 2

Let *γ*(*s*)=*e* ^*sF*^(*γ*(0)),*s*∈[0,*ℓ*] denote the unique solution of ODE (45). Now in view of Eq. (8) and Lemma 2 we define

46 $$\begin{aligned}& \begin{aligned} &C:= \biggl\{ p_0 \in T^{*}_{e}(\mathrm{SE}(2))\mid H(e,p_0)=\frac{1}{2}, p_0 \neq\pm{\rm d}\theta\biggr\} , \\ &\mathcal{D}:= \biggl\{ (p_0,\ell) \in C \times\mathbb{R}^{+}\mid p_0 \in C, \ell \leq s_{max}(p_0) \biggr\} \end{aligned} \end{aligned}$$

and we define $\widetilde{\mathrm{Exp}}_{e}: \mathcal{D} \to\mathrm {SE}(2)$ by

47 $$ \widetilde{\mathrm{Exp}}_{e}(p_0,\ell):= \pi\circ e^{\ell F}\bigl(e,1,\kappa(p_0),0,p_0\bigr). $$

where *π*:*Q*×*T* ^∗^(SE(2))→SE(2) is the natural projection given by *Π*(*g*,1,*κ*,*s*,*p*)=*g* for all $g \in\mathrm{SE}(2), \kappa ,s>0, p \in T^{*}_{g}(\mathrm{SE}(2))$.

### Remark 5.4

For sober notation we omit index *e* and write $\widetilde{\mathrm{Exp}}=\widetilde{\mathrm{Exp}}_{e}$ and *H*(*p*)=*H*(*e*,*p*) for exponential map and Hamiltonian. Furthermore, we include a tilde in this exponential map associated to the geometrical control problem of **P**_curve_ to avoid confusion with the exponential map Exp:*T*_*e*_(SE(2))→SE(2) from Lie-algebra to Lie group.

### Remark 5.5

The dual vectors $p_{0}= \pm{\rm d}\theta$ are not part of the domain of the exponential map as in these cases one would have $(z_{0},\dot{z}_{0})=(\pm1,0)=(z(s),\dot{z}(s))$ for all *s*≥0 and the sub-Riemannian geodesics in SE(2) propagate only in vertical direction, not allowing spatial arc-length parameterization. See also [16, Remark 31].

### Theorem 3

*The exponential map* (*given by Eq*. (47)) *expressed in spatial arc*-*length parametrization is given by*

48 $$ \widetilde{\mathrm{Exp}} \Biggl(\sum_{i=1}^{3}\lambda_{i}(0) \omega^{i}\vert_{\gamma(0)=e}, s \Biggr) = \gamma(s)=(x(s),y(s),\theta(s)), $$

*with* *λ*_1_(0)=*z*_0_, $\lambda_{2}(0)=\sqrt{1-|z_{0}|^{2}}$, $\lambda_{3}(0)=-\dot{z}_{0}$, *and* *s*∈[0,*ℓ*] *with total spatial length* *ℓ*≤*s*_max_ *less than the spatial cusp*-*length Eq*. (41).

*Here the cuspless geodesics are given by* $\gamma(s)= h_{0}^{-1} \tilde {\gamma}(s)$, *i*.*e*.

49 $$ \begin{aligned} &\theta(s)=\tilde{\theta}(s) - \overline{\theta}_{0} \in[-\pi,\pi ], \\ &\quad\textit{with }\cos(\overline{\theta}_{0})= \frac{\dot {z}_{0}}{\mathfrak{c}} \textit{ and }\overline{\theta}_{0}\in[-\pi ,0] , \\ &\mathbf{x}(s)= \overline{R}_{0}^{T}(\tilde{\mathbf{x}}(s)-\overline {\mathbf {x}}_{0}), \\ &\quad\textit{with }\overline{R}_{0}^{T}= \left( \begin{array}{cc} \cos\overline{\theta}_{0} & \sin\overline{\theta}_{0} \\ -\sin\overline{\theta}_{0} & \cos\overline{\theta}_{0} \end{array} \right) \\ &\phantom{\quad\textit{with }\overline{R}_{0}^{T}}=\frac{1}{\mathfrak{c}} \left( \begin{array}{cc} \dot{z}_{0} & - \sqrt{1-|z_{0}|^2} \\ \sqrt{1-|z_0|^2} & \dot{z}_{0} \end{array} \right) \end{aligned} $$

*with* $h_{0}=(\overline{\mathbf{x}}_{0},\overline{R}_{0}) \in\mathrm {SE}(2)$, *with* $\overline{\mathbf{x}}_{0}=(\frac{z_{0}}{\mathfrak{c}},0)^{T}$.

*Here curve* $\tilde{\gamma}=(\tilde{x},\tilde{y},\tilde{\theta})$ *is given by*

50 $$ \begin{aligned} &\tilde{x}(s) = \frac{z(s)}{\mathfrak{c}}= \frac{z_{0} \cosh(s) + \dot{z}_{0} \sinh(s)}{\mathfrak{c}}, \\ &\tilde{y}(s) = -\frac{1}{\mathfrak{c}} \int_{0}^{s} \sqrt {1-|z(\tau)|^2}\, {\rm d}\tau,\\ &\tilde{\theta}(s) = \arg(\dot{\tilde{x}}(s) + i \dot{\tilde {y}}(s)) \\ &\phantom{\tilde{\theta}(s)}=\arg( \dot{z}(s) -i\sqrt{1-|z(s)|^2} ) \in[-\pi,0], \end{aligned} $$

*where* $\mathfrak{c} \geq0$ *is given by*

51 $$ \mathfrak{c}=\sqrt{1- |z_{0}|^2 + | \dot{z}_{0}|^2}. $$

### Proof

Follows by Lemma 3 and Eq.’s (44), (29). □

Note that the cuspless geodesic *γ* follows from cuspless geodesic $\tilde{\gamma}=h_{0} \gamma$ via the rigid body motion

$$\begin{aligned} \gamma= h_{0}^{-1}(\tilde{\mathbf{x}},\tilde{\theta})= (R_{\overline{\theta}_{0}}^{-1}(\tilde{\mathbf{x}}-\overline {\mathbf {x}}_{0}),\tilde{\theta}-\overline{\theta}_{0}). \end{aligned}$$

### Corollary 2

*The end*-*point* *g*_*fin*_ *of a cuspless sub*-*Riemannian geodesic is given by*

52 $$ \begin{aligned} &x_{fin}=\frac{(z(\ell)-z_0)\dot{z}_{0}}{\mathfrak{c}^2} + \frac {\sqrt{1-|z_0|^2}}{\mathfrak{c}^2} \int_{0}^{\ell} \sqrt {1-(z(s))^2}\, {\rm d}s \\ &y_{fin} = \frac{\sqrt{1-|z_0|^2}(z(\ell)-z_0)}{\mathfrak{c}^2}- \frac{\dot {z}_0}{\mathfrak{c}^2} \int_{0}^{\ell} \sqrt{1-|z(s)|^2}\, {\rm d}s \\ &\theta_{fin}= \arg\Bigl\{ \Bigl(\dot{z}(\ell)\dot{z}_0 + \sqrt{1 - |z(\ell )|^2}\sqrt{1 - |z_0|^2} \Bigr) \\ &\phantom{\theta_{fin}=}{}+ i ( \dot{z}(\ell)\sqrt{1 - |z_0|^2}- \dot{z}_0\sqrt{1 - |z(\ell)|^2} ) \Bigr\} . \end{aligned} $$

### Proof

From the previous Theorem 3 we deduce

$$\begin{aligned} \theta_{fin}=\theta(\ell)=\tilde{\theta}(\ell)-\overline{\theta }_0 \end{aligned}$$

and

$$\begin{aligned} \mathbf{x}_{fin}=\mathbf{x}(\ell) = \overline{R}_{0}^{T} (\tilde{\mathbf{x}}(\ell) - \overline{\mathbf{x}}_0) \end{aligned}$$

from which the result follows. □

### Corollary 3

*The* (*x*,*y*)-*coordinates of the Exponential map involve one elliptic integral and the tangent vectors along geodesics do not involve any special functions*. *Furthermore*, *from* $-\dot{\tilde{y}}(s) \geq0$ *it follows that the spatial part of the geodesics is monotonically increasing along the* $(-\sin\overline {\theta}_{0}, -\cos\overline{\theta}_{0})=\frac{1}{\mathfrak{c}}(\sqrt {1-|z_{0}|^{2}},-\dot{z}_{0})$-*axis*:

$$\sqrt{1-|z_{0}|^2} \dot{x}(s)-\dot{z}_{0} \dot{y}(s) \geq0. $$

Geodesics with $\mathfrak{c}=1$ admit simple formulas:

### Corollary 4

*In the critical case* $\mathfrak{c}=1$ *and* $\dot{z}_{0}=-z_{0}$ *we find* *s*_max_=∞ *and*

$$\begin{aligned} &\overline{\theta}_{0}=-\arccos(-\dot{z}_0) \in[-\pi,0], \\ &\overline{\mathbf{x}}_{0}=(z_0,0)^{T}, \\ &\tilde{x}(s)= z_0 e^{-s}, \\ &\tilde{y}(s)= -s + \sqrt{1- e^{-2s} |z_0|^2}-\sqrt{1- |z_0|^2} \\ &\phantom{\tilde{y}(s)=}{}- \log\biggl( \frac{1+\sqrt{1-|z_0|^2e^{-2s}}}{1+\sqrt{1-|z_0|^2}} \biggr), \\ &\tilde{\theta}(s)= \arg\Bigl(-z_0 e^{-s} -i \sqrt{1- |z_0|e^{-2s}}\Bigr). \end{aligned} $$

*For* *s*→∞ *solutions converge towards the* $-\tilde{y}$-*axis*. *Geodesic* *γ*(*s*) *now follows by Eq*. (79).

### Corollary 5

*In the critical case* $\mathfrak{c}=1$ *and* $\dot{z}_{0}=z_{0}$ *we find* *s*_max_=−log|*z*_0_| *and*

$$\begin{aligned} &\overline{\theta}_{0}=-\arccos(\dot{z}_0) \in[-\pi,0], \\ &\overline{\mathbf{x}}_{0}=(z_0,0)^{T}, \\ &\tilde{x}(s)= z_0 e^{s}, \\ &\tilde{y}(s)= \sqrt{1-|z_0|^2} -\sqrt{1-|z_0|^2 e^{2s}}\\ &\phantom{\tilde{y}(s)=}{}-\mathrm{{arctanh}}(\sqrt{1-|z_0|^2}) + \mathrm{{arctanh}}(\sqrt{1-|z_0|^2e^{2s}}), \\ &\tilde{\theta}(s)= \arg(z_0 e^{s} -i \sqrt{1- |z_0|e^{2s}}). \end{aligned} $$

*Geodesic* *γ*(*s*) *now follows by Eq*. (49).

**Fig. 10:**
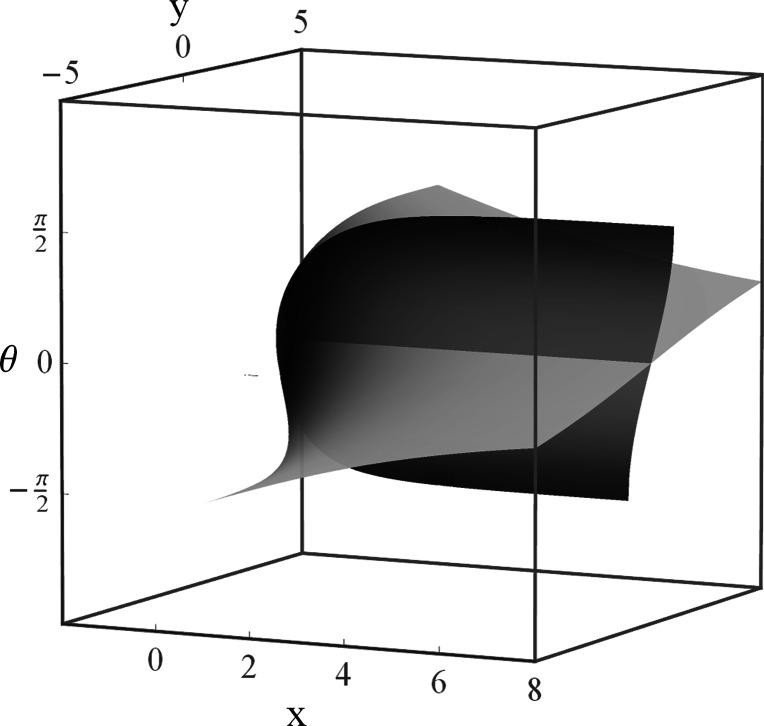
The critical surface is the union of the two surfaces generated by the solutions derived in Corollaries 4 and 5 that cross at the positive *x*-axis

For a plot of the critical surface see Fig. 10.

### Relation Between the Exponential Mappings of **P**_curve_ and **P**_MEC_

In Theorem 3 we have derived the exponential map of **P**_curve_ in terms of spatial arc-length parametrization *s*, whereas in previous work [15] the exponential map of **P**_MEC_ is expressed in sub-Riemannian arc-length *t*. For comparison see Appendix B.

On the one hand one observes that the exponential map of **P**_curve_ is much simpler when expressed in *s* and it is easier to integrate in current active shape models in imaging where the same kind of parametrization is used. On the other hand for **P**_MEC_ it is more natural to choose *t*-parametrization as this parametrization does not beak down at cusps. The following theorem relates the exponential mappings for **P**_curve_ and **P**_MEC_.

#### Theorem 4

*Let* $\widetilde{\mathrm{Exp}}$ *denote* 9 *the exponential map of* **P**_curve_. *Let* $\widetilde{\mathrm{EXP}}$ *denote the exponential map of* **P**_MEC_. *Then these exponential maps satisfy the following relation*

53 $$ \widetilde{\mathrm{EXP}}\bigl(p_0,T=t( \ell,p_0)\bigr)= \widetilde{\mathrm{Exp}}(p_0,\ell) $$

*for all* $p_{0}\in C \subset T^{*}_{e}(\mathrm{SE}(2))$, *and all* 0<*ℓ*≤*s*_*max*_, (*so that* $(p_{0},\ell) \in\mathcal{D}$, *recall Eq*. (46)), *where* *t*(*ℓ*,*p*_0_) *is given by Eq*. (6).

#### Proof

We note that *ℓ*≤*s*_*max*_ implies that the orbits do not hit the cusp lines in the pase portraits (i.e. |*z*|=1 and *ν*=0,2*π*) so that (*ν*(*t*),*c*(*t*)) stays within the central strip (i.e. *ν*(*t*)∈[0,2*π*]) indicated in Fig. 9. The rest follows by Lemma 1. □

## The Set $\mathcal{R}$ and the Cusp-Surface $\partial \mathcal {R}$

According to Theorem 1 the set of points in SE(2) that can be reached with a global minimizer from unity element *g*_*in*_=*e*=(0,0,0) is equal to $\mathcal {R}$ given in Definition 1. Therefore, we first need to investigate this set in order to apply cuspless sub-Riemannian geodesics in vision applications. First of all we have the following characterization.

### Theorem 5

*Let* *s*_*max*_(*p*_0_) *be given by Eq*. (41). *Let* *C* *be given by Eq*. (46). *The range of the exponential map given by*

54 $$ \{\widetilde{\mathrm{Exp}}(p_0,\ell) \mid 0 < \ell\leq s_{max}(p_0) \textit{ and } p_0 \in C \subset T_{e}^{*}(\mathrm{SE}(2)) \}, $$

*coincides with the set* $\mathcal{R}$, *consisting of points in* SE(2) *that can be reached with* (*globally minimizing*) *geodesics of* **P**_curve_ *departing from* *e*.

### Proof

Apply Theorems 1 and 3, where the analytic stationary solution curves of **P**_curve_ break down iff *ℓ*=*s*_*max*_(*p*_0_) in which case tangents to geodesics are vertical due to $|z(\ell)|=\frac{d\theta}{dt}(T)=1$. □

The exponential map of **P**_curve_ coincides with the exponential map of **P**_MEC_ [2, 47] restricted to the strip *ν*(*t*(*s*))∈[0,2*π*] (in between the blue lines in Fig. 9), where we exclude the points (*ν*,*c*)=(0,0) and (*ν*,*c*)=(2*π*,0) from the strip (recall Remark 5.5) so that in the range we exclude the vertical line

$$\mathfrak{l}:=\bigl\{ (0,0,\theta)\mid-\pi\leq\theta\leq\pi\bigr\} . $$

The exponential map of **P**_MEC_ restricted to this strip is a homeomorphism (as follows by the results in [56]) thereby the exponential map of **P**_curve_ is a homeomorphism as well. As a result (for formal proof see Appendix F) we have

### Theorem 6

*Let* $\mathcal{D}, \mathcal{R}$ *denote respectively the domain and range of the exponential map of* **P**_curve_ (*recall Eqs*. (46), (54)). *Then*

- $\widetilde{\mathrm{Exp}}: \mathcal{D} \to\mathcal{R}$ *is a homeomorphism if we equip* $\mathcal{D}$ *and* $\mathcal{R}$ *with the subspace topology*.^10^
- $\widetilde{\mathrm{Exp}}: \mathring{\mathcal{D}} \to \mathring {\mathcal{R}}$ *is a diffeomorphism*.

*Finally*, *the boundary* $\partial\mathcal{R}$ *is given by*

55 $$\begin{aligned}& \partial \mathcal{R}= \{\widetilde{\mathrm{Exp}}(p_0,s_{max}(p_0))\mid p_0 \in C \} \\& \phantom{\partial\mathcal{R} = }{} \cup\mathfrak{l} \cup \bigl\{ \widetilde{\mathrm{Exp}}(p_0,s)\mid p_0 \in C \textrm{ with } z_0=\pm1 , \\& \phantom{\partial\mathcal{R}=\ \ \ \quad\quad} \textit{and }s \in(0, s_{max}(z_0,\dot{z}_0)) \bigr\} \end{aligned}$$

**Fig. 11:**
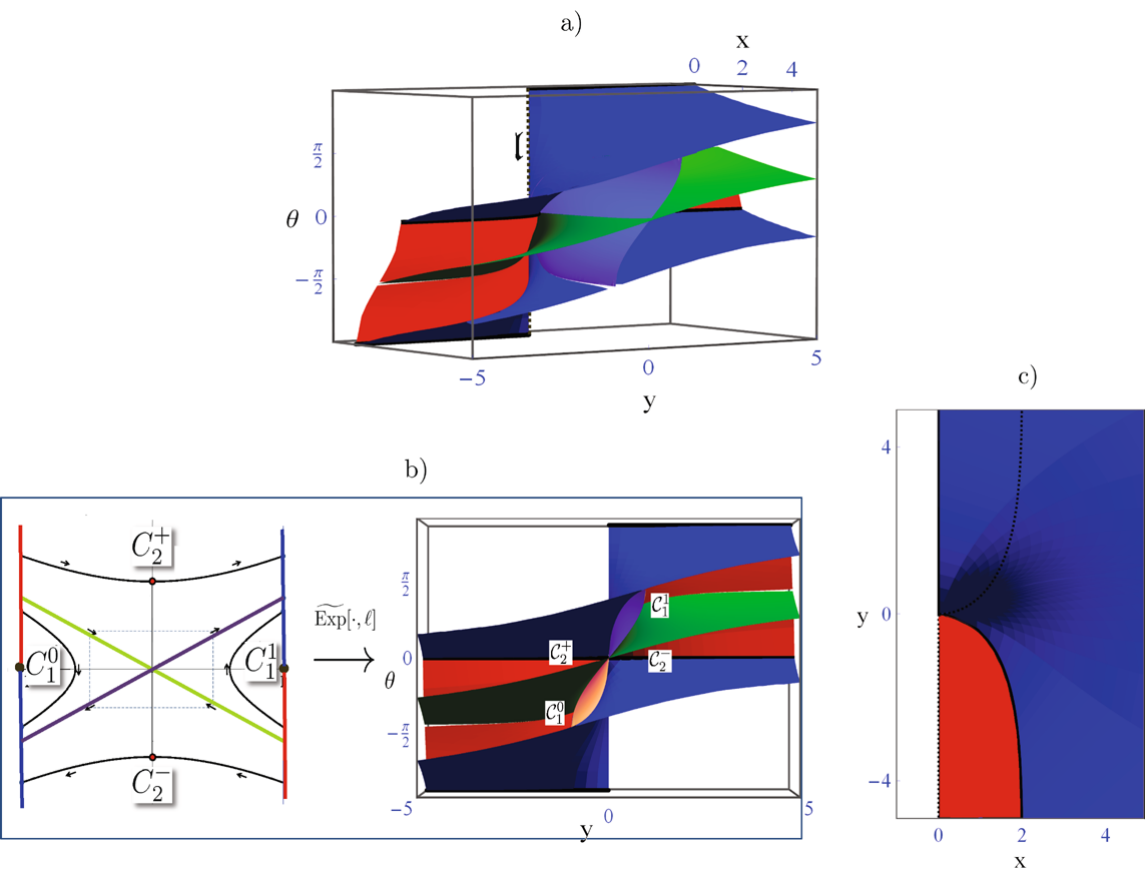
Plots (from 3 different perspectives (**a**), (**b**) and (**c**)) of the range $\mathcal{R}$ of the exponential map of **P**_curve_. *Red surface*: endpoints of geodesics starting from cusp. *Blue surface*: endpoints of geodesics ending in cusp. The *black lines* are the intersections of the *blue surface* with the *red surface*. *Green surface*: critical surface ($\mathfrak{c}=1$) with $\dot{z}_{0}=-z_{0}$. *Purple surface*: critical surface ($\mathfrak{c}=1$) with $\dot{z}_{0}=z_{0}$. The critical surface splits the range of the exponential map into four disjoint parts $\mathcal{C}_{1}^{1}$, $\mathcal{C}^{0}_{1}$, $\mathcal{C}_{2}^{+}$ and $\mathcal{C}_{2}^{-}$ that directly relate to the splitting of the phase space, cf. 9 into $C_{1}^{1}$, $C^{0}_{1}$, $C_{2}^{+}$ and $C_{2}^{-}$ as shown in (**b**) where we have depicted $\mathcal{R}$ viewed from the *x*-axis. In (**c**) we have depicted $\mathcal{R}$ viewed from the *θ*-axis (Color figure online)

These results can be observed in Fig. 11, which shows a well-posed, smooth, bijective relation between smooth regions in the phase portrait (i.e. $\mathcal{D}$) and smooth regions in $\mathcal {R}\subset\mathrm{SE}(2)$ and where the union of the blue and red surfaces form the cusp-surface adjacent to the line $\mathfrak{l}$. Subsequently, we provide some theorems on $\mathcal{R}$ and $\partial \mathcal{R}$ to get a better grip on the existence set of **P**_curve_, recall Eqs. (3) and (11).

### The Elliptic Integral in the Exponential Map

In this section we will first express the single elliptic integral arising in the exponential map in Theorem 3 in a standard elliptic integral and then we provide bounds for this integral from which one can deduce bounds on the set $\mathcal{R}$.

#### Lemma 4

*The elliptic integral in Theorem *3 *can be rewritten as*

$$\begin{aligned}& \int_{0}^{s} \sqrt{1-|z(\tau)|^{2}} {\rm d}\tau\\& \quad= -i \frac{\sqrt{1+\mathfrak{c}^2}}{\sqrt{2}} \sqrt{1-\delta} \biggl(E\biggl((s+\varphi)i, \frac{2\delta}{\delta-1}\biggr)\\& \quad\quad{}- E\biggl(\varphi i, \frac{2\delta}{\delta-1}\biggr) \biggr), \end{aligned}$$

*with* $\delta= \sqrt{|c_{1}|^{2}-|c_{2}|^{2}}\leq1$ *and* $\varphi=\frac {1}{4} \log\frac{c_{1}+c_{2}}{c_{1}-c_{2}}$, *with* $c_{1}=\frac{|z_{0}|^{2} +|\dot{z}_{0}|^{2}}{1+\mathfrak{c}^{2}}$, $c_{2}=\frac{2 z_{0} \dot{z}_{0}}{1+\mathfrak{c}^{2}}$ *and where*

56 $$ E(z,m)= \int_{0}^{z} \sqrt{1- m \sin^{2}(v)} {\rm d}v $$

*denotes the elliptic integral of the second kind*.

#### Proof

Using Eq. (77) and Eq. (38) we find $1-|z(\tau)|^{2}= \frac{1+\mathfrak{c}^{2}}{2} (1- c_{1}\cosh(2\tau) - c_{2} \sinh(2\tau))$ from which the result follows via *v*=*iτ*. □

For explicit bounds for the elliptic integral for the cases $\mathfrak {c}<1$, where the sub-Riemannian geodesics are U-shaped, see Appendix H.

### Observations and Theorems on $\mathcal{R}$

In Theorem 3 we have derived the exponential map of **P**_curve_ in explicit form. Before we derive some results on the range $\mathcal{R}$ of the exponential map we refer to Fig. 11 where we have depicted the set $\mathcal {R}$ using Theorem 3. In Fig. 11 we observe:

1. The range $\mathcal{R}$ of the exponential map is a connected, non-compact set and its piecewise smooth boundary coincides with the cusp-surface, Eq. (55).
2. The range of the exponential map produces a reasonable criterium (namely condition (3)) to connect two local orientations. Consider the set of reachable cones depicted in Fig. 14.
3. The range of the exponential map of **P**_curve_ is contained in the half-space *x*_*fin*_≥0 and |*θ*_*fin*_|=*π* can only be attained at *x*=0 and *y*≠0 where geodesics arrive at a cusp.
4. The cone of reachable angles *θ*_*fin*_ per position $(x_{fin}, y_{fin}) \in\mathbb{R}^{+} \times\mathbb{R}^{+}$, with $(x_{fin},y_{fin},\theta_{fin}) \in\mathcal{R}$ is either given by
  57 $$ \begin{aligned} &[\theta_{\mathrm{begincusp}}(\mathbf{x}_{fin}), \theta _{\mathrm{endcusp}}(\mathbf{x}_{fin})]\quad \textrm{or by }\\ &[\theta_{\mathrm{endcusp}}^{1}(\mathbf{x}_{fin}), \theta _{\mathrm{endcusp}}^{2}(\mathbf{x}_{fin})], \end{aligned} $$
  with **x**_*fin*_=(*x*_*fin*_,*y*_*fin*_) where *θ*_endcusp_(**x**_*fin*_) denotes the final angle of the geodesic ending in (**x**_*fin*_,⋅) with a cusp, and where *θ*_begincusp_(**x**_*fin*_) denotes the final angle of a geodesic ending in (**x**_*fin*_,⋅) starting with a cusp. In the second case there exist two geodesics ending in **x**_*fin*_ with a cusp and we index these such that $\theta_{\mathrm {endcusp}}^{1}<\theta_{\mathrm{endcusp}}^{2}$. Which of the two options applies depends on $\mathbf{x}_{fin} \in \mathbb{R} ^{2}$. See Fig. 12.

**Fig. 12:**
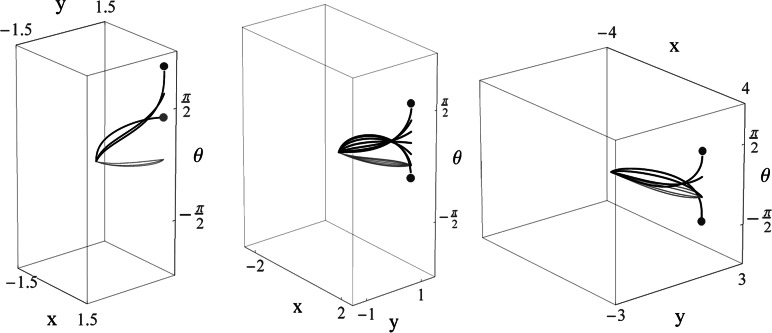
Sub-Riemannian geodesics (and their spatial projections in grey) obtained by our analytical approach to the boundary value problem, cf. Theorem 11. We have kept (*x*_*fin*_,*y*_*fin*_) fixed and we have varied *θ*_*fin*_ to full range such that our algorithm finds solutions (with relative errors less than 10^−8^). *Left*: (*x*_*fin*_,*y*_*fin*_)=(1,1.5), *middle*: (*x*_*fin*_,*y*_*fin*_)=(2,1), *right*: (*x*_*fin*_,*y*_*fin*_)=(4,1). We observe (when approaching a cusp we have vertical tangent vectors in SE(2)) that in (*x*_*fin*_,*y*_*fin*_)=(1,1.5) the first case in Eq. (57) applies, whereas in (*x*_*fin*_,*y*_*fin*_)=(2,1),(4,1) the second case in Eq. (57) applies. At the boundary of cones of reachable angles, the end-points of the sub-Riemannian geodesics are located on the cusp-surface $\partial \mathcal{R}$. End-points of geodesics departing from a cusp are indicated in red and end points of geodesics ending at cusp are indicated in red (likewise Fig. 11) (Color figure online)
5. The boundary of the range of the exponential map (given by Eq. (55)) is smooth except for 3 intersections between the surface induced by end-points of geodesics starting from a cusp and the surface induced by end-points of geodesics ending at a cusp. These intersections are given by
  $$\begin{aligned}& \theta_{fin}=-\pi\quad\textrm{and}\quad x_{fin}=0 \quad\textrm{and}\quad y_{fin} \leq 0, \\& \theta_{fin}=0 \quad\textrm{and }\\& |y_{fin}|= -x_{fin} i E \biggl(i \,\mathrm{arcsinh}\, \frac{x_{fin}}{\sqrt{4-x_{fin}^2}}, 1-\frac{4}{x_{fin}^2} \biggr), \\& \textrm{and}\quad 0\leq x_{fin}<2, \\& \theta_{fin}=\pi\quad\textrm{and}\quad x_{fin}=0\quad \textrm{and}\quad y_{fin} \geq0, \end{aligned}$$
  where *E*(*z*,*m*) is given by Eq. (56).
6. The critical surface splits the range of the exponential map into four disjoint parts, cf. Fig. 11. These parts $\mathcal{C}_{1}^{1}$, $\mathcal{C}^{0}_{1}$, $\mathcal{C}_{2}^{+}$ and $\mathcal{C}_{2}^{-}$ directly relate to the splitting of the phase space, into the four parts $C_{1}^{1}$, $C^{0}_{1}$, $C_{2}^{+}$ and $C_{2}^{-}$.
7. If $g_{fin}=(x_{fin},y_{fin},\theta_{fin}) \in\mathcal{R}$ then $g_{fin}=(x_{fin},y_{fin}, \theta_{fin}+\pi) \notin\mathcal{R}$.

Let’s underpin these observations with theorems.

#### Lemma 5

*Let* 0<*a*<*b*<1. *Then* $\varPsi(a,b):=\frac{a}{\sqrt{1+b}} -\frac{1}{2} \log( \frac{b+a}{b-a} )<0$.

#### Proof

*Ψ* does not contain stationary points in the open region in $\mathbb{R} ^{2}$ given by 0<*a*<*b*<1. At the boundary we have *Ψ*(0,*b*)=0 and lim_*b*↓*a*_ *Ψ*(*a*,*b*)=−∞ and $\varPsi(a,1)= \frac{a}{\sqrt{2}}-\frac{1}{2} \log( \frac {1+a}{1-a} )$ and $\frac{\partial\varPsi(a,1)}{\partial a}<0$ so *Ψ*(*a*,*b*)<*Ψ*(0,1)=0 for 0<*a*<*b*<1. □

#### Theorem 7

*The range* $\mathcal{R}$ *of the Exponential map of* **P**_curve_ *is contained within the half space* *x*≥0. *In particular*, *its boundary* $\partial\mathcal{R}$ (*i*.*e*. *the cusp*-*surface*) *is contained within* *x*≥0.

#### Proof

From Theorem 3 we deduce that

58 $$\begin{aligned} x_{fin} =&x(\ell)=\frac{(z(\ell)-z_0)\dot{z}_{0}}{\mathfrak{c}^2} \\ &{}+ \frac{\sqrt{1-|z_0|^2}}{\mathfrak{c}^2} \int_{0}^{\ell} \sqrt{1-(z(s))^2}\, {\rm d}s. \end{aligned}$$

One has (see Fig. 9)

$$\bigl(z(\ell)-z_0\bigr) \dot{z}_{0} \leq0 \quad\textrm{iff}\quad -z_0 \leq\dot{z}_{0} \leq0 \quad\textrm{or}\quad z_0 \geq\dot{z}_{0} \geq0. $$

In the other cases in the phase portrait where

$$\begin{aligned} \frac{(z(\ell )-z_0)}{\dot{z}_{0}} \geq0 \end{aligned}$$

the result is obvious. Via symmetry considerations one only needs to consider the case

$$- z_{0}\leq\dot{z}_{0}\leq0, $$

where *z*(*s*_*max*_)=1. Then we apply Lemma 5 (with $a=-\dot{z}_{0}$ and *b*=*z*_0_) from which we deduce that

59 $$ \frac{-\dot{z}_0}{\sqrt{1+|z_0|}}< \frac{1}{2} \log\biggl( \frac{z_0- \dot{z}_{0}}{z_0+ \dot{z}_{0}} \biggr). $$

In the remainder of this proof we will show that

60 $$\begin{aligned}& \int_{0}^{\ell} \sqrt{1-|z(s)|^2} {\rm d}s \geq\frac {-\dot{z}_0\sqrt{1-z_0}}{ \sqrt{1+|z_0|}} \\& \phantom{\int_{0}^{\ell} \sqrt{1-|z(s)|^2} {\rm d}s}= \frac{(1-z_0)|\dot{z}_0|\mathfrak{c}^2}{\mathfrak{c}^2\sqrt{1-|z_0|^2}} \geq\frac{(z(\ell)-z_0)|\dot{z}_0|\mathfrak{c}^2}{\mathfrak{c}^2 \sqrt{1-|z_0|^2}} , \end{aligned}$$

which yields the result *x*_*fin*_≥0. In order to show Eq. (60) we consider the integrand $\psi(s):= \sqrt{1-|z(s)|^{2}}$ which is a continuous (concave) function with a single maximum at *s* ^∗^ with $\dot{z}(s^{*})=0$ which yields (under the condition $-z_{0}\leq\dot{z}_{0} \leq0$)

$$s^{*}=\frac{1}{2} \log\biggl(\frac{z_0- \dot{z}_{0}}{z_0+\dot {z}_{0}} \biggr) $$

so that indeed by means of Eq. (59), see Fig. 18

$$\begin{aligned} \int_{0}^{\ell} \sqrt{1-|z(s)|^2} {\rm d}s \geq&\sqrt{1-|z_0|^2} s^{*} \geq\sqrt{1-|z_0|} s^{*} \\ \geq& \frac{-\dot{z}_0\sqrt{1-z_0}}{ \sqrt{1+|z_0|}}, \end{aligned}$$

from which the final result *x*(*ℓ*)=*x*_*fin*_≥0 follows by Eq. (58) and Eq. (60). □

For analysis of $\mathcal{R}$ and $\partial\mathcal{R}$ and for (semi-)analytically solving of the boundary value problem the following identities (due to Theorem 3) come at hand.

#### Lemma 6

*We have the following relation between the momentum at* *s*=0

$$p_{0}= z_{0}\omega^{1} + \sqrt{1-|z_0|^{2}} \omega^{2} + \dot{z}_{0}\omega^{3} $$

*and the end*-*condition* *g*_*fin*_=(*x*_*fin*_,*y*_*fin*_,*θ*_*fin*_):

61 $$ \begin{aligned} &(\dot{z}_{0})^2 -(z_{0})^{2} = (\dot{z}(\ell))^2 -(z(\ell))^{2}, \\ &\dot{z}(\ell)= \dot{z}_{0} \cos(\theta_{fin}) + \sqrt {1-|z_0|^{2}} \sin(\theta_{fin}), \\ &z(\ell)= z_0 + x_{fin} \dot{z}_0 + y_{fin} \sqrt{1-|z_0|^2}. \end{aligned} $$

*This yields a quadratic polynomial equation in* $\dot{z}_{0}$:

62 $$ \begin{aligned} &a \dot{z}_{0}^{2} + b \dot{z}_{0} +c = 0 \textrm{ with }\\ &a= (x_{fin})^{2} + \sin^{2}(\theta_{fin}), \\ &b= 2 x_{fin}(z_0+y_{fin} \sqrt{1-|z_0|^2}) \\ &\phantom{b=}{}- \sqrt{1-|z_{0}|^2} \sin(2 \theta_{fin}), \\ &c= (|z_0|^2 -1) \sin^{2}(\theta_{fin}) \\ &\phantom{c=}{}+ y_{fin}^{2} (1-|z_0|^2)+ 2 y_{fin} z_0 \sqrt{1-|z_0|^{2}} , \end{aligned} $$

*the discriminant* *D*=*b* ^2^−4*ac*≥0 *equals*

63 $$ \begin{aligned} &D=2(\alpha+ R_{1} \cos(2\theta_{fin})+R_{2} \sin(2\theta_{fin}))\\ &\phantom{D}= 2(\alpha+\rho\cos(2\theta_{fin}-\psi))\quad \textrm{with} \\ &\quad R_{1}= (1-|z_0|^2)(y_{fin}^{2}-x_{fin}^{2}-1)\\ &\phantom{\quad R_{1}=}{}+ 2y_{fin} z_0 \sqrt {1-|z_0|^{2}}, \\ & \quad R_{2}= -(1-|z_0|^2)(2 x_{fin} y_{fin})\\ & \phantom{\quad R_{2}=}{}- 2x_{fin} z_0 \sqrt {1-|z_0|^{2}}, \\ & \quad\alpha= -R_{1} + 2|x_{fin}|^{2}|z_{0}|^2, \\ & \quad\rho=\sqrt{R_{1}^{2}+R_{2}^{2}},\quad \psi= \arg\biggl( \frac{R_{1}+i R_{2}}{\rho} \biggr), \end{aligned} $$

*and whose solutions are expressed in* *z*_0_ *via*

64 $$ \dot{z}_{0}=\frac{-b \pm\sqrt{D}}{2a}. $$

#### Theorem 8

*In* **P**_curve_ *the plane* *x*_*fin*_=0 *is only reached by a non*-*trivial geodesic that starts in a cusp and ends in a cusp with angle* *θ*_*fin*_=*π*, *i*.*e*.

$$\begin{aligned}& (x_{fin}=0 \textit{ and }y_{fin}\neq0) \\& \quad\Leftrightarrow\quad|\theta_{fin}|=\pi\\& \quad\Leftrightarrow\quad(|z_{0}|=|z(\ell)|=1 \textit{ and }\dot{z}_{0}=-\dot {z}(\ell)) \end{aligned}$$

#### Proof

Suppose |*θ*_*fin*_|=*π* then on the one hand by Eq. (61) we have $\dot{z}(\ell)=-\dot{z}_{0}$ whereas on the other hand by Eq. (52) we have $\dot{z}_{\ell}\sqrt {1-|z_{0}|^{2}}-\dot{z}_{0}\sqrt{1-|z(\ell)|^{2}}=0$ from which we deduce |*z*(*ℓ*)|=|*z*_0_|=1. Suppose |*z*_0_|^2^=|*z*(*ℓ*)|^2^=1 and $\dot{z}_{0}=-\dot{z}(\ell)$ then *z*(0)≠−*z*(*ℓ*) and we obtain *x*_*fin*_=0 and *y*_*fin*_≠0 by Eq. (52). Finally, suppose *x*_*fin*_=0 and *y*_*fin*_≠0 then *D*=*ψ*=*R*_2_=0 and *ρ*=*R*_1_=−*α* in Eq. (63) and thereby we obtain cos(2*θ*_*fin*_)=1 and the result follows □

**Fig. 13:**
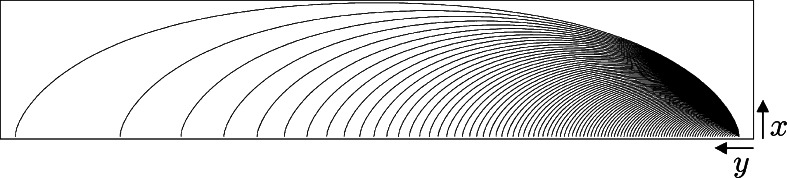
For points on the cusp-surface one has *z*_0_=*z*(*ℓ*)⇔*x*_*fin*_=0⇔*θ*=±*π*. We have depicted the geodesics (with maximal length until cusp) of the 1-control problem where we set *z*_0_=1 while varying $\dot{z}_{0} \in [-1,0]$

See Fig. 13 for an illustration of such geodesics.

### The Cones of Reachable Angles

We will provide a formal theorem that underpins our observations of the cone of reachable angles *θ*_*fin*_ per end-position (*x*_*fin*_,*y*_*fin*_), recall (57). Recall that *θ*_endcusp_(*x*_*fin*_,*y*_*fin*_) denotes the final angle of the geodesic ending in (*x*_*fin*_,*y*_*fin*_,⋅) with a cusp and where *θ*_begincusp_(*x*_*fin*_,*y*_*fin*_) denotes the final angle of a geodesic ending in (*x*_*fin*_,*y*_*fin*_,⋅) starting with a cusp. In case there exist two geodesics ending with a cusp at (*x*_*fin*_,*y*_*fin*_) we order their end-angles by writing

$$\theta_{\mathrm{endcusp}}^{1}(x_{fin},y_{fin})\leq \theta_{\mathrm {endcusp}}^{2}(x_{fin},y_{fin}). $$

#### Theorem 9

*Let* $(x_{fin},y_{fin},\theta_{fin}) \in\mathcal{R}$. *If*

65 $$ \begin{aligned} &|y_{fin}|\leq-x_{fin} i E \biggl(i \,\mathrm{arcsinh}\, \frac {x_{fin}}{\sqrt{4-x_{fin}^2}}, \frac{x_{fin}^2-4}{x_{fin}^2} \biggr),\quad\textit{and} \\ &0\leq x_{fin}<2. \end{aligned} $$

*then we have*

$$\begin{aligned}& y_{fin}>0 \quad\Rightarrow\\& \quad\theta_{fin} \in [ \theta_{\mathrm{begincusp}}(x_{fin},y_{fin}), \theta_{\mathrm {endcusp}}(x_{fin},y_{fin})], \\& y_{fin}<0\quad \Rightarrow\\& \quad\theta_{fin} \in [ \theta_{\mathrm{endcusp}}(x_{fin},y_{fin}), \theta_{\mathrm {begincusp}}(x_{fin},y_{fin})], \end{aligned}$$

*otherwise* (*so in particular if* *x*_*fin*_≥2) *we have*

$$\theta_{fin} \in\bigl[\theta_{\mathrm{endcusp}}^{1}(x_{fin},y_{fin}), \theta_{\mathrm{endcusp}}^{2}(x_{fin},y_{fin})\bigr]. $$

For a direct graphical validation of Theorem 9 see Fig. 11 (in particular the top view along *θ*), where we note that the bound in (65) relates to the spatial projection of the curve that arises by taking the intersection of the blue and red surface on $\partial\mathcal{R}$ at *θ*=0 (the thick black line in Fig. 11 at *θ*=0). For more details on the proof see Appendix E.

As already mentioned in Sect. 3.1, it does not matter if one considers problem **P**_curve_ on the projective line bundle $\mathbb{R}^{2} \rtimes P^{1}$ or on $\mathbb{R}^{2} \rtimes S^{1} \equiv\mathrm {SE}(2)$. This is due to the following theorem.

#### Theorem 10

*If*

$$\begin{aligned} (x_{fin},y_{fin},\theta_{fin}) \in\mathcal{R}, \end{aligned}$$

*then*

$$\begin{aligned} (x_{fin},y_{fin},\theta_{fin}+\pi) \notin\mathcal{R}. \end{aligned}$$

#### Proof

From Theorem 3 we have $-\dot {\tilde{y}}(s) \geq0$ from which we deduce condition $\sin(\theta _{fin}-\overline{\theta}_{0}) \leq0$ implying the result. □

## Solving the Boundary Value Problem

In order to explicitly solve the boundary-value problem for **P**_curve_ for admissible boundary conditions (Eq. (3)) we can apply left-invariance (i.e. rotation and translation invariance) of the problem and consider the case *g*_*in*_=*e*=(0,0,0) and $g_{fin} \in \mathcal{R}$.

Recall from Eq. (20) that initial momentum *p*_0_ is determined by *z*_0_ and $\dot{z}_{0}$:

$$p_0= z_{0} \omega^{1} + \sqrt{1-|z_0|^{2}} \omega^{2} + -\dot{z}_{0} \omega^{3}. $$

Now solving the boundary value problem boils down to expressing (*p*_0_,*ℓ*) directly into

$$\begin{aligned} g_{fin}=(x_{fin},y_{fin},\theta _{fin}), \end{aligned}$$

since when we achieve to do so we have

66 $$ \widetilde{\mathrm{Exp}}\bigl(p_{0}(g_{fin}), \ell(g_{fin})\bigr)= g_{fin}, $$

and the globally minimizing curve of **P**_curve_ is given by

$$\begin{aligned} s \mapsto\gamma(s):=\widetilde{\mathrm{Exp}}(p_{0}(g_{fin}),s). \end{aligned}$$

In fact, this means we must find the inverse of the exponential map $\widetilde{\mathrm{Exp}}$. The inverse of this exponential map exists due to Theorems 6, 4 and 1.

We invert the boundary value problem for a very large part analytically, yielding a novel very fast and highly accurate algorithm to solve the boundary value problem. In comparison to previous work on this topic [45], we have less parameters to solve (and moreover, our proposed optimization algorithm involves less parameters).

First of all we directly deduce from Theorem 3, Lemma 6 and Eq. (40) that

67 $$ \begin{aligned} &e^{\ell}= \left\{ \begin{array}{l@{\quad}l} \frac{z_0}{v}, & \mathfrak{c}=1, \dot{z}_{0}=- z_{0}, \\ \frac{v}{z_0}, & \mathfrak{c}=1, \dot{z}_{0}= z_{0}, \\ \frac{v+w}{z_0+ \dot{z}_{0}}, & \textrm{ else }\\ \end{array} \right. \\ &e^{\ell} \leq e^{s_{\mathrm{max}}}:= \frac{1+\mathfrak{c}}{|z_0+ \dot {z}_{0}|}, \end{aligned} $$

where $v,w,\mathfrak{c}$ are given by

68 $$ \begin{aligned} &v=z(\ell)=z_0 + x_{fin} \dot{z}_{0} + y_{fin} \sqrt{1-|z_0|^{2}}, \\ &w=\dot{z}(\ell)= \dot{z}_{0} \cos\theta_{fin} + \sqrt {1-|z_0|^{2}} \sin\theta_{fin},\\ &\mathfrak{c}=\sqrt{1- |z_{0}|^2 + |\dot{z}_{0}|^2}. \end{aligned} $$

Now we have already expressed two of the three unknowns in the end condition

69 $$ \begin{aligned} &\ell=\ell(z_0, \dot{z}_{0}, g_{fin})\quad \textrm{given by Eq.}~(67), \\ &\dot{z}_{0}=\dot{z}_{0}(z_0, g_{fin})\quad \textrm{given by Eq.}~(64). \end{aligned} $$

The remaining unknown variable *z*_0_∈[−1,1] can be found via a simple numerical algorithm to find the unique root of a function $F:I \to\mathbb{R}^{+}$, where *I*⊂[−1,1] is a known and determined by *g*_*fin*_.

However, before we can formulate this formally there is a technical issue to be solved first, which is the choice of sign in Eq. (64).

### Lemma 7

*Let surface* $\mathcal{V} \subset\mathrm{SE}(2)$ *be given by*

70 $$ \begin{aligned} &\mathcal{V}= \bigl\{ \widetilde{\mathrm{Exp}}(z_0 \omega^{1} + \sqrt {1-|z_0|^{2}}\omega^{2}, \ell)\mid\\ &\quad\quad\ z_0 \in[-1,1] \textrm{ and }0\leq\ell\leq s_{max}(z_0,0) \bigr\} . \end{aligned} $$

(*where* $\dot{z}_{0}=0$). *Given* $g_{fin} \in\mathcal{R}$ *we have*

$$\dot{z}_{0}(z_0)= \frac{-b + {\rm sign}(g_{fin}) \sqrt{D}}{2a}, $$

*with* *a*=*a*(*g*_*fin*_,*z*_0_),*b*=*b*(*g*_*fin*_,*z*_0_) *given by Eq*. (62) *and* *D*=*D*(*g*_*fin*_,*z*_0_) *given by Eq*. (63) *and with* ${\rm sign}(g_{fin})$ *given by*

71 $$ \rm{sign}(g_{fin})= \left\{ \begin{array}{l@{\quad}l} 1 & \textrm{if }g_{fin} \in\mathcal{C}_{2}^{+},\\ 1 &\textrm{if } g_{fin} \in\mathcal{C}_{1}^{1} \cup\mathcal {C}_{1}^{0}\textrm{ is above }\mathcal{V}, \\ -1 &\textrm{if } g_{fin} \in\mathcal{C}_{1}^{1} \cup\mathcal {C}_{1}^{0} \textrm{ is below }\mathcal{V,} \\ -1 & \textrm{if }g_{fin} \in\mathcal{C}_{2}^{-}. \end{array} \right. $$

### Proof

The $\widetilde{\mathrm{Exp}}$ is a (global) homeomorphism and its orbits $s \mapsto\widetilde{\mathrm{Exp}}(p_{0},s)$ are analytic for each $p_{0} \in T^{*}_{e}(\mathrm{SE}(2))$. Thereby the sign cannot switch along orbits (unless *D*=0, which only occurs at *θ*_*fin*_=±*π* at $\partial\mathcal{R}$). Furthermore, since $\widetilde{\mathrm{Exp}}$ is a homeomorphism sign switches (in Eq. (64)) between neighboring orbits are not possible unless it happens across an orbit $s\mapsto(z(s),\dot {z}(s))$ with $\dot{z}_{0}=0$. Now from the phase portrait it is clear that orbits in phase space $s \mapsto(z(s), \dot{z}(s))$ with $\dot{z}(s)>0$ and $\mathfrak{c}>1$, i.e. orbits in $C^{+}_{2}$ need a plus sign, whereas orbits in $C^{-}_{2}$ need a minus sign in Eq. (64). The line $\dot{z}_{0}=0$ splits the phase portrait in two parts, and by the results in Theorem 6 this means that the surface $\mathcal{V}$ splits the set $\mathcal{R}$ into two parts. Now $\widetilde{\mathrm{Exp}}$ maps $C^{+}_{2}$ onto $\mathcal{C}^{+}_{2}$ and it maps $C^{-}_{2}$ onto $\mathcal{C}^{-}_{2}$, and $\mathcal{C}^{-}_{2}$ lies beneath *V* and $\mathcal{C}^{+}_{2}$ lies above *V*, from which the result follows. □

### Remark 7.1

The surface $\mathcal{V}$ is depicted in Fig. 15. Lemma 7 is depicted in Fig. 16, where we used Theorem 3 to compute for each point in $(z_{0},\dot{z}_{0}) \in[-1,1] \times[-2,2]$ in phase space the sign of $2a \dot{z}_{0}+b$ at respectively $s=0, \frac{1}{2}s_{max}(z_{0},0), \frac{3}{4}s_{max}(z_{0},0)$ and *s*=*s*_*max*_(*z*_0_,0). We see that the black points (where the sign is positive) lies above the orbits family of orbits with *z*_0_∈[−1,1] and $\dot{z}_{0}=0$.

### Remark 7.2

The explicit parametrization for plane $\mathcal {V}$ is given by the union of the *x*-axis and the surface parameterized by

$$\left\{ \begin{array}{l} x(\ell,z_0)= -i \sqrt{1-|z_0|^{2}} E (i \ell, \frac {|z_0|^{2}}{|z_0|^{2}-1} ),\\ y(\ell,z_0)= \frac{z_0}{\sqrt{1-|z_0|^2}} (\cos h \ell-1),\\ \theta(\ell,z_0)= \arctan( \frac{z_0 \sinh\ell}{\sqrt {1-|z_0\cosh\ell|^2}} ), \end{array} \right. $$

*z*_0_∈(−1,1)∖{0}, 0≤*ℓ*≤arccosh(|*z*_0_|^−1^).

The next theorem reduces the boundary value problem to finding the unique root of a single positive real-valued function.

### Theorem 11

*Let* $g_{fin} \in\mathcal{R}$. *The inverse of the exponential map in Definition *2 *is given by*

$$\begin{aligned} &p_{0}= \sum_{i=1}^{2}\lambda_{i}(0) \omega^{i}, \\ &\ell= \ell(z_0,\dot{z}_0,x_{fin},y_{fin},\theta_{fin})\quad \textit{ given by Eq.}~(67) \end{aligned} $$

*with* *λ*_1_(0)=*z*_0_, $\lambda_{2}(0)=\sqrt{1-|z_{0}|^{2}}$, $\lambda_{3}(0)=-\dot{z}_{0}$, *where* $\dot{z}_{0}(z_{0},g_{fin})$ *given in Lemma *7 *and with discriminant* *D*(*z*_0_,*g*_*fin*_) *given by Eq*. (63) *and where* *z*_0_ *denotes the unique zero* *F*(*z*_0_)=0 *of function* $F:I \to \mathbb{R}^{+}$ *defined on*

$$\begin{array}{l} I=\{z_{0} \in[-1,1]\mid D(z_0,x_{fin},y_{fin},\theta_{fin}) \geq0 \} \end{array} $$

*given by*

$$\begin{aligned} &F(z_0) = \|\widetilde{\mathrm{Exp}}(z_{0}\omega^{1}+ \sqrt{1-|z_0|^2} \omega^{2} \\&\phantom{F(z_0) =}{}- \dot{z}_{0}(z_0,g_{fin}) \omega^{3} \ell(z_0,g_{fin}) ) - g_{fin}\| \end{aligned} $$

*where* ∥⋅∥ *denotes the Euclidean norm on* $\mathbb{R}^{2}\times S^{1}$.

### Proof

By Theorem 1 there is a unique stationary curve connecting *e* and $g_{fin} \in\mathcal{R}$. The exponential map of **P**_curve_ is a homeomorphism by Theorem 6 and thereby the continuous function *F* has a unique zero, since *ℓ* and $\dot{z}_{0}$ are already determined by *z*_0_ and *g*_*fin*_ via Theorem 3 and Lemma 7. □

### Remark 7.3

Theorem 11 allows fast and accurate computations of sub-Riemannian geodesics, see Fig. 12 where the computed geodesics are instantly computed with an accuracy of relative $\mathbb{L}_{2}$-errors in the order of 10^−8^. Finally, we note that Theorem 6 implies that (our approach to) solving the boundary-value problem is well-posed (i.e. the solutions are both unique and stable).

## Modeling Association Fields with Solutions of **P**_curve_

Contact geometry plays a major role in the functional architecture of the primary visual cortex (V1) and more precisely in its pinwheel structure, cf. [52]. In his paper [52] Petitot shows that the horizontal cortico-cortical connections of V1 implement the contact structure of a continuous fibration *π*:*R*×*P*→*P* with base space the space of the retina and *P* the projective line of orientations in the plane. This model was refined by Citti and Sarti [22], who formulated the model as a contact structure within SE(2) producing problem **P**_curve_ given by Eq. (11).

Petitot applied his model to the Field’s, Hayes’ and Hess’ physical concept of an association field, to several models of visual hallucinations [32] and to a variational model of curved modal illusory contours [42, 48, 65].

In their paper, Field, Hayes and Hess [34] present physiological speculations concerning the implementation of the association field via horizontal connections. They have been confirmed by Jean Lorenceau et al. [43] via the method of apparent speed of fast sequences where the apparent velocity is overestimated when the successive elements are aligned in the direction of the motion path and underestimated when the motion is orthogonal to the orientation of the elements. They have also been confirmed by electrophysiological methods measuring the velocity of propagation of horizontal activation [37].

There exist several other interesting low-level vision models and psychophysical measurements that have produced similar fields of association and perceptual grouping [39, 49, 68], for an overview see [52, Chaps. 5.5, 5.6].

### Three Models and Their Relation

Subsequently, we discuss three models of the association fields: horizontal exponential curves, Legendrian geodesics, and cuspless sub-Riemannian geodesics (which for many boundary conditions coincide with Petitot’s circle bundle model, as we will explain below).

With respect to the first model we recall that horizontal exponential curves [26, 57] in the sub-Riemannian manifold $(\mathrm{SE}(2),\Delta,\mathcal {G}_{\xi})$, recall Eq. (17), are given by circular spirals

72 $$\begin{aligned} & r \mapsto g_0 e^{r(c^{1}A_{1}+c^{2}A_{2})} \\ &\quad =\biggl(x_0 + \frac{c^{2}}{c^{1}}(\sin(c^{1}r + \theta_0)-\sin (\theta_{0})), \\ &\quad\quad\ y_0-\frac{c^{2}}{c^{1}}(\cos(c^{1}r + \theta_0)-\cos (\theta_{0})) ,\theta_{0} + r c^{1} \biggr), \end{aligned}$$

for *c* ^1^≠0, *g*_0_=(*x*_0_,*y*_0_,*θ*_0_)∈SE(2) and all *r*≥0. If *c* ^1^=0 they are straight lines:

$$g_0 e^{rc^{2}A_{2}}=\bigl(x_0+ r c^{2}\cos \theta_0, y_0+r c^{2} \sin \theta_{0},\theta_{0}\bigr). $$

Clearly, these horizontal exponential curves reflect the co-circularity model [46].

To model the association fields from psychophysics and neurophysiology Petitot [52] computes “Legendrian geodesics”, [52, Chap. 6.6.4, Eq. (49)] minimizing Lagrangian $\sqrt{1+ |y'(x)|^{2}+ |\theta'(x)|^{2}}$ under the constraint *θ*(*x*)=*y*′(*x*). This is directly related^11^ to the sub-Riemannian geodesics in

73 $$ \bigl(\bigl(\mathrm{SE}(2)\bigr)_{0}, \mathrm {Ker}(- \theta\, {\rm d}x+ {\rm d}y), {\rm d}\theta\otimes{\rm d}\theta+{\rm d}x \otimes{\rm d}x \bigr), $$

where (SE(2))_0_ is the well-known nilpotent Heisenberg approximation [25, Chap. 5.4]) of SE(2), which minimize Lagrangian $\sqrt{1+ |\theta'(x)|^{2}}$ under constraint *θ*(*x*)=*y*′(*x*). The drawback of such curves is that they are coordinate dependent and not covariant^12^ with rotations and translations. Similar problems arise with B-splines which minimize Lagrangian 1+|*θ*′(*x*)|^2^ under constraint *θ*(*x*)=*y*′(*x*) which are commonly used in vector graphics.

To this end Petitot [52] also proposed the “circle bundle model” which has the advantage that it is coordinate independent. Its energy integral

$$\int_{0}^{x_{fin}}\sqrt{1+ |y'(x)|^2 + \frac {|y''(x)|^2}{(1+|y'(x)|^2)^2}} {\rm d}x $$

can be expressed as $\int_{0}^{\ell} \sqrt{1+\kappa^{2}} {\rm d}s$, where *s*∈[0,*ℓ*] denotes spatial arclength-parametrization. As long as the curve can be well-parameterized by *x*↦(*x*,*y*(*x*),*θ*(*x*)) this model coincides^13^ with sub-Riemannian geodesics.

For the explicit connections between each of the 3 mathematical models we refer to Appendix G.

### Sub-Riemannian Geodesics Versus Co-circularity

In Fig. 8 we have modeled the association field with sub-Riemannian geodesics (*ξ*=1) and horizontal exponential curves (Eq. (72) as proposed in [9, 57]). Horizontal exponential curves are circular spirals and thereby rely on “co-circularity”, a well-known basic principle to include orientation context in image analysis, cf. [35, 46].

On the one hand, a serious drawback arising in the co-circularity model for association fields is that the only the spatial part (*x*_*fin*_,*y*_*fin*_) of the end-condition can be prescribed (the angular part is imposed by co-circularity), whereas with geodesics one can prescribe (*x*_*fin*_,*y*_*fin*_,*θ*_*fin*_) (as long as the ending condition is contained within $\mathcal{R}$). This drawback is clearly visible in Fig. 8, where the association field (see a) in Fig. 8) typically ends in points with almost vertical tangent vectors.

On the other hand, the sub-Riemannian geodesic model has more difficulty describing the association field by Field and co-workers in the almost circular connections to the side (where the co-circularity model is reasonable). To this end we note that circles are not sub-Riemannian geodesics as the ODE $\ddot{z}=\xi z$ does not allow *z* to be constant.

This difficulty, however, can be tackled by variation of *ξ* in Problem **P**_curve_. Our algorithm explained in Sect. 5, combined with the scaling homothety described in Remark 1.2, is well-capable of reconstructing the almost circular field line cases as well. This can be observed in Fig. 17.

### Variation of *ξ* and Association Field Modeling

**Fig. 14:**
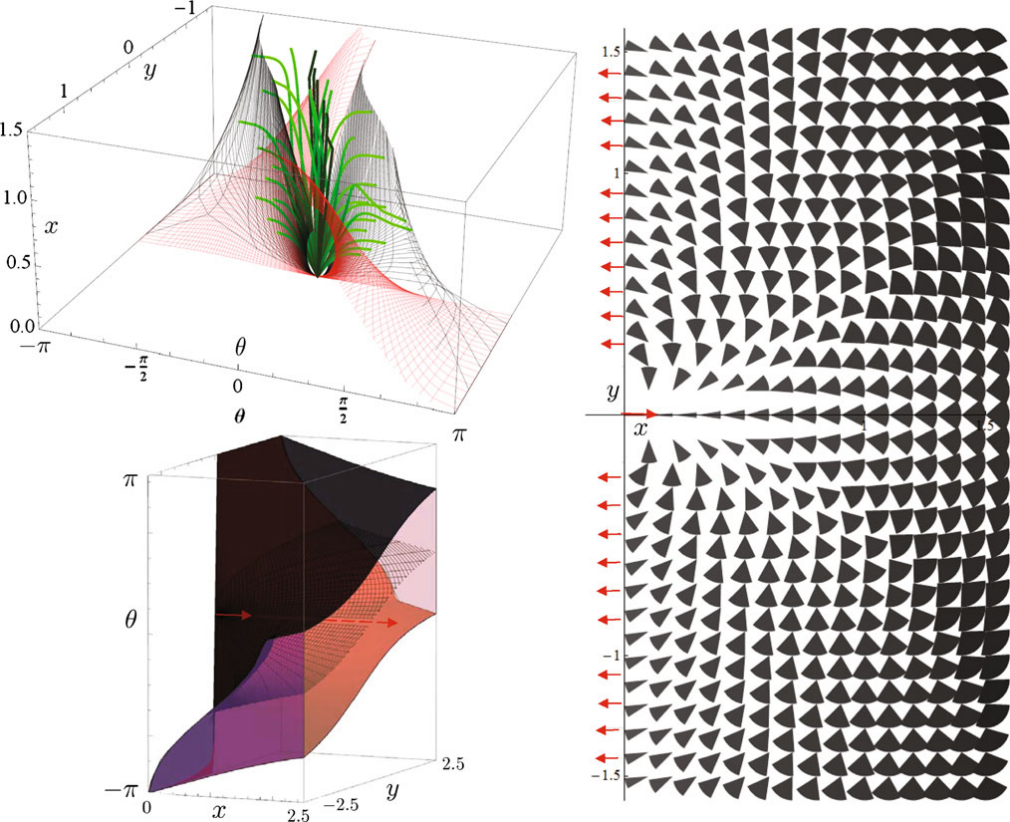
*Left; top*: the range of the exponential map for *ξ*=1. Within this range we have plotted several sub-Riemannian geodesics. The boundary of the range of the exponential map contains a *black surface* and a *red surface*, the *black surface* denotes points with cusps at the end (*green* geodesics end here), the *red surface* denotes points with cusps in the origin. *Bottom*: the range of the exponential map depicted as a volume in [0,2.5]×[−2.5,2.5]×*S* ^1^, within this volume we have plotted the critical curve surface (spanned by the solutions with *s*_max_=∞). *Right*; the field of reachable cones, determined by the tangent vector of a sub-Riemannian geodesic with a cusp at the end-point (*x*,*y*,*θ*_max_) and the tangent vector of a sub-Riemannian geodesic with a cusp at the origin (0,0,0) ending at (*x*,*y*,*θ*_min_). The range of the exponential map is contained within *x*≥0, cf. Theorem 7 and *x*=*x*_*fin*_=0 is reached with angle *θ*_*fin*_=*π*≡−*π*, cf. Theorem 8 (Color figure online)

See Fig. 17 to see the effect of *ξ*>0 on the modeling of association fields. The larger *ξ* the shorter the spatial part of the paths, and the more bending we see in the vicinity of the end-points. The smaller the *ξ* the more circular the shape becomes at the sides of the association field model. Here we note that for these smaller values of *ξ*, the end-points of the more straight association field lines become problematic. In Fig. 17 one can see that when choosing *ξ* too small the end-point of the most straight field line even lies outside the range $\mathcal{R}$ of the exponential map. This effect is due to the fact that the boundary $\partial\mathcal{R}$ of the range of the exponential map, depicted in Figs. 11 and 14, scales with *ξ*>0 in spatial direction.

**Fig. 15:**
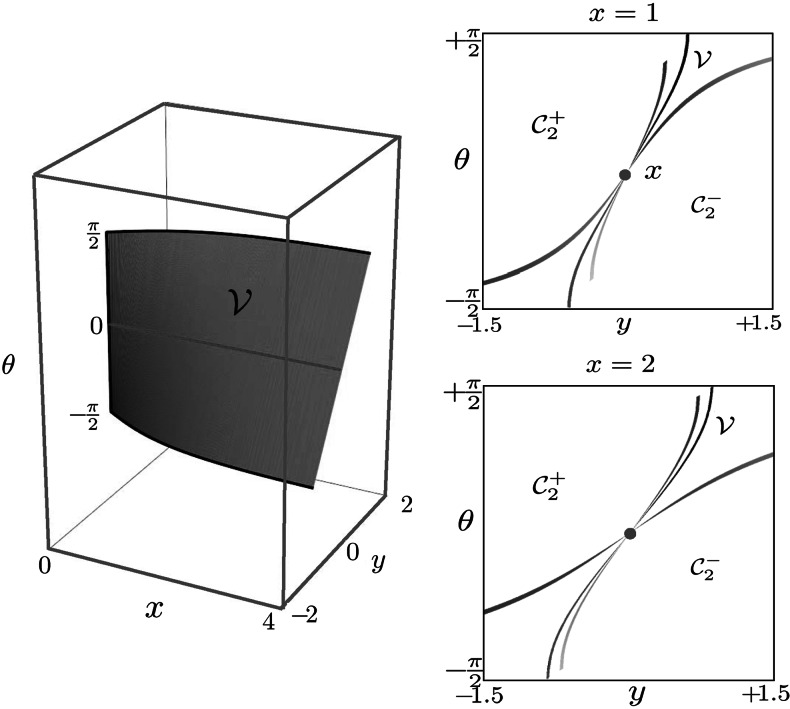
*Left*: the surface $\mathcal{V} \subset\mathrm {SE}(2)$ splits $\mathcal{R}$ into to parts and intersects the cusp surface $\partial\mathcal{R}$ at $\theta= \frac{\pi}{2}$ and $\theta=-\frac{\pi}{2}$. The *upper part* requires positive sign in the formula for $\dot{z}_{0}$ whereas the lower part requires a negative sign. *Right*: cross sections for *x*=1 and *x*=2 where $\mathcal{V}$ is contained within $\mathcal{C}_{1}^{1}$ and $\mathcal {C}_{1}^{0}$ and splits $\mathcal{C}_{2}^{+}$ and $\mathcal {C}_{2}^{-}$, cf. Fig. 11

**Fig. 16:**
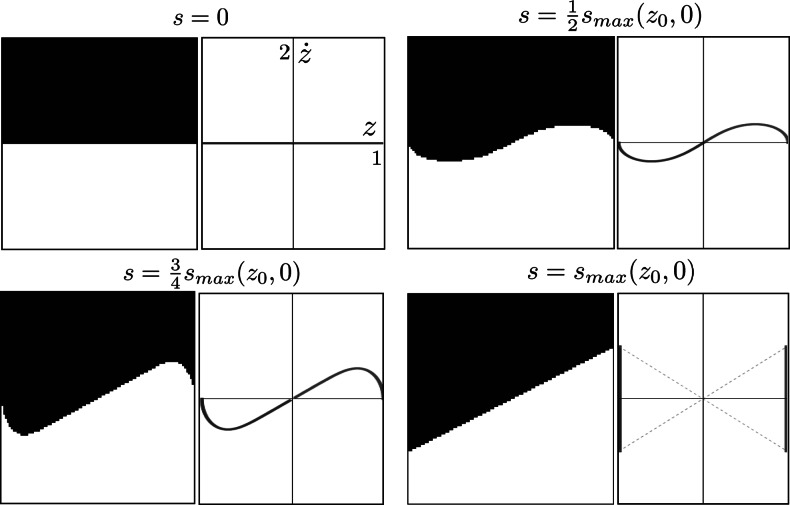
Points in the phase plane where the sign in Eq. (64) is + are depicted in black, whereas points in the phase plane where the sign in Eq. (64) is − are depicted in white. Next to these plots we provide the family of orbits [−1,1]∋*z*_0_↦(*z*_0_cosh(*s*),*z*_0_sinh(*s*)) (with $\dot{z}_{0}=0$) evaluated at $s=0, \frac{1}{2}s_{max}(z_{0},0), \frac{3}{4}s_{max}(z_{0},0)$ and *s*=*s*_*max*_(*z*_0_,0), to illustrate the idea behind Lemma 7

Varying of *ξ* ^2^>0 also takes into account a well-known parameter in completion; namely the area of the completed figures (see e.g. [52]). This area equals *A*=(*x*_*fin*_−*x*_*in*_)(*y*_*fin*_−*y*_*in*_). By Remark 1.1 we can as well set *x*_*in*_=*y*_*in*_=*θ*_*in*_=0 and then as explained in Remark 1.2 solving **P**_curve_ with *ξ*>0 amounts to solving **P**_curve_ with *ξ*=1 with scaled end-conditions (*x*_*fin*_ *ξ*,*y*_*fin*_ *ξ*). In fact, such rescaling of end-conditions rescales the area as follows *A*↦*Aξ* ^2^.

### A Conjecture and Its Motivation

**Fig. 17:**
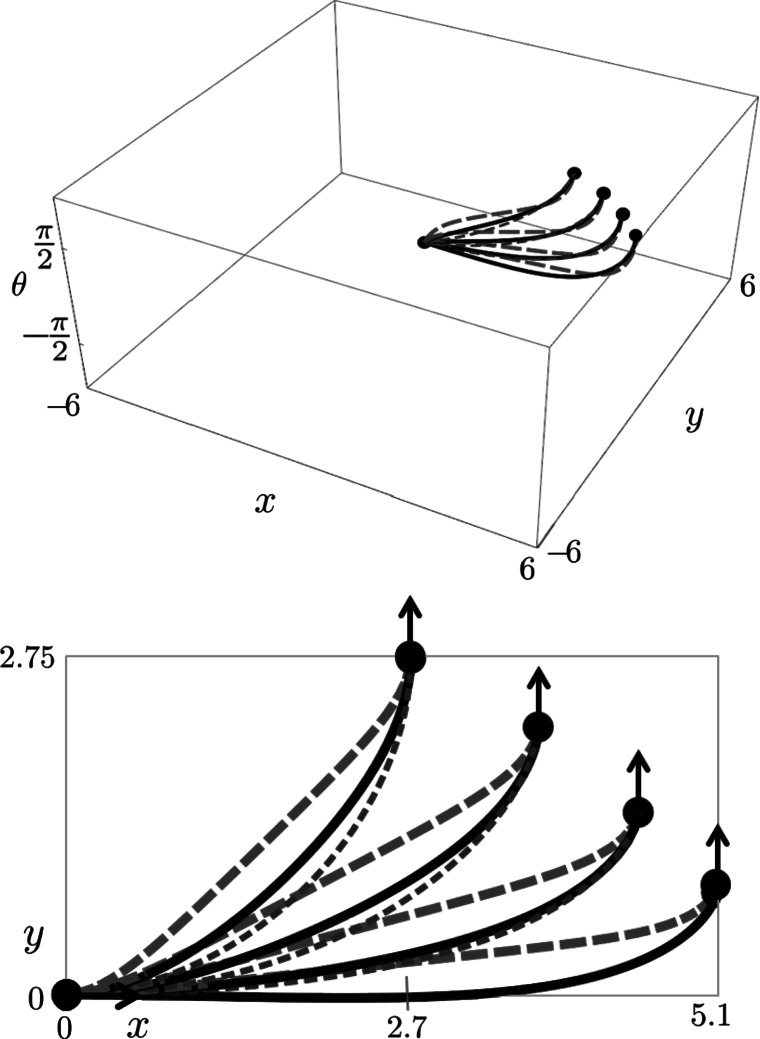
Sub-Riemannian geodesics in $(\mathrm{SE}(2),\Delta ,\mathcal{G}_{\xi })$ (*top*) and their spatial projections (*below*) with endpoints similar to the association field in Fig. 8(a) for various values of *ξ*>0, computed via the algorithm in Sect. 7 and Remark 1.2. *Black lines* are sub-Riemannian geodesics with *ξ*=1, the *dashed lines* in *red* are sub-Riemannian geodesics with *ξ*=3, and the *dashed lines* in *blue* are sub-Riemannian geodesics with $\xi=\frac{1}{3}$ (Color figure online)

**Fig. 18:**
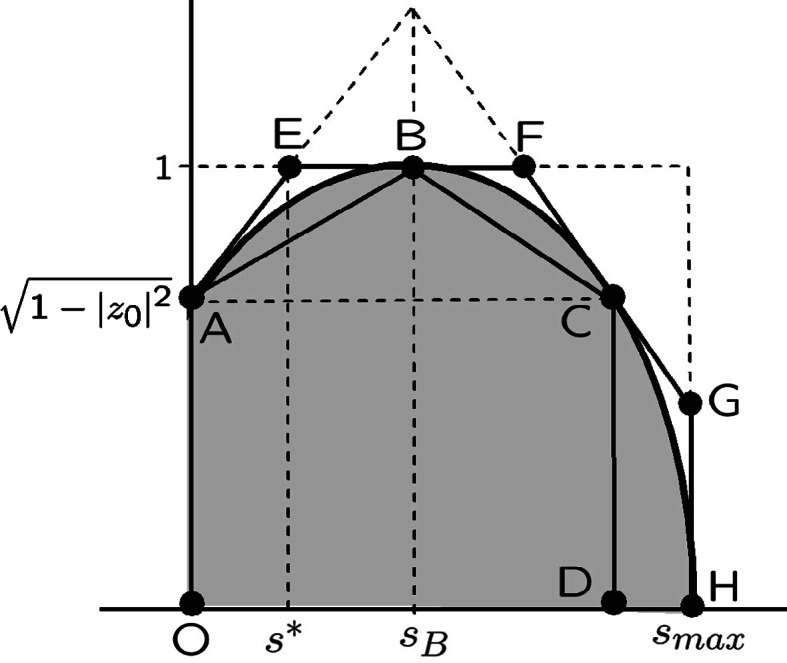
The graph of $s \mapsto\sqrt{1-|z(s)|^{2}}$ and the estimates for elliptic function $\int_{0}^{\ell} \sqrt{1-|z(s)|^{2}}{\rm d}s\leq\int_{0}^{s_{max}} \sqrt{1-|z(s)|^{2}}{\rm d}s$ for *c*<1 and $z_{0} \dot{z}_{0}<0$. The lower-bound is obtained by surface area *OABCD*. The upper-bound is obtained by surface area *OAEFGH*

The shape of the association field lines is well captured by the sub-Riemannian geodesics with *ξ*=1, in comparison to e.g. the exponential curves as can be observed in part b) of Fig. 8. See also Fig. 17. On top of that, the field curves of the association field end with vertical tangent vectors, and these end-points are very close to cusp points in the sub-Riemannian geodesics modeling these field lines. This can be observed both in Fig. 4 and in Fig. 17, where the sub-Riemannian geodesics ending at the end-points of the association field is nearly vertical. We will underpin this observation also mathematically in Lemma 8 and Remark 8.1.

Apparently, both the shape of the association field lines and their ending is well-expressed by the sub-Riemannian geodesics model **P**_curve_, which was proposed by Citti and Sarti [22]. Therefore, following the general idea of Petitot’s work [50] (in particular, his circle bundle model) and the results in this article on the existence set $\mathcal{R}$ this puts the following conjecture:

#### Conjecture 1

*The criterium in our visual system to connect two local orientations*, *say* *g*_0_=(*x*_0_,*y*_0_,*θ*_0_)=(0,0,0) *and* *g*_*fin*_=(*x*_*fin*_,*y*_*fin*_,*θ*_*fin*_)∈SE(2), *could be modeled by checking whether* *g*_*fin*_ *is within the range* $\mathcal{R}$ *of the exponential map*.

Here we recall that from the results in [16] (summarized in Theorem 1) it follows that the set $\mathcal{R}$ consists precisely of those points in SE(2) that are connected to the origin by a unique global minimizer of **P**_curve_. This conjecture needs further investigation by psycho-physical and neuro-physiological experiments. In any case, within the model **P**_curve_ (relating to Petitot’s circle bundle model [52] and the sub-Riemannian model by Citti and Sarti [22]) a curve is optimal if and only if it is stationary. Furthermore, the sub-Riemannian geodesics strongly deviate from horizontal exponential curves even if the end condition is chosen such that the co-circularity condition is satisfied (this can be observed in item c) of Fig. 8). This discrepancy between horizontal exponential curves and cusp-less sub-Riemannian geodesics in $(\mathrm{SE}(2), \Delta,\mathcal{G}_{\xi})$ is also intruiging from the differential geometrical viewpoint: see Theorem 12 in Appendix C.

In the remainder of this section we will mathematically underpin our observation that end-points of association fields are close to cusps.

#### Lemma 8

*Let* *γ* *be the sub*-*Riemannian geodesic with* *γ*(0)=(0,0,0) *and* *γ*(*ℓ*)=(*x*_*fin*_,*y*_*fin*_,*θ*_*fin*_)∈SE(2) *induced by the exponential map associated to the trajectory* $[0,\ell]\ni s \mapsto(z(s),\dot{z}(s))$ *with*

74 $$ 0\geq\dot{z}_{0}>-z_0 \quad\textit{and}\quad z( \ell)>0. $$

*Then for* $\dot{z}_{0}<0$ *small we have*

$$\theta_{fin}=\frac{\pi}{2} \quad\Rightarrow\quad e^{s_{\mathrm{max}}-\ell}= O\bigl(| \dot{z}_{0}|^2\bigr). $$

*Furthermore*, *under the conditions in Eq*. (74), *two of the following statements*

1. $\dot{z}_{0}=0$.
2. *γ* *ends with a cusp in* *γ*(*ℓ*)=(*x*(*ℓ*),*y*(*ℓ*),*θ*(*ℓ*)).
3. $|\theta(\ell)|=\frac{\pi}{2}$.

*imply the remaining third one*.

#### Proof

If $\theta_{fin}=\theta(\ell)=\frac{\pi}{2}$ then by Eq. (61) we have that $\dot{z}_{0}=-\sqrt{1-|z(\ell)|^{2}}$, so that

$$\begin{aligned} e^{s_{\mathrm{max}}-\ell} &= \frac{(1-\sqrt{1-|\dot {z}_{0}|^{2}})+(\mathfrak{c}- \sqrt{\mathfrak{c}^2-|\dot {z}_{0}|^{2}})}{|z_0+\dot{z}_{0}|} \\ &= O(|\dot{z}_{0}|^2) \end{aligned} $$

The rest follows by the fact that the second statement is equivalent to |*z*(*ℓ*)|=1 and the formula for *θ*_*fin*_ in Eq. (52). □

#### Remark 8.1

The curves in the association field have $\theta_{fin}=\frac{\pi }{2}$ and relatively small initial curvature so that $|\dot{z}_{0}|\ll 1$ and therefore they end very close to cusps, i.e. *ℓ*≈*s*_max_.

## Conclusion and Future Work

Under conditions (3) on the boundary conditions cuspless sub-Riemannian geodesics in

$$\bigl(\mathrm{SE}(2), \mathrm{span}\{\cos\theta\partial_{x}+\sin \theta\partial _{y}, \partial_{\theta}\}, \mathcal{G}_{\xi}\bigr) $$

coincide with the lifts of global minimizers of **P**_curve_ (i.e. curves optimizing $\int_{0}^{\ell}\sqrt{\kappa^{2}+ \xi^{2}} {\rm d}s$ with free length *ℓ* and given boundary conditions).

As the derivation of these cuspless geodesics is much less trivial than it seems (many conflicting results have appeared in the imaging literature on this topic), we derived them via 3 different mathematical approaches producing the same results from different perspectives. There are two ways to reasonably parameterize such curves, via spatial arclength and sub-Riemannian arclength and in this article we explicitly relate these parameterizations. The phase portrait in momentum space induced by sub-Riemannian arclength parametrization corresponds to (a strip within) the phase portrait of the mathematical pendulum, whereas the phase portrait in momentum space induced by spatial arclength parametrization is a hyperbolic phase portrait associated to a *linear* ODE for normalized curvature $z=\kappa /\sqrt{\kappa^{2}+\xi^{2}}$. Using the latter approach we have analyzed and computed the existence set $\mathcal{R}$ for **P**_curve_ (where every stationary curve is globally minimizing!). We have also solved the boundary value problem, where the numerics is reduced to finding the unique root of a continuous explicit real-valued function on a small subset of [−1,1].

As such cuspless sub-Riemannian geodesics provide a suitable alternative to (involved and not necessarily optimal) elastica curves in computer vision. Moreover, they seem to provide a very adequate model for association fields and they are the solutions to Petitot’s circle bundle model. They also relate to previous models for association fields based on horizontal exponential curves (i.e. “co-circularity”) via the Cartan connection: Along horizontal exponential curves tangent vectors are parallel transported, whereas along sub-Riemannian geodesics momentum is parallel transported.

Our solutions, analysis and geometric control for the sub-Riemannain geodesics presented in this article form the venture point for data-dependent active contour models in SE(2) (in combination with contour-enhancement [1, 14, 22, 26, 29, 35, 36] and contour completion PDE’s [4, 8, 30, 48]) we are currently developing and applying in various applied imaging problems. Applications include extraction of the vascular tree in 2D-retinal imaging [10] and fiber-tracking in diffusion weighted magnetic resonance imaging [23, 62] (where we use sub-Riemannian geodesics in SE(3) solving the 3D-version of **P**_curve_). In these applications one replaces the constant measure on SE(2) in **P**_curve_ by a data-dependent measure $\tilde{C}:\mathrm{SE}(2) \to[1,\infty)$ in **P**_curve_, producing external force terms in the Euler-Lagrange equations that pull the geodesics towards the data.

Finally, future work will include comparison of numerical algorithms for **P**_MEC_ and **P**_curve_.

## Appendix A: Basic Derivation of the ODE for Curvature Along Sub-Riemannian Geodesics

We derive normalized curvature $z=\frac{\kappa}{\sqrt{\kappa^{2}+\xi^{2}}}$, recall Eq. (77), along stationary curves of the functional

$$E(\mathbf{x})=\int_{0}^{\ell} \sqrt{ \kappa^{2}(s)+\xi^2 }\, {\rm d} s, $$

with $\mathbf{x}:[0,\ell] \to\mathbb{R}^{2}$ twice differentiable. Here we apply Mumford’s approach to elastica [48] to cuspless sub-Riemannian geodesics instead.

The energy after horizontal curve deformation

$$\mathbf{x} \mapsto\mathbf{x}_{NEW}:=\mathbf{x} +h \delta\mathbf{n} $$

with *h*>0 and $\mathbf{n}(s)=\ddot{\mathbf{x}}(s)$, $\delta :[0,\ell] \to \mathbb{R}$ infinitely differentiable and vanishing at the boundary (i.e. $\delta\in\mathcal{D}([0,\ell])$), becomes

$$\begin{aligned}& E(\mathbf{x} +h\delta\mathbf{n})\\ & \quad =\int_{0}^{\ell_{NEW}} \sqrt{\kappa_{NEW}^{2}(s) +\xi^{2} } \,{\rm d}s_{NEW} \\ & \quad = \int_{0}^{\ell} \sqrt{\kappa^{2} +2 h\delta'' \kappa+ 2 \delta h \kappa^{3} + \xi^{2} +O(h^{2})} (1-\delta h \kappa) {\rm d}s \\ & \quad = \int_{0}^{\ell}\sqrt{\kappa^{2}+\xi^{2}}\sqrt{1+\frac {2h\delta'' \kappa+2 h\delta\kappa^3}{\kappa^{2} +\xi ^{2}}}(1-h\delta\kappa){\rm d}s \\ & \quad = \int_{0}^{\ell}\sqrt{\kappa^{2}+\xi^{2}} (1+ \frac {h\delta'' \kappa+ h\delta\kappa^3}{\kappa^{2} +\xi^{2}} + O(h^{2}) )(1-h\delta\kappa){\rm d}s \\ & \quad = \int_{0}^{\ell}\sqrt{\kappa^{2}+\xi^{2}} \biggl(1+ \frac {h\delta'' \kappa+ h\delta\kappa^3}{\kappa^{2} +\xi^{2}} -\delta \kappa+ O(h^{2}) \biggr){\rm d}s \\ & \quad = E(\mathbf{x}) + h \int_{0}^{\ell} \sqrt{\kappa^{2}+\xi^{2} } \biggl( \frac{\delta'' \kappa+\delta\kappa^{3}}{\kappa^2 +\xi ^{2}}-\delta\kappa\biggr) {\rm d}s +O(h^2) \end{aligned}$$

where we used $\sqrt{1+x}=1 +\frac{1}{2} x + O(x^{2})$ and

75 $$ \left\{ \begin{array}{l} {\rm d}s_{NEW}\equiv(1-h \delta\kappa){\rm d}s \\ \mathbf{t}_{NEW}=\frac{d\mathbf{x}_{NEW}}{ds_{NEW}}=\frac {ds}{ds_{NEW}}\frac{d \mathbf{x}_{New}}{ds} \equiv\mathbf{t} +h\delta' \mathbf{n}\\ \kappa_{NEW}= \frac{d \mathbf{t}_{NEW}}{ds_{NEW}} \cdot\mathbf {n}_{NEW} \equiv\kappa+h\delta'' +h\delta\kappa^{2} +O(h^{2}), \\ \mathbf{n}_{NEW}= \frac{d^{2} \mathbf{x}_{New}}{ds_{NEW}^{2}} \equiv \mathbf{n} -h\delta' \mathbf{t} \end{array} \right. $$

Now $\mathcal{D}([0,\ell])$ is dense within $\mathbb{L}_{2}([0,\ell ])$ so for stationary curves one must have $\ddot{z}(s)=\xi z(s) \Leftrightarrow$

76 $$ \biggl(\frac{\kappa}{\sqrt{\kappa^2+\xi^{2}}} \biggr)'' +\frac {\kappa^{3}}{\sqrt{\kappa^{2}+\xi^{2}}} - \kappa\sqrt{\kappa ^{2}+\xi^{2}}=0. $$

This gives us the preservation law

$$\xi^{2}\bigl(1-|z(s)|^{2}\bigr) +|\dot{z}(s)|^{2}= \mathfrak{c}^2\xi^2:= \xi^{2} \bigl(1-|z(0)|^{2}\bigr) +|\dot{z}(0)|^{2} $$

and curvature $\kappa^{2}(s)= \frac{\xi^{2} (z(s))^{2}}{1-(z(s))^{2}}$ with

77 $$ z(s)= z_{0} \cosh(\xi s) + \frac{\dot{z}_0}{\xi} \sinh( \xi s), $$

with $z_{0}=\frac{\kappa_{0}}{\sqrt{\xi^{2} +\kappa_{0}^{2}}}$, $\dot{z}_{0}=\frac{\xi^{2} \dot{\kappa}_{0}}{(\xi^{2} + \kappa _{0}^{2})^{\frac{3}{2}}}$. These expressions are only valid for $s \in[0,s_{\mathrm{max}}(z_{0},\dot{z}_{0}))$ where

78 $$\begin{aligned} s_{\mathrm{max}}(z_0,\dot{z}_{0}) :=& \log\biggl(\frac{1+\mathfrak {c}}{|z_0+ \dot{z}_{0}|} \biggr) \\ =&\frac{1}{\xi} \log\Biggl( \frac{1+\sqrt{1- (z_0^{2}-(\xi ^{-1}\dot{z}_{0})^{2})}}{z_0 + \xi^{-1} \dot{z}_{0}} \Biggr). \end{aligned}$$

This last expression (78) denotes the spatial length towards a cusp (where *E* and the sub-Riemannian distance *d*, recall Eq. (16), remain finite despite the fact that curvature tends to ∞ when approaching a cusp).

## Appendix B: Explicit Expression of Geodesics in Terms of Elliptic Functions

We will restrict ourselves to the case *ξ*=1 as we recall from the introduction (Remark 1.2) that the general case follows by spatial scaling.

### B.1 The geodesics for **P**_curve_ parameterized by spatial arclength

For **P**_curve_ geodesics (**x**(*s*),*θ*(*s*)) are given by

79 $$ \begin{aligned} &\theta(s)=\tilde{\theta}(s) - \overline{\theta}_{0} \in[-\pi,\pi ], \quad\textrm{with} \\&\quad \overline{\theta}_{0}=\arg\biggl\{ \frac{1}{\mathfrak{c}}(\dot{z}_0-i \sqrt{1-|z_0|^2})\biggr\} , \\& \mathbf{x}(s)= \overline{R}_{0}^{T}\biggl(\biggl(\tilde{x}(s)-\frac {z_{0}}{\mathfrak {c}},\tilde{y}(s)\biggr)^{T}\biggr),\quad \textrm{with}\\&\quad \overline{R}_{0}^{T}= \frac{1}{\mathfrak{c}} \left( \begin{array}{c@{\quad}c} \dot{z}_{0} & - \sqrt{1-|z_{0}|^2} \\ \sqrt{1-|z_0|^2} & \dot{z}_{0} \end{array} \right), \\&\quad \tilde{x}(s)= \frac{z(s)}{\mathfrak{c}}, \quad\quad \tilde{y}(s)= -\frac{1}{\mathfrak{c}} \int _{0}^{s} \sqrt{1-|z( \tau)|^2}\, {\rm d}\tau, \\&\quad \tilde{\theta}(s)= \arg\bigl(\dot{z}(s) - i \sqrt{1-|z(s)|^2} \bigr), \end{aligned} $$

with $z(s)=z_{0} \cosh(s) + \dot{z}_{0} \sinh(s)$, *z*_0_∈[−1,1], $\dot{z}_{0} \in\mathbb{R}$ and $\mathfrak{c}=\sqrt{1-|z_{0}|^{2}+|\dot{z}_{0}|^{2}}$. Geodesics are defined for *s*∈[0,*s*_*max*_], with $s_{max}=\log(\frac {1+\mathfrak{c}}{|z_{0}+ \dot{z}_{0}|} )$.

#### Remark

Lemma 4 expresses the integral for $\tilde{y}$ in a singe elliptic function.

### B.2 The Geodesics for **P**_MEC_ (and **P**_curve_) Parameterized by Sub-Riemannian Arclength

Here we distinguish between different cases in the phase portrait of the mathematical pendulum, recall Fig. 9.

In the cases $C_{1}=C_{1}^{1} \cup C_{1}^{0}$ (where $\mathfrak{c}<1$), $C_{2}=C_{2}^{+} \cup C_{2}^{-}$ (where $\mathfrak{c}>1$), the geodesics of **P**_MEC_ are expressed [47] in sub-Riemannian arc-length *t* are parameterized by Jacobian functions $\mathrm{cn\,}$, $\mathrm{sn\, }$, $\mathrm{dn\,}$, ${\mathrm{E\,}}$ as follows.

Case *C*_1_:

$$\begin{aligned} &\cos\theta(t) = \mathrm{cn\,}\varphi\mathrm{cn\,}(\varphi+t) + \mathrm{sn\,}\varphi\mathrm{sn\,}(\varphi+t), \\ &\sin\theta(t) = \pm\bigl(\mathrm{sn\,}\varphi\mathrm{cn\, }(\varphi+t) - \mathrm{cn\,}\varphi\mathrm{sn\,}(\varphi+t) \bigr), \\ &x(t) = (\pm/k) \bigl[ \mathrm{cn\,}\varphi\bigl(\mathrm{dn\, }\varphi- \mathrm{dn\,}(\varphi+t)\bigr) \\ &\phantom{x(t) =}{}+ \mathrm{sn\,}\varphi\bigl(t + {\mathrm{E\,}}(\varphi) - {\mathrm{E\,}}(\varphi+t)\bigr)\bigr], \\ &y(t) = (1/k) \bigl[ \mathrm{sn\,}\varphi\bigl(\mathrm{dn\, }\varphi- \mathrm{dn\,}(\varphi+t)\bigr) \\ &\phantom{y(t) =}{}- \mathrm{cn\,}\varphi\bigl(t + {\mathrm{E\,}}(\varphi) - {\mathrm{E\,}}(\varphi+t)\bigr)\bigr]. \end{aligned}$$

Case *C*_2_:

$$\begin{aligned} &\cos\theta(t) = k^2 \mathrm{sn\,}(\varphi/k) \mathrm{sn\, }(\varphi+ t)/k \\ &\phantom{\cos\theta(t) =}{} + \mathrm{dn\,}(\varphi/k) \mathrm{dn\,}(\varphi+ t)/k, \\ &\sin\theta(t) = k\bigl(\mathrm{sn\,}(\varphi/k) \mathrm{dn\, }(\varphi+ t)/k \\ &\phantom{\sin\theta(t) =}{} - \mathrm{dn\,}(\varphi/k) \mathrm{sn\,}(\varphi+ t)/k\bigr), \\ &x(t) = \pm k \bigl[\mathrm{dn\,}(\varphi/k) \bigl(\mathrm{cn\, }(\varphi/k) - \mathrm{cn\,}(\varphi+ t)/k \bigr) \\ &\phantom{x(t) =}{}+ \mathrm{sn\,}(\varphi/k) (t/k + {\mathrm{E\,}}(\varphi/k) - {\mathrm{E\,}}\bigl((\varphi+ t)/k\bigr)\bigr], \\ &y(t) = \pm\bigl[k^2 \mathrm{sn\,}(\varphi/k) \bigl(\mathrm{cn\, }(\varphi/k) - \mathrm{cn\,}( \varphi+ t)/k\bigr) \\ &\phantom{y(t) =}{} - \mathrm{dn\,}(\varphi/k) \bigl(t/k + {\mathrm{E\,}}(\varphi/k) - {\mathrm{E\,}}(\varphi+ t)/k\bigr)\bigr]. \end{aligned}$$

In the critical case $C_{3}= C_{3}^{1+}\cup C_{3}^{1-} \cup C_{3}^{0-} \cup C_{3}^{0+}$ (where $\mathfrak{c}=1$) geodesics are parameterized by hyperbolic functions:

$$\begin{aligned} &\cos\theta(t) = 1/ \bigl(\cosh\varphi\cosh(\varphi+ t)\bigr) \\ &\phantom{\cos\theta(t) =}{} + \tanh\varphi\tanh(\varphi+ t), \\ &\sin\theta(t) = \pm\bigl(\tanh\varphi/\cosh(\varphi+ t) \\ & \phantom{\sin\theta(t) =}{}- \tanh(\varphi+ t) /\cosh\varphi\bigr), \\ &x(t) = \pm\bigl[(1/\cosh\varphi) \bigl(1/\cosh\varphi- 1/\cosh (\varphi+ t) \bigr) \\ &\phantom{x(t) =}{}+ \tanh\varphi\bigl(t + \tanh\varphi- \tanh(\varphi+ t)\bigr)\bigr], \\ &y(t) = \pm\bigl[\tanh\varphi\bigl(1/\cosh\varphi- 1/\cosh (\varphi+ t)\bigr) \\ &\phantom{y(t) =}{} -(1/\cosh\varphi) \bigl(t + \tanh\varphi- \tanh(\varphi+ t)\bigr)\bigr]. \end{aligned}$$

Here (*φ*,*k*) are action-angle coordinates in the state space of the pendulum Eq. (10) that rectify its flow: $\dot{\varphi}= 1$, $\dot{k} = 0$. Set *s*_1_=sign(cos(*ν*/2)), *s*_2_=sign(*c*)∈{−1,1}. Using Jacobi’s functions $\mathrm{sn\,}(\varphi,k), \mathrm{dn\, }(\varphi,k), \mathrm{cn\,} (\varphi,k)$ and elliptic integrals of the first kind *K*(*k*), the explicit dependence of (*φ*,*k*) on (*ν*,*c*), cf. [47], is given by

- Case (*ν*,*c*)∈*C*_1_:
  80 $$ \begin{array}{l} k=\sqrt{\sin^{2}(\nu/2)+ c^2} \in(0,1), \\ \sin(\nu/2)= s_{1} k \mathrm{sn\,}(\varphi,k), \\ \cos(\nu/2)= s_{1} \mathrm{dn\,}(\varphi,k), \\ c/2 = k \mathrm{cn\,}(\varphi,k), \varphi\in[0,4K(k)]. \end{array} $$
- Case (*ν*,*c*)∈*C*_2_:
  81 $$ \begin{array}{l} k=1/\sqrt{\sin^{2}(\nu/2)+ c^2} \in(0,1), \\ \sin(\nu/2)= s_{2} \mathrm{sn\,}(\varphi/k,k), \\ \cos(\nu/2)= s_{1} \mathrm{cn\,}(\varphi/k,k), \\ c/2 = (s_2/k) \mathrm{dn\,}(\varphi/k,k), \varphi\in[0,4K(k)]. \end{array} $$
- Case (*ν*,*c*)∈*C*_3_:
  82 $$ \begin{array}{l} k=1, \\ \sin(\nu/2)= s_{1}s_{2} \tanh(\varphi), \\ \cos(\nu/2)= s_{1}/ \cosh(\varphi), \\ c/2 = s_{2}/\cosh(\varphi) , \varphi\in\mathbb{R}. \end{array} $$

#### Remark 11.1

The geodesics of **P**_MEC_ are defined for $t \in \mathbb{R}$ and every pair of points in SE(2) can be connected by a smooth geodesic.

## Appendix C: The Cartan Connection and Its Relation to Geodesics and Exponential Curves

In this section we show that last three equations of the Pfaffian system Eq. (28) can be summarized in a single simple formula $\nabla_{\dot{\gamma}} p=0$, where ∇ denotes the Cartan connection on the co-tangent bundle *T* ^∗^(SE(2)) of SE(2).

In [26] we have shown that the principle fiber bundle *P*_*Y*_=(SE(2),SE(2)/*Y*,*π*,*R*) with projection *π*(*g*)=[*g*]=*gY*, *g*∈SE(2), base manifold SE(2)/*Y* and right-action *R*_*g*_(*h*)=*hg* and structure group $Y=\{(0,y,0)\mid y \in\mathbb{R}\}$, coincides with the sub-Riemannian manifold $(\mathrm{SE}(2),\Delta, \mathcal{G}_{\xi})$. We equip *P*_*Y*_ with Cartan-Maurer form *ω*=(*L*_0,−*y*,0_)^∗^ (i.e. the push-forward of the left-multiplication of the structure group acting on SE(2)). In our moving frame of reference this Lie-algebra-valued 1-form reads as

$$\omega_{g}(X_g) = \bigl\langle \omega^{3}\vert_{g}, X_{g}\bigr\rangle A_{3}. $$

for all left-invariant vector fields $X \in\mathcal{L}(\mathrm {SE}(2))$. By definition the horizontal part of the tangent bundle on SE(2) is given by

$$\mathcal{H}=\mathrm{ker}(\omega)=\mathrm{span}\{\mathcal{A}_{1}, \mathcal{A}_{2}\}, $$

which relates (*P*_*Y*_,*ω*) to the sub-Riemannian manifold $(\mathrm{SE}(2),\mathrm{Ker}(\omega^{3}),\mathcal{G}_{\beta})$.

The Maurer connection form *ω* induces the following connection form on the associated vector bundle $\mathrm{SE}(2) \times_{\widetilde{\mathrm{Ad}}} \mathcal {L}(\mathrm{SE}(2)) $

$$\tilde{\omega}= \widetilde{\mathrm{ad}}(\mathcal{A}_{3}) \otimes \omega^{3} =-\mathcal{A}_{2} \otimes\omega^{1} \otimes\omega^{3} $$

with $\widetilde{\mathrm{Ad}}={\rm d}\mathcal{R} \circ\mathrm{Ad} \circ\omega$ the adjoint action of SE(2) on the Lie algebra $\mathcal{L}(\mathrm{SE}(2))$ of left-invariant vector fields, whose push-forward equals $\widetilde{\mathrm {ad}}(A_{3})=[\cdot,A_{3}]= -\mathcal{A}_{2} \otimes\omega^{1}$, since $c_{13}^{2}=-1$.

Connection form $\tilde{\omega}$ induces the following matrix-valued 1-form

$$\tilde{\omega}^{j}_{k}(\cdot):=-\tilde{\omega}\bigl( \omega^{j},\cdot,\mathcal{A}_{k}\bigr), \quad k,j=1,2,3, $$

on the frame-bundle, cf. [59, pp. 353, 359], where the sections are moving frames.

Finally, the corresponding Cartan connection $D=d+\overline{\omega}$ on the tangent bundle *T*(SE(2)) with $\overline{\omega }(\sum_{k=1}^{3}a^{k} \mathcal{A}_{k})=a^{k} \sum_{j=1}^{3} \tilde {\omega }^{j}_{k}(\cdot) \mathcal{A}_{j}= a^{3} \omega^{1}(\cdot) \mathcal {A}_{2}$ and note that $\tilde{\omega}$ vanishes when restricted to $\mathcal{H}$ (as this implies *a* ^3^=0). Consequently, the only horizontal auto-parallel curves (i.e. $D_{\dot{\gamma}} \dot{\gamma}=0$) in *T*(SE(2)) passing through *g*_0_∈SE(2) at *t*=0 are given by

$$\gamma(t)=g_{0} e^{t (c^{1} A_{1} +c^{2} A_{2})}, $$

with *c* ^1^ and *c* ^2^ constant.

The construction: Cartan-Maurer form on principal fiber bundle → connection form on associated vector bundle → connection form on frame bundle → connection form on tangent bundle, can also be applied to structure group SE(2) acting on SE(2) from the right. Then we find *P*=(SE(2),SE(2)/SE(2)≡{*e*},*π*,*R*), *π*(*g*)=*e* with Cartan-Maurer form $\omega(X_{g})= \sum_{i=1}^{3} \langle\omega ^{i}\vert_{g}, X_{g}\rangle A_{i}$, and $\tilde{\omega}= \mathcal{A}_{2} \otimes\omega^{3} \wedge\omega ^{1} + \mathcal{A}_{3} \otimes\omega^{1} \wedge\omega^{2} $ and we obtain (for details see [26, Thm. 3.8])

### Definition 3

The Cartan connection ∇ on the tangent bundle (SE(2),*T*(SE(2))) is given by the covariant derivatives

83 $$\begin{aligned}& \nabla_{ X \vert_{\gamma(t)}}(\mu(\gamma(t))) \end{aligned}$$

84 $$\begin{aligned}& \quad:= \nabla \mu(\gamma(t))( X \vert_{\gamma(t)}) \end{aligned}$$

85 $$\begin{aligned}& \quad = \sum_{k=1}^{3} \dot{a}^{k}(t) \mathcal{A}_{k}(\gamma(t)) \end{aligned}$$

86 $$\begin{aligned}& \quad\quad{} + \sum_{k=1}^{3} a^{k}(\gamma(t)) \sum_{j=1}^{3} \tilde{\omega}^{j}_{k}( X \vert_{\gamma(t)}) \mathcal {A}_{j}(\gamma(t)) \end{aligned}$$

87 $$\begin{aligned}& \quad = \sum_{k=1}^{3} \dot{a}^{k}(t) \mathcal{A}_{k}(\gamma(t)) \end{aligned}$$

88 $$\begin{aligned}& \quad\quad{} + \sum_{i,j=1}^{3} \dot{\gamma}^{i}(t) a^{k}(\gamma (t)) \varGamma^{j}_{ik} \mathcal{A}_{j}(\gamma(t)), \end{aligned}$$

with $\dot{a}^{k}(t)= \dot{\gamma}^{i}(t) (\mathcal {A}_{i}\vert_{\gamma(t)} a^{k})$, for all tangent vectors $X \vert_{\gamma(t)} = \dot{\gamma }^{i}(t) \mathcal{A}_{i}\vert_{\gamma(t)}$ along a curve *t*↦*γ*(*t*)∈SE(2) and all sections $\mu(\gamma(t))=\sum_{k=1}^{3}a^{k}(\gamma(t)) \mathcal {A}_{k}(\gamma(t))$. The Christoffel symbols in (88) coincide with the structure constants of the Lie-algebra

89 $$ \varGamma^{j}_{ik}=-c^{j}_{ik}. $$

### Theorem 12

*Exponential curves are auto*-*parallel with respect to the Cartan connection* ∇. *Horizontal exponential curves are auto*-*parallel with respect to Cartan connection* *D*. *In fact*,

90 $$ \begin{aligned} &\nabla_{\dot{\gamma}}\dot{\gamma}=0\quad \Leftrightarrow\\ &\quad\exists_{(c^{1},c^{2},c^{3}) \in\mathbb{R}^{3}}\forall_{t \in \mathbb{R}} \\ &\quad\gamma(t)= \gamma(0)\, \mathrm{Exp} (t(c^{1}A_{1}+c^{2}A_{2}+c^{3}A_{3}) ), \\ &D_{\dot{\gamma}} \dot{\gamma}=0 \quad\Leftrightarrow\\ &\quad\exists_{(c^{1},c^{2}) \in\mathbb{R}^{2}}\forall_{t \in\mathbb {R}}\\ &\quad\gamma(t)= \gamma(0)\, \mathrm{Exp} (t(c^{1}A_{1}+c^{2}A_{2}) ), \end{aligned} $$

*where* Exp:*T*_*e*_(SE(2))→SE(2) *denotes the exponential map from Lie algebra to Lie group*. *Along an exponential curve* $\gamma (t)=\mathrm{Exp} (t \sum_{i=1}^{3} c^{i} A_{i} ) \gamma(0)$, *tangent vectors are covariantly constant*. *Along a geodesic one has covariantly constant momentum*, *i*.*e*.

91 $$ \nabla_{\dot{\gamma}} p=0. $$

### Proof

With respect to the first part of the theorem, we note that the left-invariant vector-fields on SE(2) are obtained by *T*_*e*_(SE(2)) by means of the infinitesimal generator of the right-regular representation acting on $C^{1}(\mathrm{SE}(2),\mathbb {R})$ via

$$\begin{aligned} &\mathcal{A}_{i}={\rm d}\mathcal{R}(A_{i})=\lim_{t \downarrow 0} t^{-1}(\mathcal{R}_{e^{tA_{i}}}-I),\quad\textrm{with } \\ &\quad\mathcal{R}_hU(g)=U(gh). \end{aligned} $$

Via the identity $e^{t {\rm d}\mathcal{R}(c^{i}A_{i})}=\mathcal{R}_{e^{\sum _{i=1}^{3}t(c^{i}A_{i})}}$ and application of the method of characteristics to linear, left-invariant convection systems on $C^{1}(\mathrm{SE}(2),\mathbb{R})$

$$\left\{ \begin{array}{l} \frac{\partial W}{\partial t}(g,t)=-\sum_{i=1}^{d} c^{i} (\mathcal{A}_{i}W)(g,t)\\ W(g,0)=U(g) \end{array} \right. $$

with *d*∈{2,3}, we find its unique solutions $W(g,t)=\mathcal{R}_{e^{-\sum_{i=1}^{d}t(c^{i}A_{i})}}(U)(g)= U(g e^{-t\sum_{i=1}^{d}c^{i}A_{i}})$. Thereby, for any *C* ^1^-curve $\gamma:\mathbb{R}\to\mathrm{SE}(2)$ one has

$$\bigl\langle \omega^{i} \vert_{\gamma(t)}, \dot{ \gamma}(t) \bigr\rangle =c^{i} \quad\Leftrightarrow\quad\gamma(t)=\gamma(0) e^{t\sum_{i=1}^{d}c^{i}A_{i}}. $$

Now, as the Christoffel symbols are anti-symmetric (see Eq. (89)) we have

92 $$ \nabla_{\dot{\gamma}} \dot{\gamma}=0 \quad\Leftrightarrow\quad \forall_{i=1,\ldots ,d}: \dot{\gamma}^{i}:=\frac{d}{dt}\bigl\langle \omega ^{i}\vert_{\gamma}, \dot{\gamma} \bigr\rangle =0, $$

which implies Eq. (90). With respect to Eq. (91) we note that by duality

93 $$\begin{aligned}& \sum_{i=1}^{d} \dot{\lambda}_{i}a^{i} + \lambda_{i} \dot {a}^{i} \\& \quad = \frac{d}{dt} \langle p, \mathcal{A}\rangle\vert_{\gamma(t)} \\& \quad= \langle\nabla_{\dot{\gamma}(t)} p\vert_{\gamma(t)}, \mathcal{A}\vert_{\gamma(t)} \rangle+ \langle p\vert_{\gamma(t)}, \nabla_{\dot{\gamma}(t)} \mathcal{A}\vert_{\gamma(t)} \rangle \end{aligned}$$

with $\mathcal{A}=\sum_{i=1}^{d}a^{i} \mathcal{A}_{i}$ and $p=\sum _{i=1}^{d}\lambda_{i} \omega^{i}$, covariant derivatives in the tangent bundle (Definition 3) induce covariant derivatives in the cotangent bundle (by inversion of the Christoffel symbols $c_{ji}^{k} \mapsto c_{ij}^{k}$):

94 $$ \nabla_{\dot{\gamma}(t)} p\vert_{\gamma(t)}= \sum _{j=1}^{d} \Biggl(\dot{ \lambda}_{j}(t) + \sum_{i,k=1}^{d}c_{ij}^{k} \dot{\gamma}^{i} \lambda_{k} \Biggr) \omega^{j}. $$

Now Eq. (94) and Eq. (31) imply Eq. (91). □

## Appendix D: Canonical ODE’s via the Pontryagin Maximum Principle (PMP)

For details, proof and formal formulation of the Pontryagin maximum principle see [3]. Here we will apply the principle to our problems of interest (**P**_MEC_ and **P**_curve_), without giving the general formulation. Furthermore, we will not rely on Hamiltonian vector fields on the co-tangent bundle. For details see [3, 47], [31, Thm. 1 & App. A].

### D.1 Application of PMP to **P**_MEC_ After Squaring the Lagrangian and Constraining to Fixed Length Using *t*-Parametrization

Via the Cauchy-Schwartz inequality it can be shown that **P**_MEC_ is equivalent to the problem where one finds in the space of *L* ^∞^ controls $u(\cdot),v(\cdot):[0,\ell]\to\mathbb{R}$, the solution of

95 $$\begin{aligned} &(x(0),y(0),\theta(0))=(x_{in},y_{in},\theta_{in}), \\ &(x(T),y(T),\theta(T))=(x_{fin},y_{fin},\theta_{fin}) , \\ & \left( \begin{array}{c} \frac{dx}{dt}(t)\\ \frac{dy}{dt}(t)\\ \frac{d\theta }{dt}(t) \end{array} \right)=\tilde{u}(t) \left( \begin{array}{c} \cos(\theta(t)) \\ \sin(\theta(t)) \\ 0 \end{array} \right)+\tilde{v}(t) \left( \begin{array}{c} 0\\ 0\\ 1 \end{array} \right) \\ & \int_0^T\xi^2\tilde{u}(t)^2+\tilde {v}(t)^2~{\rm d}t \to\min\\ & \end{aligned}$$

where we constrain the total time *T* in such a way that the curve is parameterized by sub-Riemannian arclength: $\xi^{2} \tilde{u}^{2}(t) +\tilde{v}^{2}(t)=1$.

The “control dependent Hamiltonian” equals

$$h_{\tilde{\mathbf{u}}}(p)= -\frac{1}{2}\bigl(\tilde{u}^{2}+ \tilde{v}^{2}\bigr) +p_{1} \tilde{u}+ p_{2} \tilde{v} \cos\theta+ p_{3}u_{2} \sin\theta $$

which provides (maximized) Hamiltonian

$$H(p)= \frac{1}{2} \bigl( (p_{2}\cos\theta+p_{3} \sin \theta)^2 + (p_{1})^{2} \bigr). $$

Here momentum is expressed in fixed coordinates

$$p= p_{1} {\rm d}\theta+ p_{2} {\rm d}x + p_{3} { \rm dy}. $$

The vertical part of the canonical equations is now given by

$$\begin{aligned}& \dot{p}_{1}(t) = -p_{2}(t)p_{3}(t) \cos(2\theta) \\& \phantom{\dot{p}_{1}(t) =}{}+ \frac{1}{2}((p_{3}(t))^2-(p_{2}(t))^2 )\sin(2\theta), \\& \dot{p}_{2}(t) =0, \\& \dot{p}_{3}(t) =0, \end{aligned}$$

which may be re-expressed in moving coordinates (again using sub-Riemannian arclength parametrization)

$$\begin{aligned}& \dot{\lambda}_{1}(t) =- \lambda_{2}(t) \lambda_{3}(t), \\& \dot{\lambda}_{2}(t)= \lambda_{1}(t)\lambda_{3}(t), \\& \dot{\lambda}_{3}(t)= -\lambda_{1}(t)\lambda_{2}(t). \end{aligned}$$

This ODE-system can be expressed in spatial arc-length parametrization (before cusp situations, i.e. for initial condition (*x*_*in*_,*y*_*in*_,*θ*_*in*_)=(0,0,0) and end condition $(x_{in},y_{in},\theta_{in}) \in\mathcal{R}$) yielding

$$\begin{aligned}& \dot{\lambda}_{1}(s) =- \lambda_{3}(s), \\& \dot{\lambda}_{3}(s)= -\lambda_{1}(s), \\& \frac{d}{ds} (\lambda_{1}^{2}+\lambda_{2}^{2})= \frac{d}{ds} (\lambda_{2}^{2}+\lambda_{3}^{2})=0, \end{aligned}$$

which coincides with the canonical equation for **P**_curve_.

The horizontal part of the PMP ODE is given right after Eq. (18) expressed in fixed coordinates, see also [16]. Expressed in our moving frame of reference, this horizontal part is given by $\dot{\gamma}=\sum_{i=1}^{2}\lambda_{i}\mathcal{A}_{i}\vert_{\gamma}$, see [31].

### D.2 Application of PMP to **P**_curve_ with free Length Problem Using *s*-Parametrization

When we apply PMP directly to **P**_curve_ and use spatial arc-length parametrization we obtain “control dependent Hamiltonian”

$$h_{u}(p)= p_{1} u_{1} +p_{2} \cos \theta+ p_{3} \sin\theta-\sqrt{1+u^2} $$

Optimization over all controls produces the (maximized) Hamiltonian

$$H(p)= p_{2} \cos\theta+p_{3} \sin\theta-\sqrt {1-p_{1}^{2}}=0, $$

which vanishes since the total length is free [3, Thm. 12.8]. As a result we find

$$\begin{aligned}& \dot{p}_{1}(s)=p_{2}(s) \sin\theta(s) -p_{3}(s) \sin\theta(s),\\& \dot{p}_{2}(s)=\dot{p}_{3}(s)=0, \end{aligned}$$

and thereby $\ddot{z}(s)=\ddot{\lambda}_{1}(s)=\ddot {p}_{1}(s)=p_{1}(s)=\lambda_{1}(s)=z(s)$ which coincides with the result in Appendix A.

## Appendix E: Proof of Theorem 9

First of all, see Fig. 11 (top view along *θ*) for a graphical validation of the statement. Without loss of generality we assume *y*_*fin*_<0. The surface of geodesics departing from a cusp and the surface of geodesics ending at a cusp intersect each other only at *θ*_*fin*_=0 (cases *z*(*l*)=−*z*(0)=±1 and $\mathfrak{c}>1$) or at |*θ*_*fin*_|=*π* (cases *z*(*l*)=*z*(0)=±1 and $\mathfrak{c}>1$). This directly follows by the formula for *θ*_*fin*_ in Eq. (52). Next we show that if a geodesic starts with a cusp it will have *x*_*fin*_<2. This follows by the formula for *x*_*fin*_ in Eq. (52) where the second term vanished if |*z*_0_|=1 and where the first term is less than 2. For if *z*(*ℓ*)=−*z*(0)=±1 with $|\dot{z}_{0}|>1$ we have |*x*_*fin*_|<|*z*(*ℓ*)−1|≤2, whereas if $|\dot{z_{0}}|<1$ and *z*(*ℓ*)=*z*(0)=±1 we again have $\frac{x_{fin}}{2} \leq\frac {x_{fin}^{2}+\sin^{2}(\theta_{fin})}{2 x_{fin}} < 1$. More precisely, a geodesic starting with a cusp will satisfy Eq. (65), since the maximum value for *x*_*fin*_ under the condition |*z*_0_|=1 is obtained at |*z*(*ℓ*)|=1=|*z*_0_| and in these cases we find $|\dot {z}_{0}|=\frac{x_{fin}}{2}$ and $|y_{fin}|=-x_{fin} i E (i \, \mathrm{arcsinh}\, \frac{x_{fin}}{\sqrt{4-x_{fin}^{2}}}, 1-\frac {4}{x_{fin}^{2}} )$. Now assume Eq. (65) holds and consider a geodesic *γ*:[0,*ℓ*]→SE(2) and consider the corresponding trajectory in phase space and assume that the trajectory has $\mathfrak{c}<1$ and $\dot{z}_{0}<-z_{0}$, then $\mathrm{sign}(z(s))=\mathrm{sign}(\kappa(s))=\mathrm {sign}(\dot{\theta}(s))=-1$ is for all *s*∈[0,*ℓ*] and minimum value for |*θ*(*s*)| is obtained *s*=*ℓ*. We can extend this geodesic to [*s*_*min*_,*s*_*max*_] with |*z*(*s*_*min*_)|=1 and *s*_*min*_≤0. Then the extended geodesic starts and ends in a cusp and we have $\theta_{fin} \in[ \theta_{\mathrm{endcusp}}(x_{fin},y_{fin}), \theta_{\mathrm {begincusp}}(x_{fin},y_{fin})] \subset\mathbb{R}^{-}$ due to the monotonic decrease of *s*↦*θ*(*s*). This can be observed in Fig. 11 where all extended orbits in $\mathcal{C}_{1}^{0}$ go down in theta direction converging towards the *θ*-minimum at the blue surface at *s*↑*s*_*max*_.

For geodesics with $\mathfrak{c}>1$ the curvature $\kappa(s)=\dot {\theta}(s)$ can switch sign only once at *s*_*B*_ where *z*(*s*_*B*_)=0. By the assumption *y*_*fin*_≤0 we can restrict ourselves to the non-trivial case *z*_0_<0, $\dot{z}_{0}>0$ and $\mathfrak{c}>1$, i.e. ($(z_{0},\dot{z}_{0}) \in C_{2}^{+}$) where we assume *z*(*ℓ*)>0 in order to consider the non-straightforward case where the curvature switches sign. Again we extend the geodesic to [*s*_*min*_,*s*_*max*_] (starting from a cusp ending at a cusp). The extended geodesic’s angle initially decreases initially on [0,*s*_*B*_], but then by reflection symmetry it will gain more than the initial decrease during *s*∈[*s*_*B*_,*s*_*max*_]. Thereby, if a geodesics is within $\mathcal {C}_{2}^{+}$ its extension will stay in $\mathcal{C}_{2}^{+}$, see Fig. 11 (as it cannot cross the critical surface) and converge (upwards in *θ*) towards the blue surface (where geodesics end with a cusp) and we have *θ*_*fin*_∈[*θ*_endcusp_(*x*_*fin*_,*y*_*fin*_),*θ*_begincusp_(*x*_*fin*_,*y*_*fin*_)]. Conclusion, if the bound (65) holds $(x_{fin},y_{fin}) \in \mathbb{R} ^{2}$ can be connected both with a geodesic starting with a cusp (at (0,0) and a geodesic ending in a cusp at (*x*_*fin*_,*y*_*fin*_), then the end-angles of these curves determine the cone of reachable orientations. In case Eq. (65) does not hold, there are no geodesics starting from a cusp that reach (*x*_*fin*_,*y*_*fin*_) and in these cases the cone of reachable orientations is just bounded by the end-angles of two geodesics ending in a cusp (the two blue surfaces in Fig. 11).  □

## Appendix F: Proof of Theorem 6

Within this proof we will rely on previous results by Sachkov in [47, 55, 56]. As these works rely on sub-Riemannian arclength parametrization and the pendulum phase portrait (Fig. 9), we will do the same in this proof. To this end (via Lemma 1) we re-express domain and range of the exponential map $\widetilde {\mathrm{Exp}}$ of **P**_curve_ in this parametrization

$$\begin{aligned} C =& \{p_0 \in T^{*}_{e}(\mathrm{SE}(2)) \mid H(p_0) = \frac{1}{2} \textrm{ and }p_0 \neq\pm{\rm d}\theta\}\\ \equiv& \{(\nu,c)\mid\nu\in[-\pi,3\pi], c \in\mathbb{R}, (0,0)\neq(\nu ,c)\neq(2\pi,0)\}, \\ \mathcal{D} =& \{(p_0,t) \mid p_0 \in C \textrm{ and }0<t \leq t_{cusp}(p_0) \}, \\ \mathcal{R} =& \widetilde{\operatorname{Exp}}(\mathcal{D}). \end{aligned}$$

where $t_{cusp}(p_{0})=t(s_{max}(z_{0},\dot{z}_{0}))$ denotes sub-Riemannian arclength until the first cusp-time formally defined by

$$t_{cusp}(p_0)= \inf \bigl\{ t>0 \mid\dot{x}(t)=\dot{y}(t)=0\bigr\} . $$

The function *p*_0_↦*t*_*cusp*_(*p*_0_) is continuous on *C* and it is a uniform lower bound [55] for the continuous function *p*_0_↦*t*_*cut*_(*p*_0_) which assigns to each initial momentum the corresponding time where global optimality of **P**_MEC_ is lost along the corresponding stationary curve.

Now that the preliminaries are done, let us start with the proof.

We will first show the mapping $\widetilde{\mathrm{Exp}}: \mathring {\mathcal {D}} \to\mathring{\mathcal{R}}$ is a diffeomorphism. Consider to this end the set $\check{N} = \{(p_{0},t) \in C \times\mathbb{R}^{+} \mid t < t_{cut}(p_{0})\}$. Since the function *t*_*cut*_:*C*→(0,∞] is continuous, the set $\check{N}$ is open. Let $\widetilde{\mathrm{EXP}}$ denote the exponential map of **P**_MEC_. It follows from Th. 3.1 [55] that the mapping $\widetilde{\mathrm{EXP}}\vert_{\check{N}}$ is injective. Moreover, it was shown in Th. 2.5 [56] that this mapping is a local diffeomorphism. Thus the restriction $\widetilde{\mathrm{EXP}}\vert_{\check{N}}$ is a global diffeomorphism. Now since *t*_*cusp*_(*p*_0_)≤*t*_*cut*_(*p*_0_), we have $\mathring{\mathcal{D}} \subset\check{N}$. So $\widetilde{\mathrm{Exp}}\vert_{\mathring{\mathcal{D}}}\equiv \widetilde{\mathrm{EXP}}\vert_{\mathring{\mathcal{D}}}$ is a global diffeomorphism as well.

Regarding the homeomorphic property we note that injectivity of $\widetilde{\mathrm{EXP}}\vert_{\check{N}}$ implies injectivity of $\widetilde{\mathrm{Exp}}\vert_{\check{N} \cap\mathcal{D}}\equiv\widetilde{\textrm{EXP}}\vert_{\check{N} \cap\mathcal{D}}$. Moreover, we have

$$\begin{aligned} &\mathcal{D} \setminus\check{N}=S_{1} \cap S_{2}, \quad\textrm{with }\\ &\quad S_{1}=\{(\nu,c,t) \in C \times\mathbb{R}^{+} \mid\nu=0,c \in(0,2), t=2K\}, \\ &\quad S_{2}=\{(\nu,c,t) \in C \times\mathbb{R}^{+} \mid\nu=0, c \in(-2,0), t=2K\}, \end{aligned}$$

where *K*=*K*(*k*) denotes the elliptic integral of the first kind (recall Appendix B). Now its was shown in [56] that the exponential map restricted to *S*_*k*_ map *S*_*k*_, for *k*=1,2, diffeomorphically onto the corresponding ranges. Now as these ranges are disjoint, we have that $\widetilde{\mathrm{Exp}}\vert_{\mathcal{D}}$ is bijective, with a continuous inverse.

Regarding the final remark: Consider a sequence $(g_{n})_{n \in\mathbb{N}}$ in $\mathcal{R}$ that converges to some $g \in\mathrm{SE}(2) \setminus\mathring{\mathcal{R}}$. We must show $g=(x,y,\theta) \in\mathfrak{l}$ or *g* is the end-point of a geodesic starting with a cups or *g* is the end-point of a geodesic ending at a cusp.

By the homeomorphic and diffeomorphic properties of $\widetilde {\mathrm{Exp}}$ this sequence relates to a sequence in the phase portrait converging to a point $(\nu,c,t) \in\partial\mathcal{D}$. Let us consider all possible cases:

- If (*ν*,*c*)=(0,0) or (*ν*,*c*)=(2*π*,0) we have $g \in\mathfrak{l}$.
- If (*ν*,*c*)=(0,*c*) with *c*>0 or if (*ν*,*c*)=(2*π*,*c*) with *c*<0 (i.e. the cases corresponding to the red lines in the phase portrait in Fig. 11b), this means *g* is the end-point of a geodesic starting with a cusp.
- If (*ν*,*c*)=(0,*c*) with *c*>0 or if (*ν*,*c*)=(2*π*,*c*) with *c*<0 (i.e. the cases corresponding to the blue lines in the phase portrait in Fig. 11b), this means *g* is the end-point of a geodesic ending with a cusp.

## Appendix G: Definition of Cusps, Geodesics and Association Field (Models)

We use the following definition of a geodesic.

### Definition 4

A geodesic of **P**_curve_ (respectively **P**_MEC_) is a stationary curve *γ* of the corresponding geometric control problem formulated in Sect. 1.1. Geodesics of **P**_curve_ are called cuspless sub-Riemannian geodesics.

### Remark

It can be shown that such a geodesic *γ* has the property that for every sufficiently small interval *I*=(*t*_1_,*t*_2_) in the domain of such a curve the restriction *γ*|_*I*_ is a minimizer between *γ*(*t*_1_) and *γ*(*t*_2_).

Smooth sub-Riemannian geodesics in **P**_MEC_ may exhibit cusps when projected to the spatial plane, as we recall from Fig. 2. Roughly speaking, cusps are singular points in which spatial velocity changes its sign.

### Definition 5

A curve trajectory $(\gamma(\cdot), \tilde{u}(\cdot), \tilde {v}(\cdot))$ has a cusp at *t*_*cusp*_∈[0,*T*] if $\tilde{u}(\cdot)$ changes its sign in a neighborhood of *t*_*cusp*_>0. More precisely, there exists an *ϵ*>0 such that $\tilde{u}(a)\tilde{u}(b)<0$ for all *a*∈(*t*_*cusp*_−*ϵ*,*t*_*cusp*_) and *b*∈(*t*_*cusp*_,*t*_*cusp*_+*ϵ*), where $\tilde{u}$ denotes the first control variable in **P**_MEC_. In such a case *γ*(*t*_*cusp*_) is called the cusp-point and *t*_*cusp*_>0 is called the cusp-time.

### Remark

Formally, in the minimizers of **P**_curve_, cusps do not occur, as the solutions break down at cusps. However, when |*z*(0)|=1, where *z*(*s*) denotes normalized curvature Eq. (5) we say that a geodesic in **P**_curve_ departs from a cusp. If |*z*(*ℓ*)|=1, i.e. $\ell=s_{max}(z_{0},\dot{z}_{0})$ given by Eq.(78), we say the geodesic of **P**_curve_ ends in a cusp.

### G.1 Association Field

The term association field comes from modeling contour integration in the human visual cortex by psycho-physical experiments [34, 52]. The general idea of an association field is to provide an *a priori* link between relative positions and orientations within the sensorium of cortical columns in the primary visual cortex, Fig. 5. Intuitively, the tangents to the field lines of the association field provide expected local orientations, given that an local orientation is observed at the center of the association field.

Field and his co-workers psychophysical measurements have resulted in the *association field* depicted in item a) of Fig. 8. They relied on the hypothesis that the visual system can solve the continuity problem separately at each scale.^14^

### G.2 Models of the Association Field

Within this article, we consider the cuspless sub-Riemannian geodesic model **P**_curve_, cf. [11, 22, 40], which is a natural extension of Petitot’s circle bundle model [52, Chap. 6.6.5]. Other models include Legendrian geodesics [52], and horizontal exponential curves in [57] given by Eq. (72).

These other models relate to the cuspless sub-Riemannian geodesics as follows:

- The Legendrian geodesics follow from the cuspless sub-Riemannian geodesic model by contracting (e.g. [25]) the sub-Riemannian manifold on $(\mathrm {SE}(2),\Delta ,\mathcal{G}_{\xi})$, Eq. (17), towards its nilpotent approximation, cf. Eq. (73).
- The horizontal exponential curves keeps the control variable in **P**_curve_ constant and they can be considered as a rough local approximation of sub-Riemannian geodesics, see item (c) in Fig. 8. Finally, we recall Theorem 12.

## Appendix H

We provide some estimates for the single elliptic integral appearing in the spatial arclength parametrization of cuspless sub-Riemannian geodesics, recall Theorem 3 and Lemma 4.

### Lemma 9

*We have the following lower and upper bound for the elliptic integral at* *s*=*s*_*max*_, *for the case* $z_{0}\dot{z}_{0}<1$ *and* $\mathfrak{c}<1$:

$$s_{B}\bigl(1+\sqrt{1-|z_0|^{2}}\bigr) \leq \int_{0}^{s_{max}} \sqrt{1-|z(s)|^{2}} {\rm d}s \leq s_{max}, $$

*with* *s*_*B*_ *such that* *z*(*s*_*B*_)=0 *and* |*z*(*s*_*max*_)|=1, *given by Eq*. (42) *and Eq*. (41). *A sharper upper*-*bound is given by*

$$\begin{aligned}& \int_{0}^{s_{max}}\sqrt{1-|z(s)|^{2}}{\rm d}s \\& \ \ \leq 2 (s_B-s^{*}) + \sqrt{1-|z_0|^2} s^* -\frac{1}{2} |s^{*}|^2 \frac{z_0\dot{z}_{0}}{\sqrt{1-|z_0|^{2}}} \\& \quad\ {} + (s_{max}-(s_{B}+s^{*})) + \frac{z_0\dot{z}_0}{\sqrt{1-|z_0|^{2}}} \frac{(s_{max}-(s_{B}+s^{*}))^2}{2}, \end{aligned}$$

*where*

$$\begin{aligned} s^*=\frac{\sqrt{1-|z_0|^2} -(1-|z_0|^2)}{-z_0 \dot{z}_{0}}. \end{aligned}$$

### Proof

The smooth integrand

$$\begin{aligned} s \mapsto\sqrt{1-|z(s)|^2} \end{aligned}$$

is a concave function, since

$$\begin{aligned} \frac{d^2}{ds^2} ( \sqrt{1-|z(s)|^{2}} )\leq0. \end{aligned}$$

Moreover, it is symmetric around the point *s*_*B*_ and we have 2*s*_*B*_≤*s*_*max*_. So the tangent lines at *s*=0 and *s*=2*s*_*B*_ to the graph of

$$\begin{aligned} s \mapsto \sqrt{1-|z(s)|^{2}} \end{aligned}$$

are contained in the epigraph. Their slope equals $\frac{-z_{0} \dot{z}_{0}}{\sqrt{1-|z_{0}|^{2}}}$. The rest follows by Fig. 18.  □

## Footnotes

1 More precisely, the models coincide for cuspless sub-Riemannian geodesics that can be properly parameterized by their *x*-coordinate.

2 This fact has more or less been overlooked in the previous literature on this topic.

3 Although not considered here the third approach also includes local optimality via Jacobi operators appearing in 2nd order variations [20, Chap. 4.1, Prop. 4.4].

4 The choice of *T*>0 does not change the set of minimizers, but only their parametrization. For this reason, it can be useful to choose a *T* such that the minimizer is parametrized by sub-Riemannian arclength.

5 In this case the Lagrangian and Hamiltonian relate to each other by the Fenchel transform on the Lie algebra of horizontal left-invariant vector fields akin to the 3D-case [30].

6 Usually the minimization in Eq. (16) is made in the space of Lipschitz functions, to guarantee the existence of minimizers via PMP. However, a posteriori one verifies that these minimizers are indeed *C* ^∞^.

7 For end-condition $(x_{fin},y_{fin},\theta_{fin}) \notin\mathcal{R}$ problem **P**_MEC_ has a minimizer with internal cusp (and thereby violating the natural settings of **P**_curve_). Such a minimizer of **P**_MEC_ can be approximated by smooth curves satisfying the constraints of problem **P**_curve_. In these cases **P**_curve_does not allow local or global minimizers, nor does it allow a stationary curve [16].

8 This becomes even more apparent when considering the *d*-dimensional extension of **P**_curve_, see [31].

9 For the sake of simplicity we do not index $\widetilde{\mathrm{Exp}}$ the exponential map with the initial condition *g*_*in*_, as throughout this article we set *g*_*in*_=*e*=(0,0,0).

10 As $\mathcal{D}$ and $\mathcal{R}$ are not open sets within the standard topologies on the embedding spaces $T_{e}(\mathrm{SE}(2)) \times \mathbb{R}^{+}$ and $\mathbb{R}^{2} \times S^{1}$. These subspace topologies do not coincide with the induced topology imposed by the embedding via the identity map, as such identity map is not continuous. However, with respect to the subspace topologies the set $\mathcal{D}$, respectively $\mathcal{R}$ are open sets and the homeomorphism $\widetilde{\mathrm{Exp}}: \mathcal{D} \to\mathcal{R}$ is well-defined.

11 The dual basis in (SE(2))_0_ is equal to $({\rm d}\theta, {\rm d}x,- \theta\, {\rm d}x+ {\rm d}y)$ and thereby the sub-Riemannian metric on (SE(2))_0_ does not include the |*y*′(*x*)|^2^ term.

12 The corresponding minimization problem (and induced sub-Riemannian distance) is left invariant in (SE(2))_0_ and not left-invariant in SE(2).

13 The preservation law and curvature in [52, Eq. (87)] does not fully match our results in [12, 26, 47, 55, 56], Appendix A and Theorem 2.
